# Supplementary figures and images for: Allosteric inhibition of the T cell receptor by a designed membrane ligand
Source: eLife. 2023 Oct 5;12:e82861. doi: 10.7554/eLife.82861 (PMC10554751; doi:10.7554/eLife.82861)

$\zeta$  (pY142)

| Marker | - | - | + | + | OKT3  |
|--------|---|---|---|---|-------|
|        | - | + | - | + | PITCR |

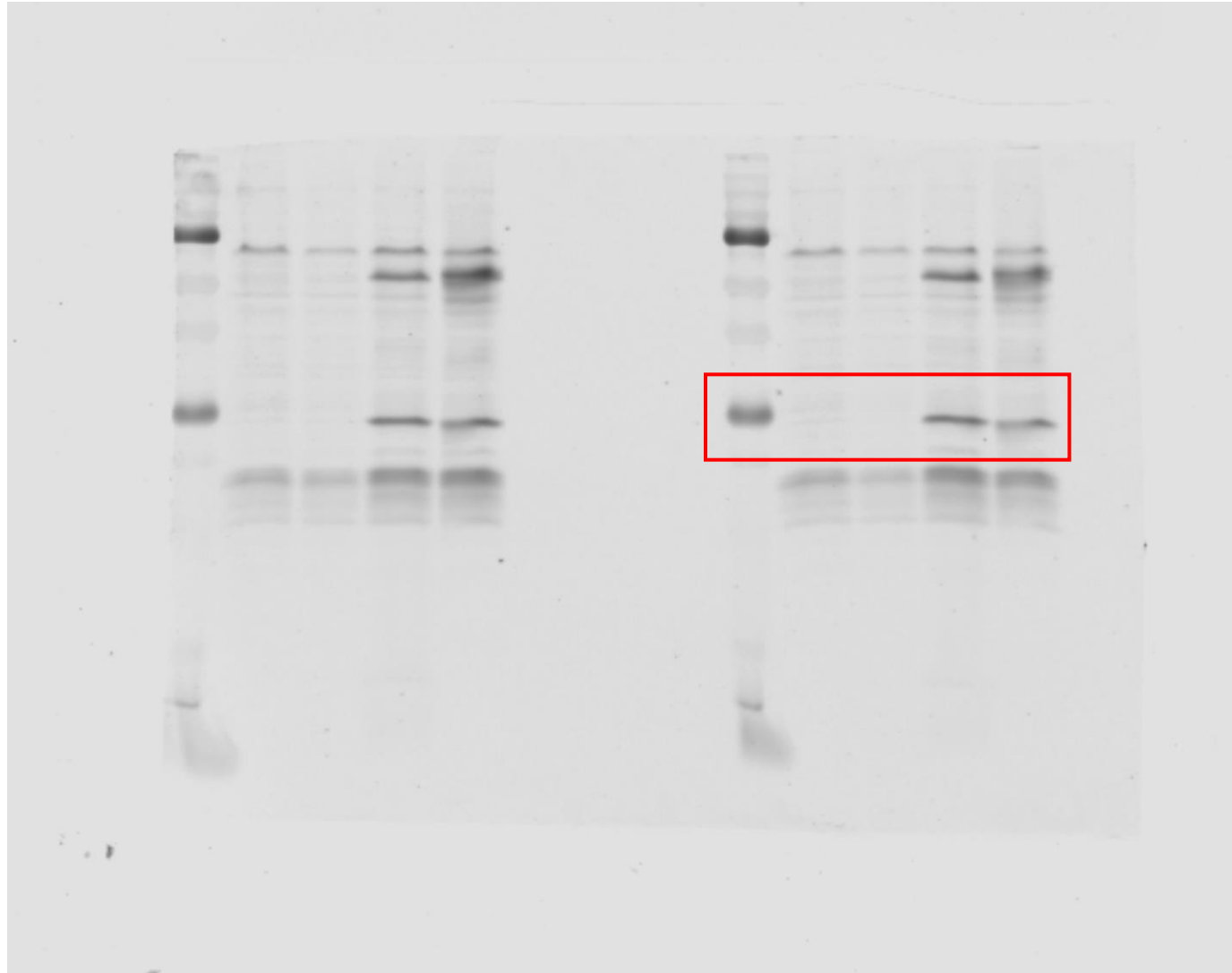

|                |        |   |   |   |   |       |
|----------------|--------|---|---|---|---|-------|
| $\zeta$ (pY83) | Marker | - | - | + | + | OKT3  |
|                |        | - | + | - | + | PITCR |

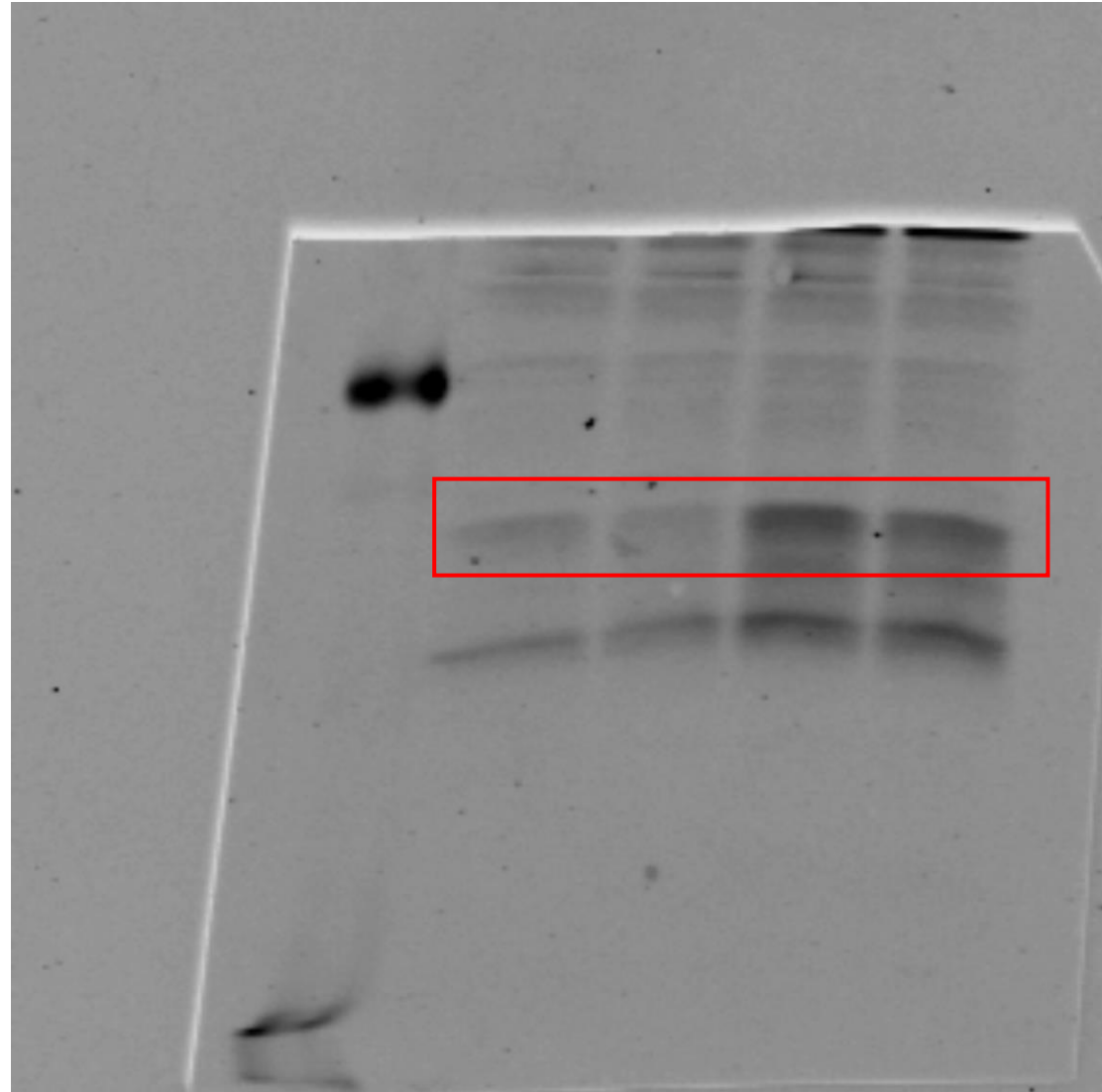

$\zeta$  (total)

|        |   |   |   |   |       |
|--------|---|---|---|---|-------|
| Marker | - | - | + | + | OKT3  |
|        | - | + | - | + | PITCR |

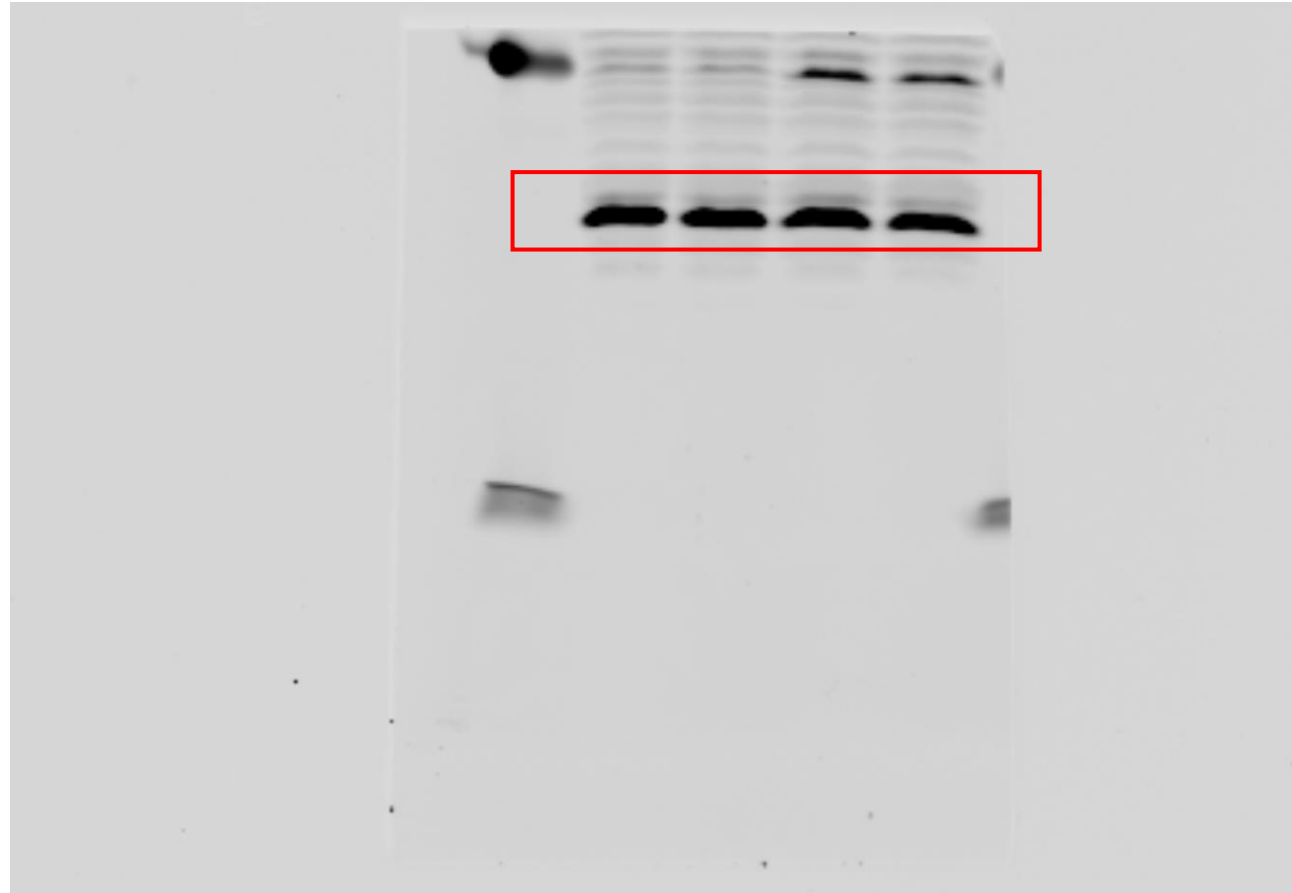

Supplement: Figure 1—source data 1. [file elife-82861-fig1-data1.zip › Figure1_labeled.pdf]

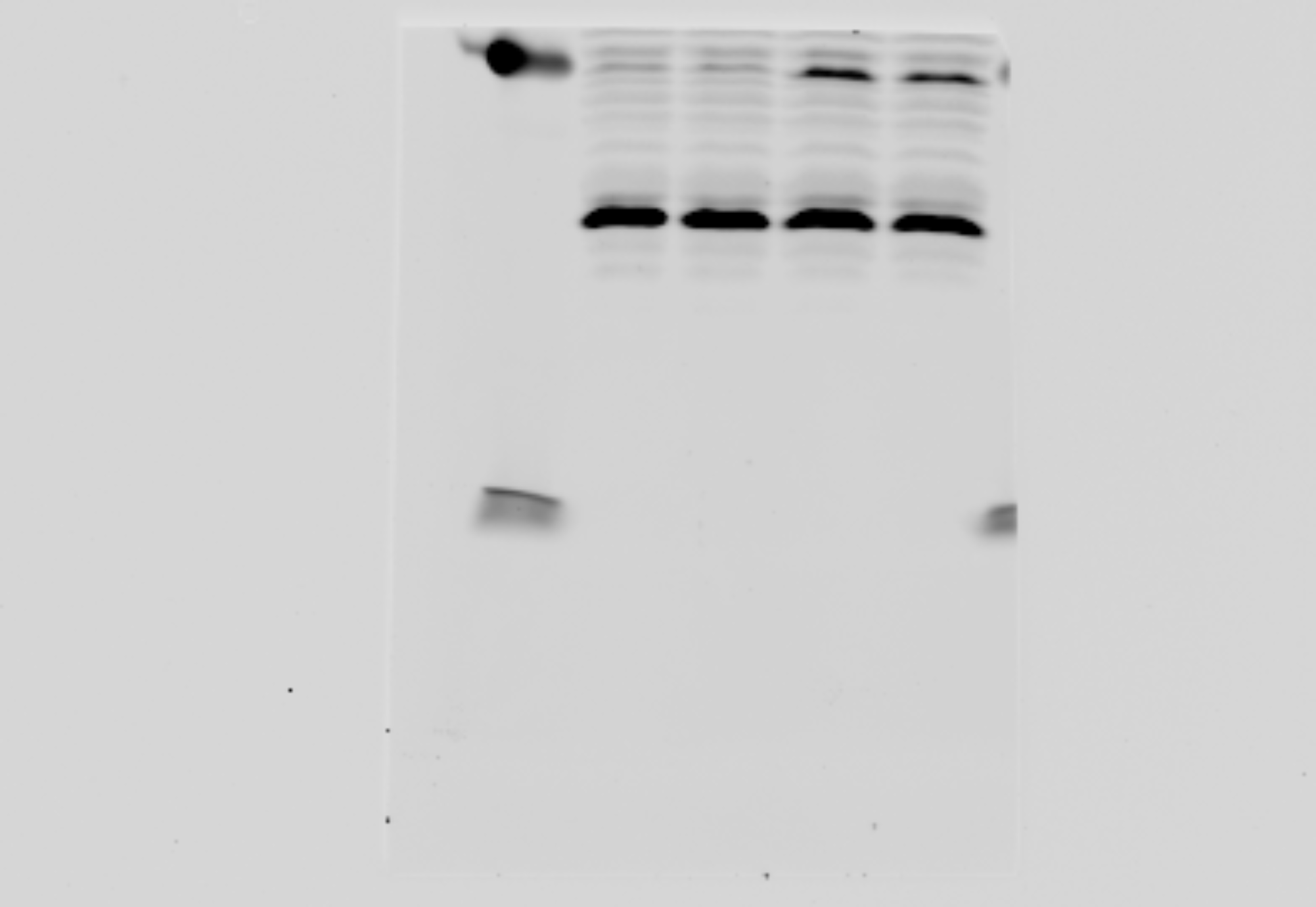

Supplement: Figure 1—source data 1. [file elife-82861-fig1-data1.zip › z-total.tif]

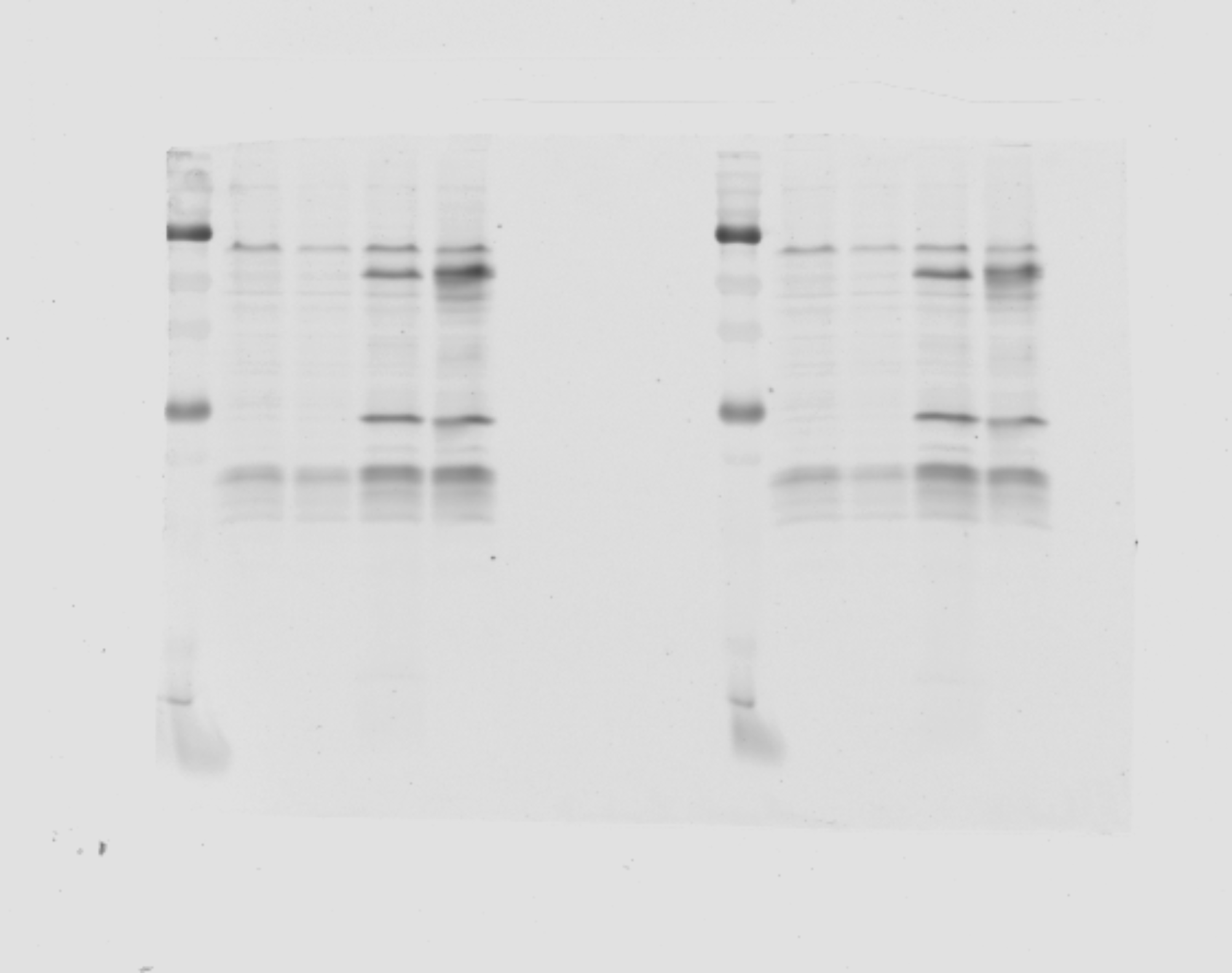

Supplement: Figure 1—source data 1. [file elife-82861-fig1-data1.zip › Z-Y142.tif.tif]

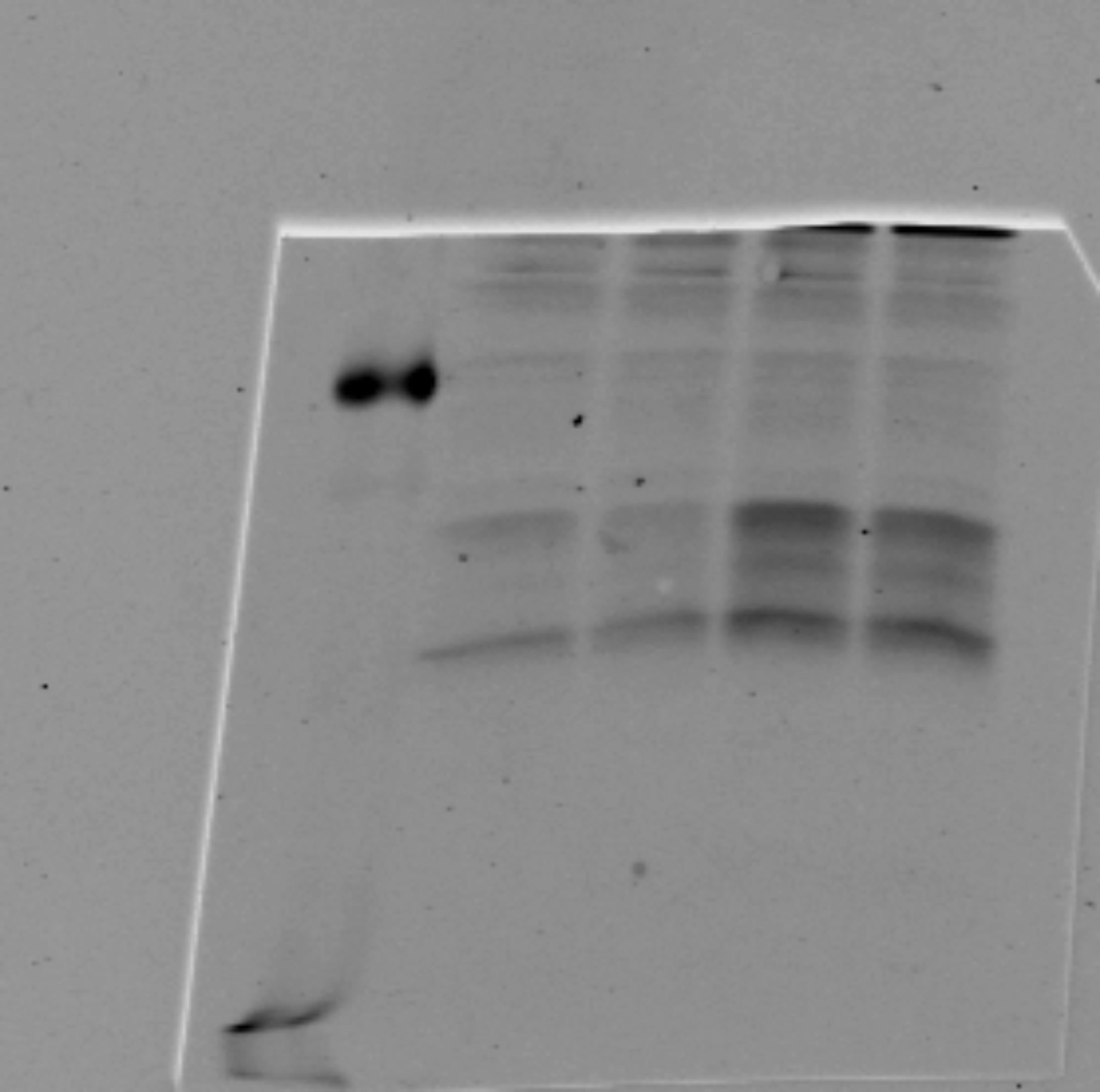

Supplement: Figure 1—source data 1. [file elife-82861-fig1-data1.zip › Z-Y83.tif.tif]

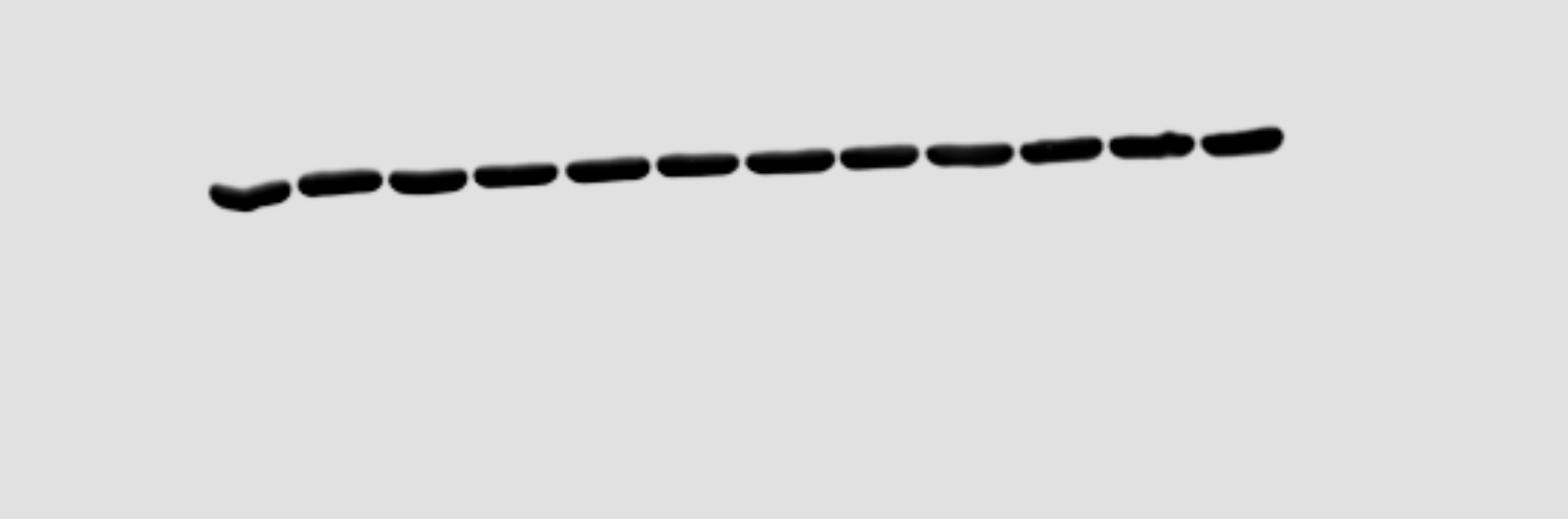

Supplement: Figure 2—source data 1. [file elife-82861-fig2-data1.zip › beta-actin.tif.tif]

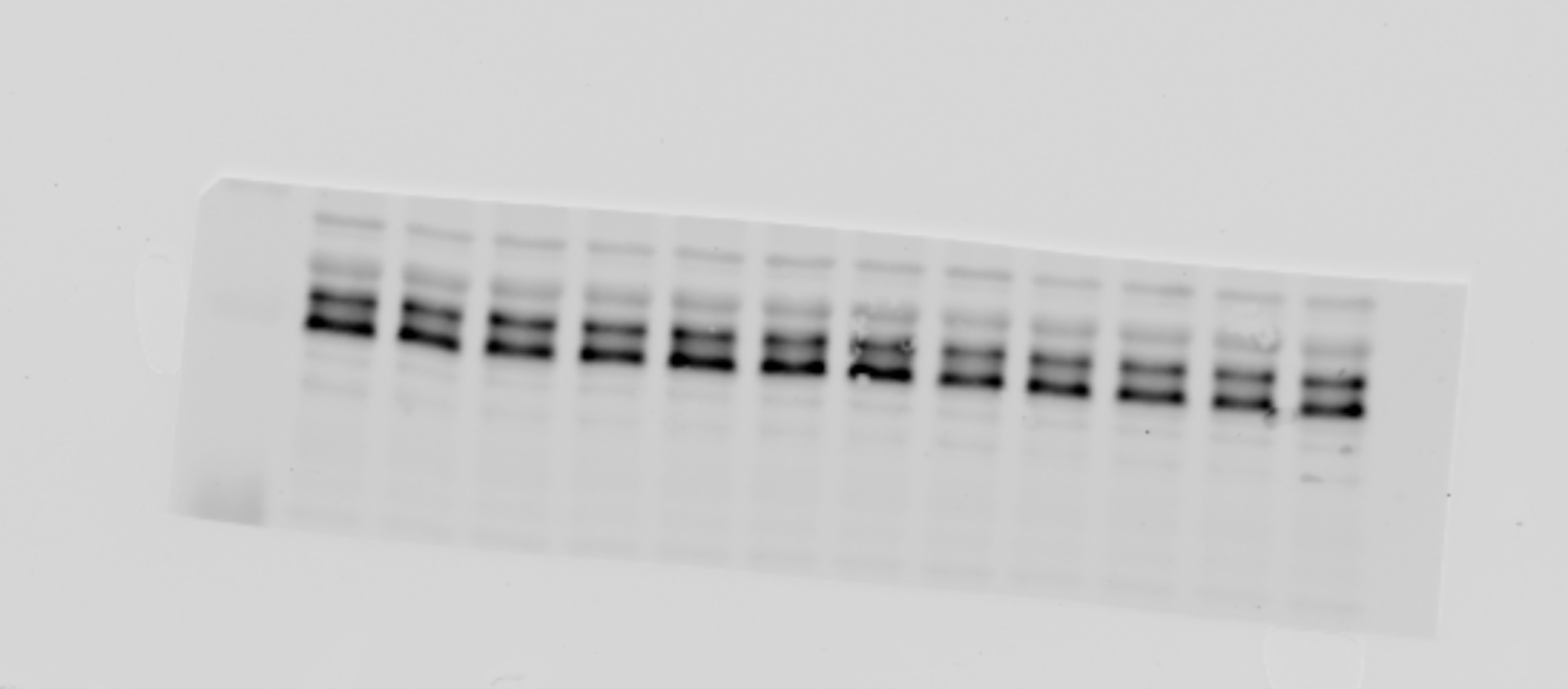

Supplement: Figure 2—source data 1. [file elife-82861-fig2-data1.zip › LAT-total.tif.tif]

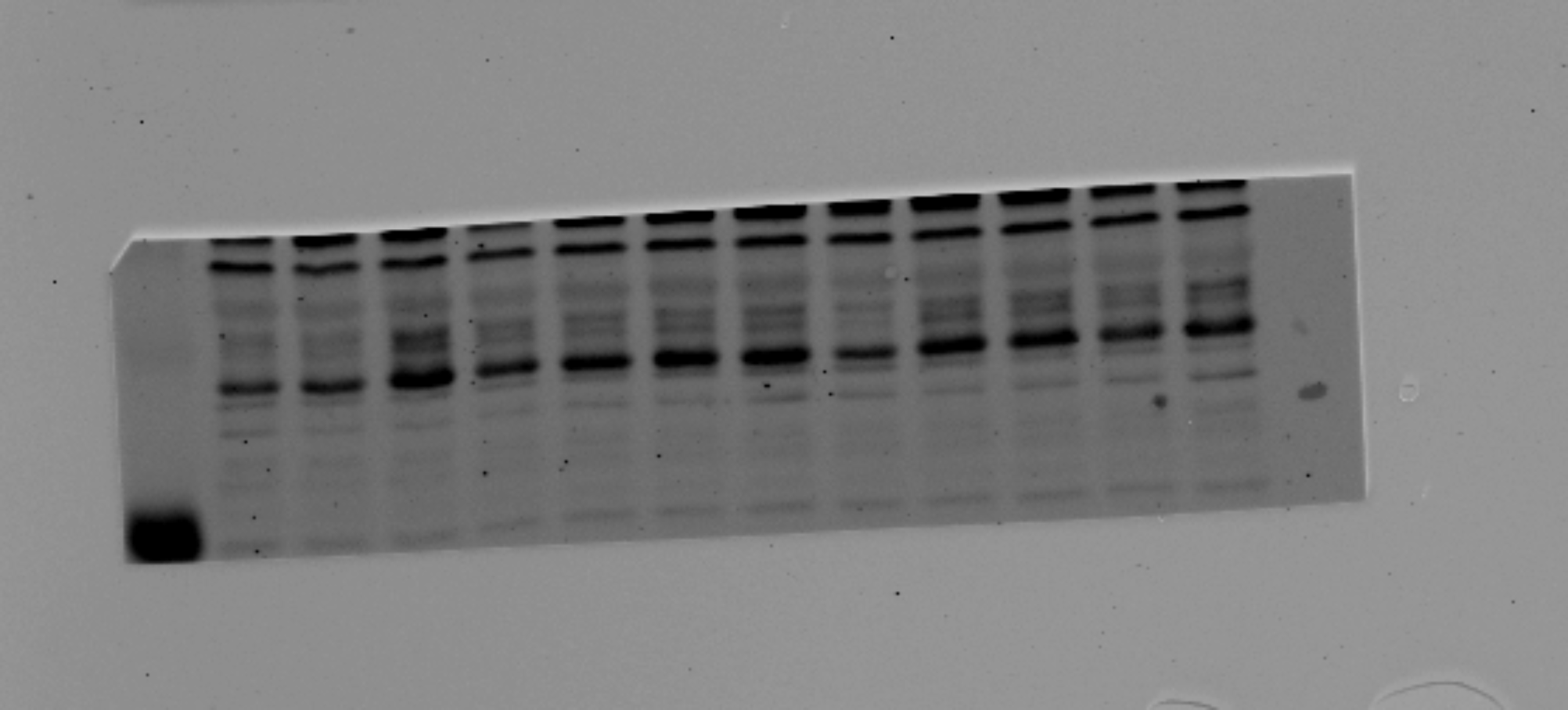

Supplement: Figure 2—source data 1. [file elife-82861-fig2-data1.zip › LATY132.tif.tif]

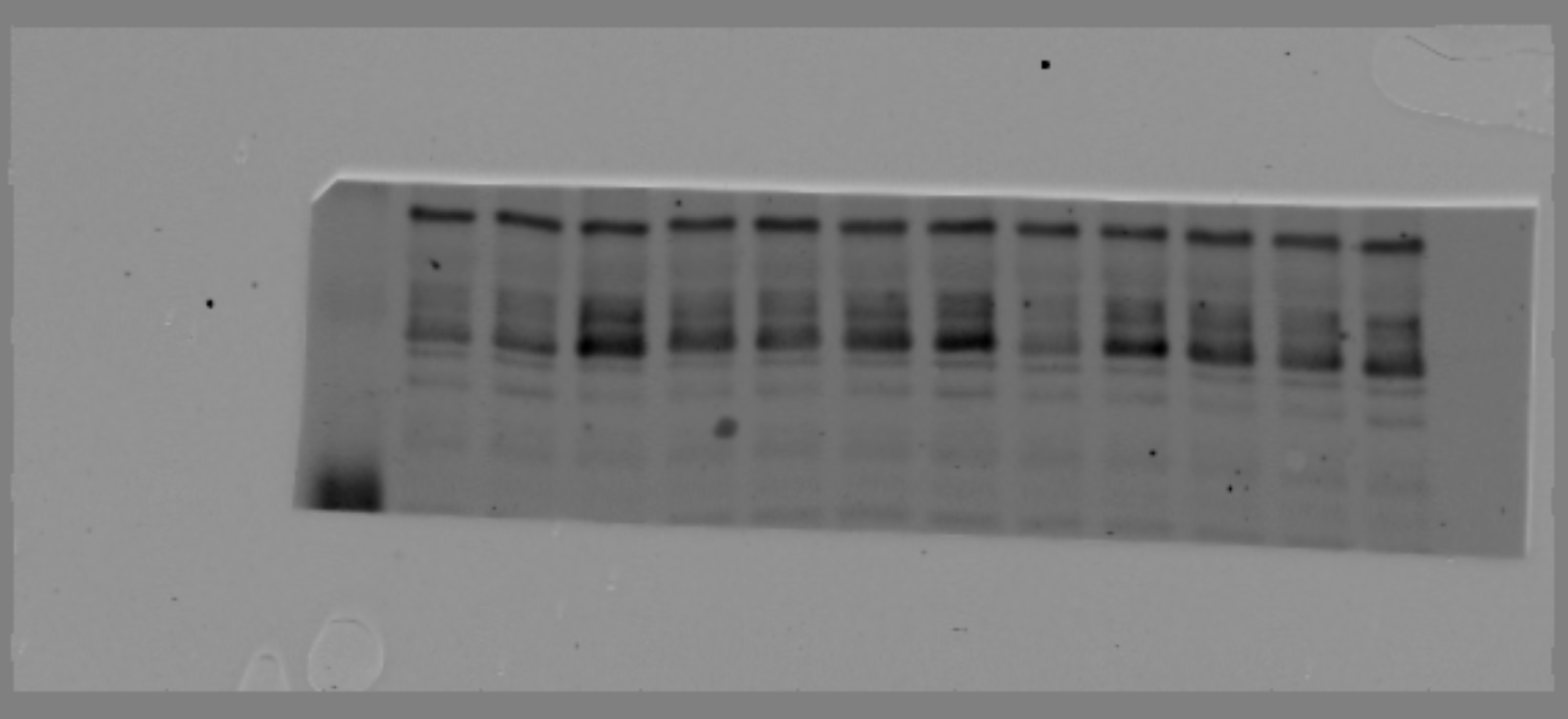

Supplement: Figure 2—source data 1. [file elife-82861-fig2-data1.zip › LATY191.tif.tif]

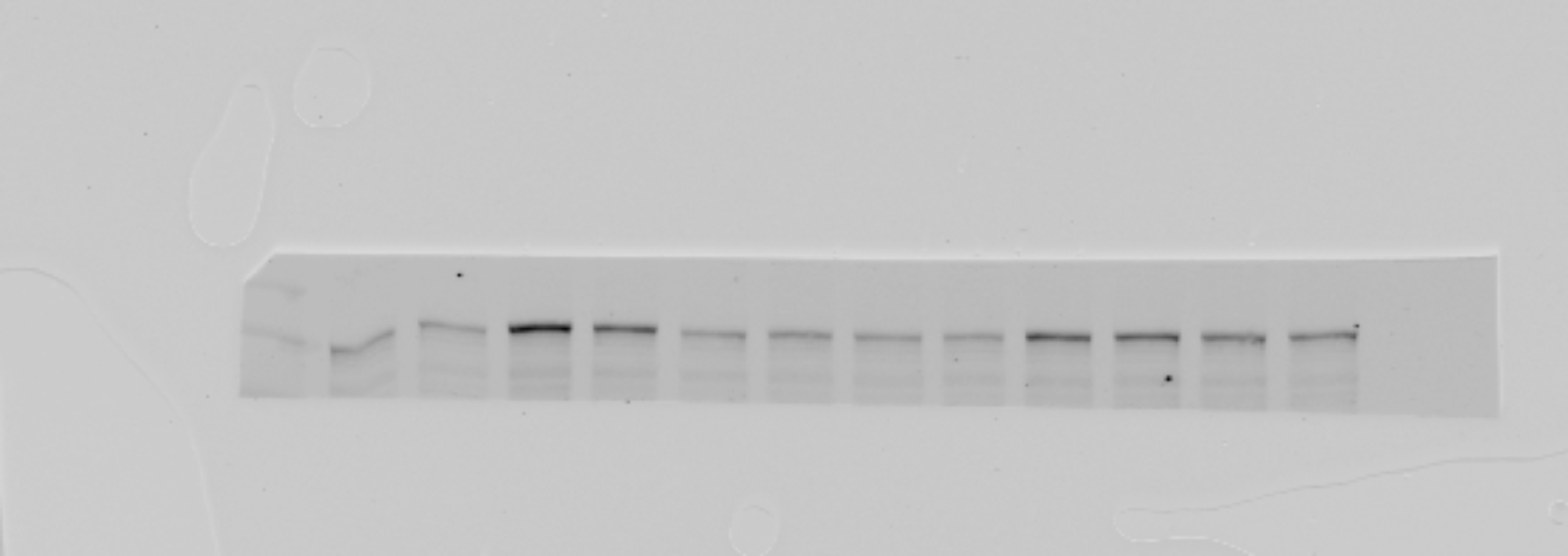

Supplement: Figure 2—source data 1. [file elife-82861-fig2-data1.zip › PLCg1Y783.tif.tif]

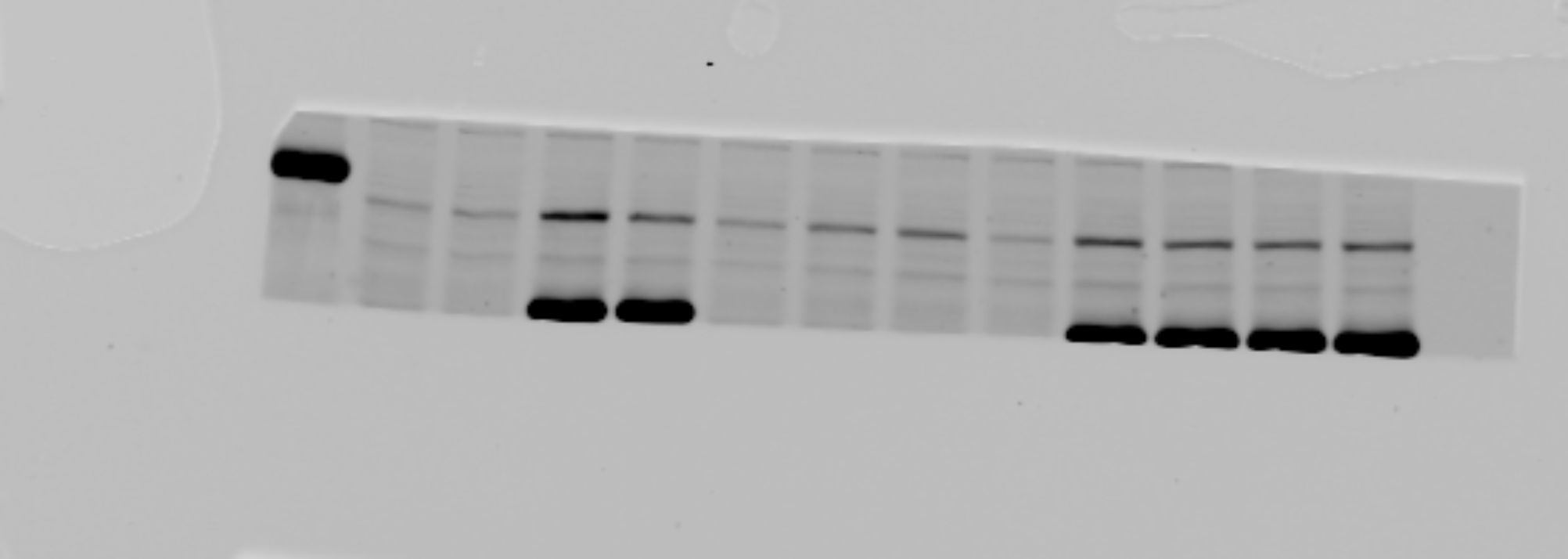

Supplement: Figure 2—source data 1. [file elife-82861-fig2-data1.zip › SLP76Y128.tif.tif]

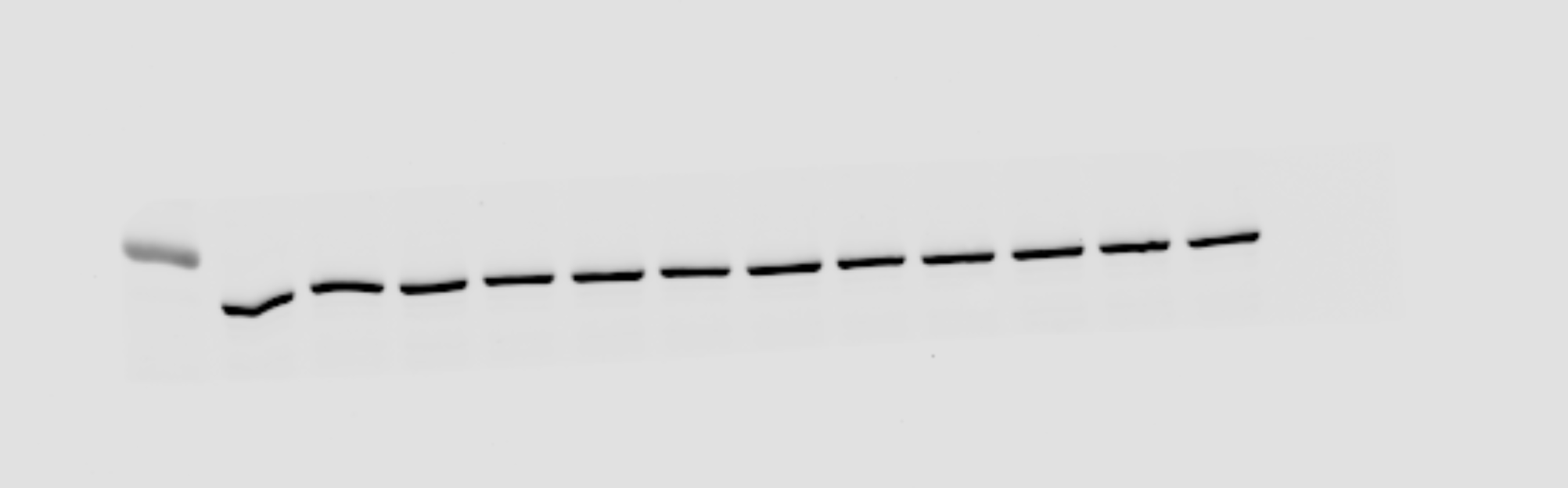

Supplement: Figure 2—source data 1. [file elife-82861-fig2-data1.zip › Zap70-total.tif.tif]

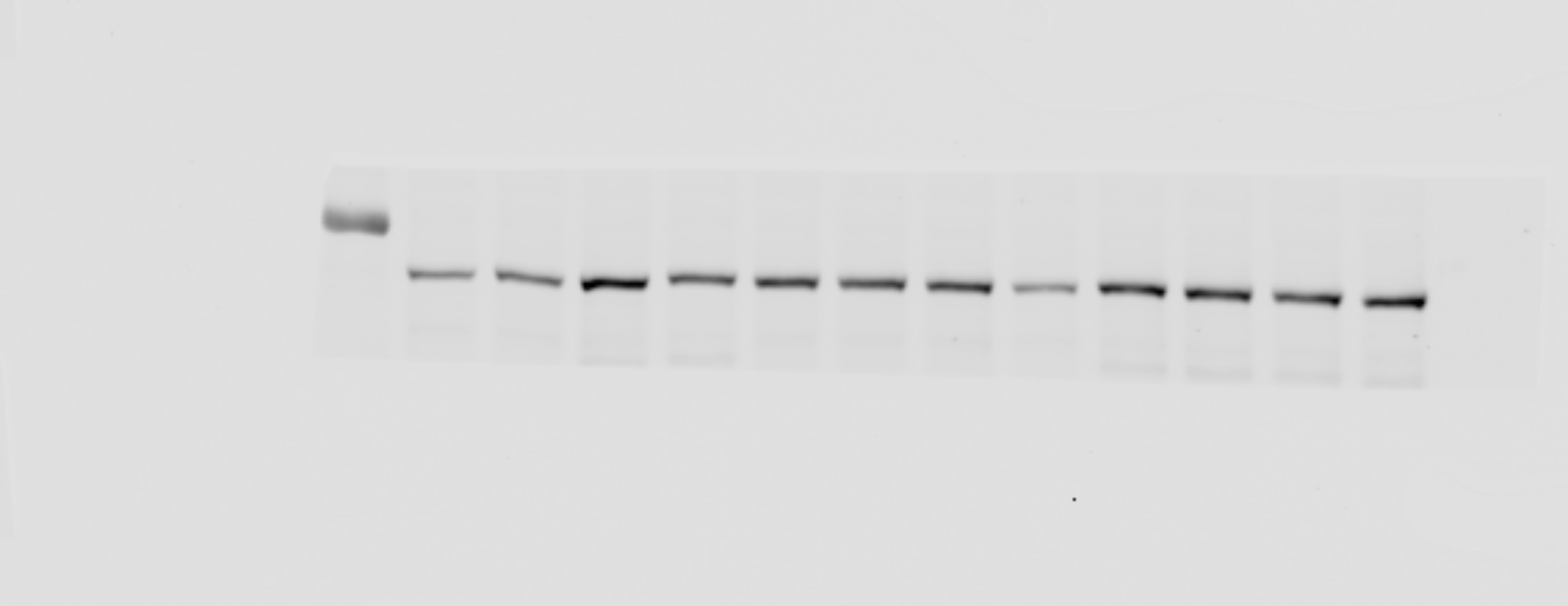

Supplement: Figure 2—source data 1. [file elife-82861-fig2-data1.zip › Zap70Y319.tif.tif]

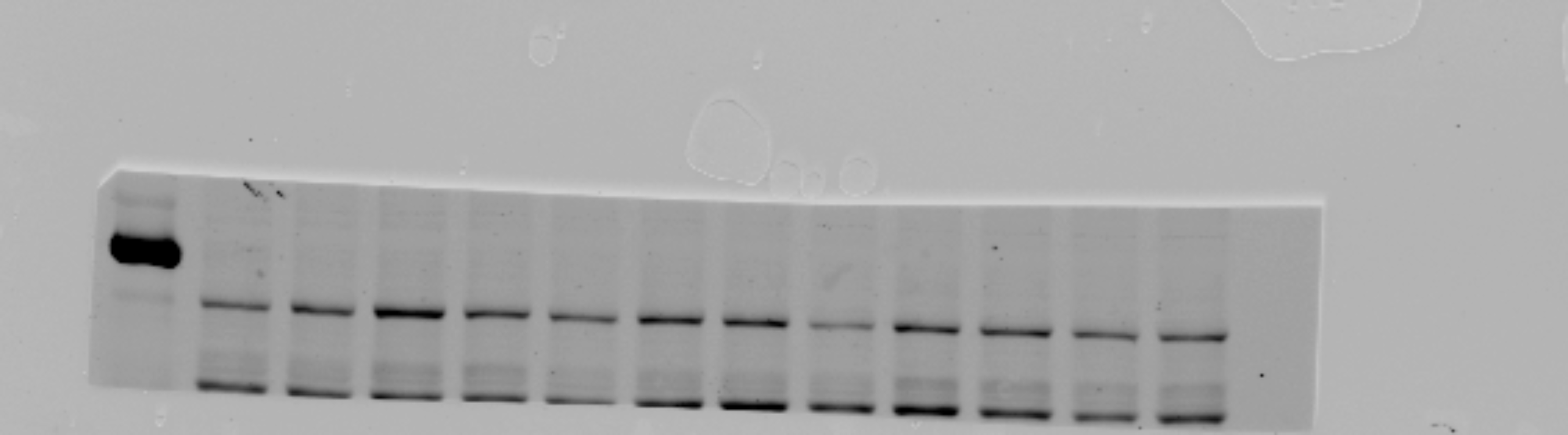

Supplement: Figure 2—source data 1. [file elife-82861-fig2-data1.zip › Zap70Y493.tif.tif]

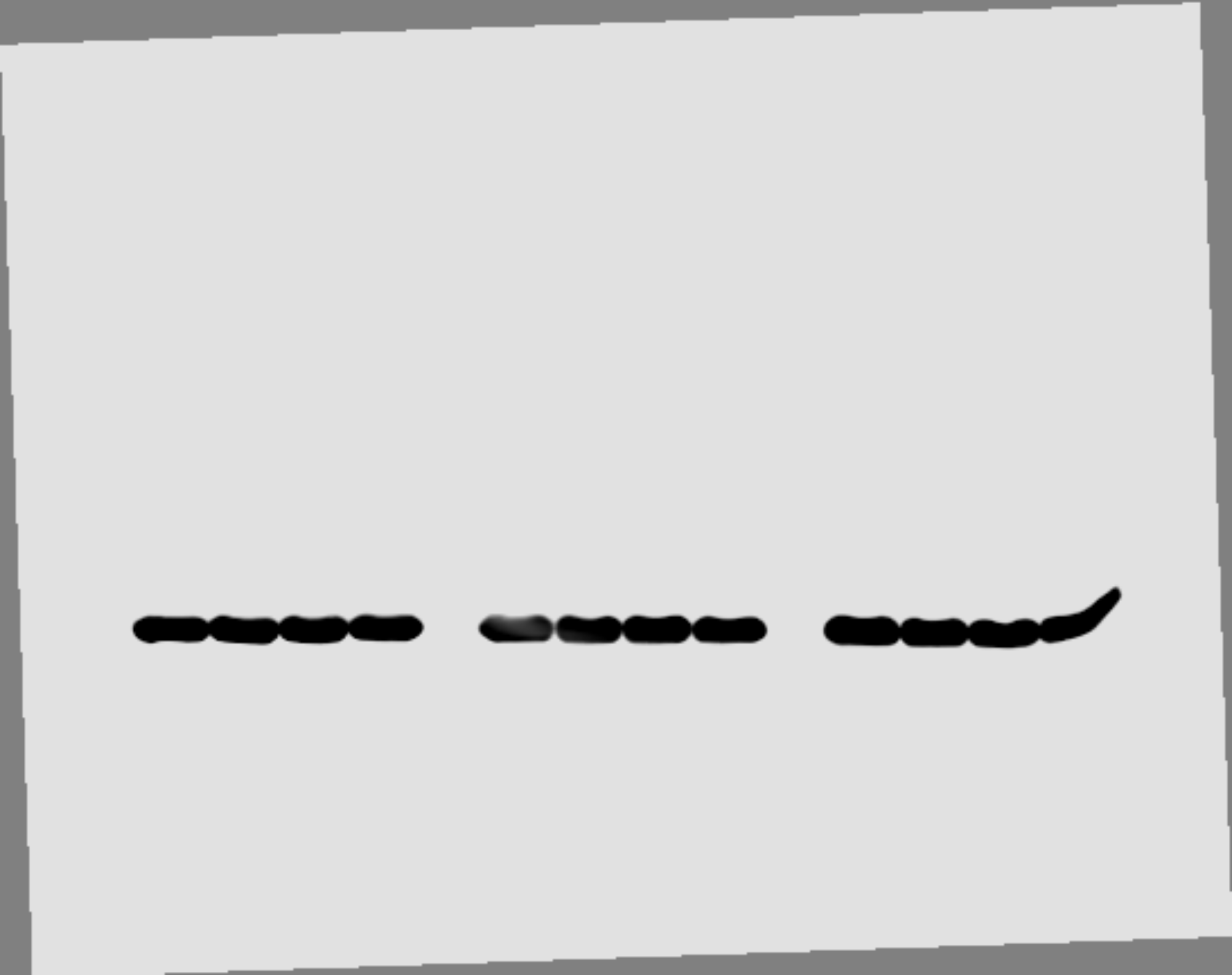

Supplement: Figure 2—figure supplement 2—source data 1. [file elife-82861-fig2-figsupp2-data1.zip › beta-actin.tif.tif]

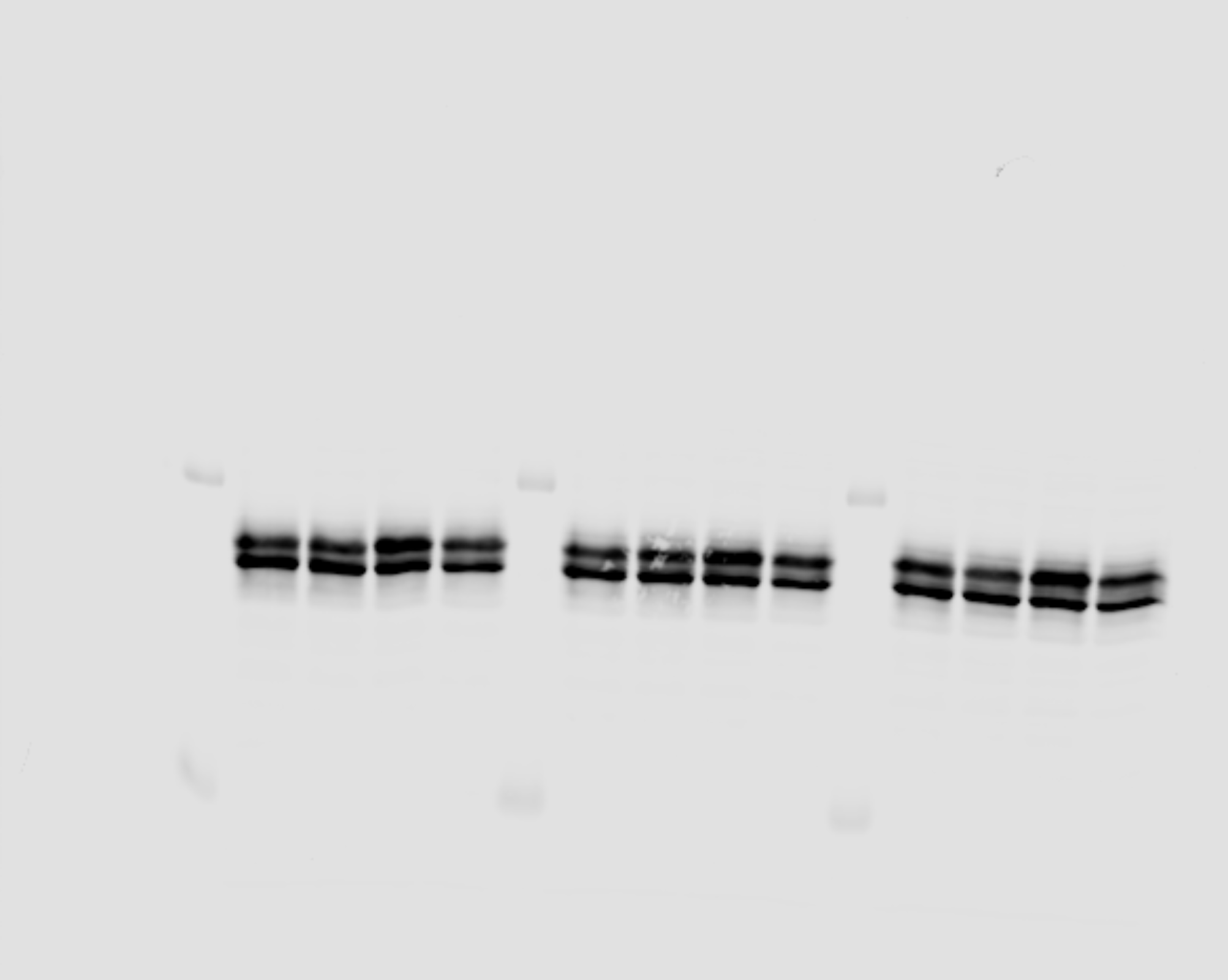

Supplement: Figure 2—figure supplement 2—source data 1. [file elife-82861-fig2-figsupp2-data1.zip › Lcktotal.tif]

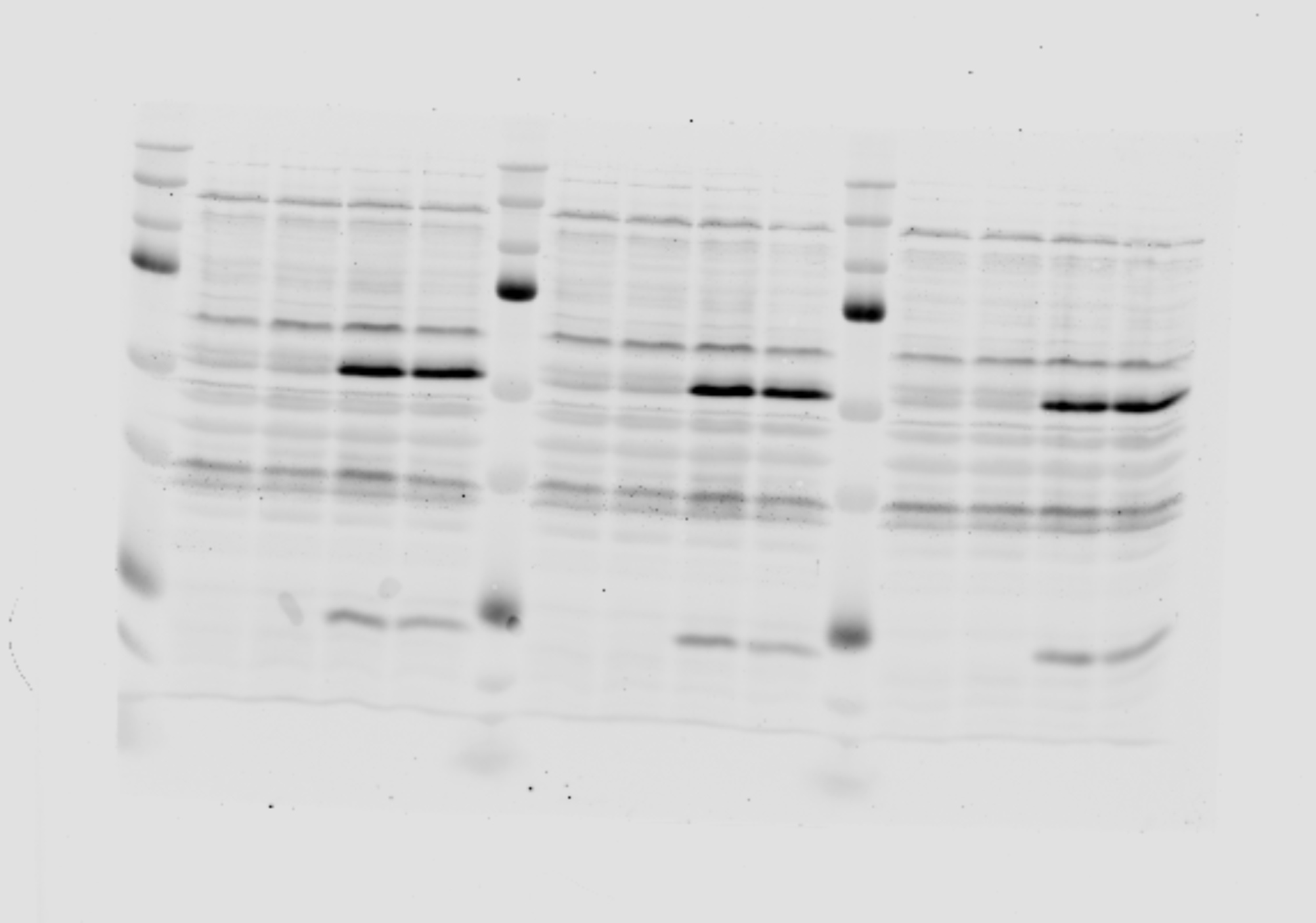

Supplement: Figure 2—figure supplement 2—source data 1. [file elife-82861-fig2-figsupp2-data1.zip › LckY394.tif.tif]

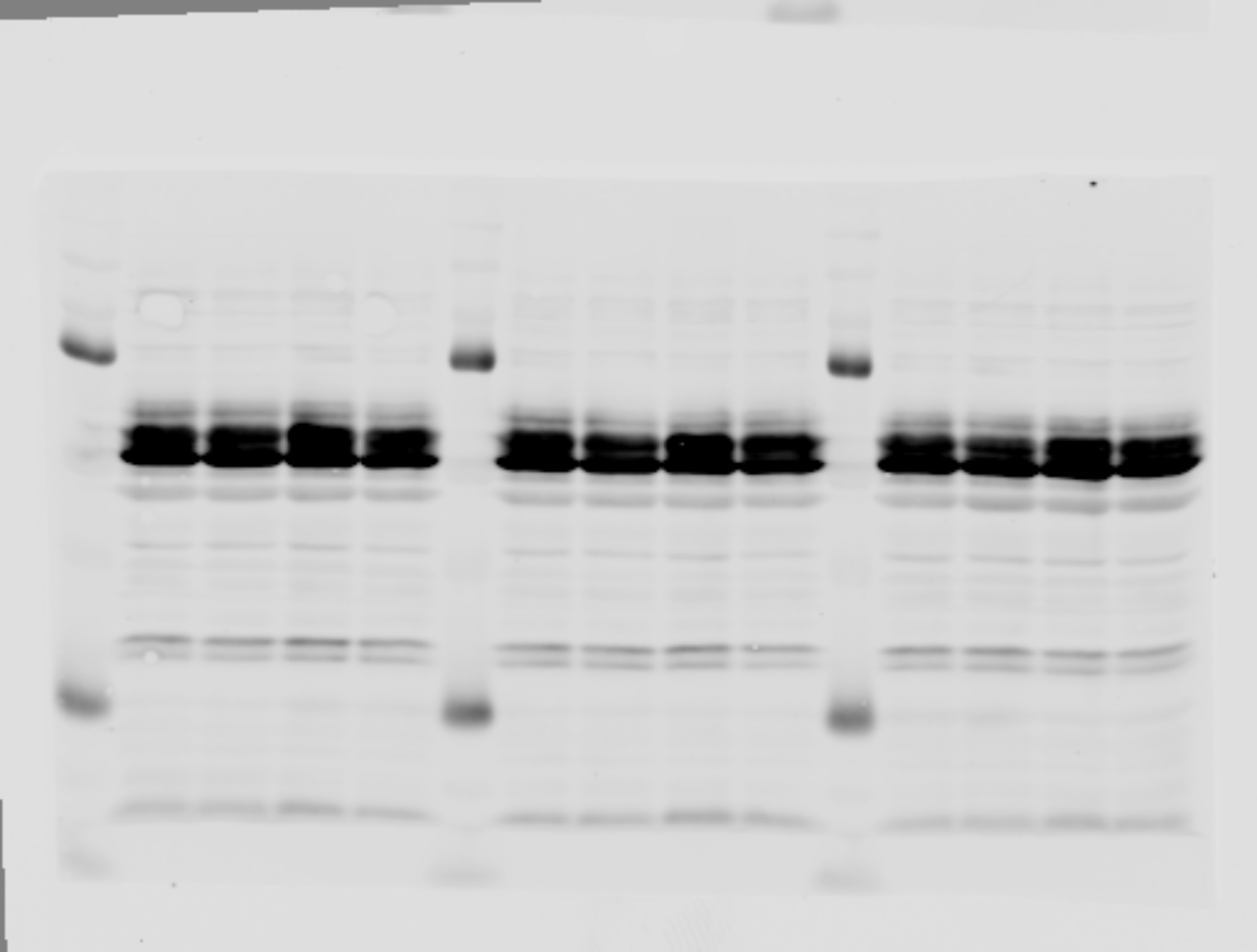

Supplement: Figure 2—figure supplement 2—source data 1. [file elife-82861-fig2-figsupp2-data1.zip › LckY505.tif.tif]

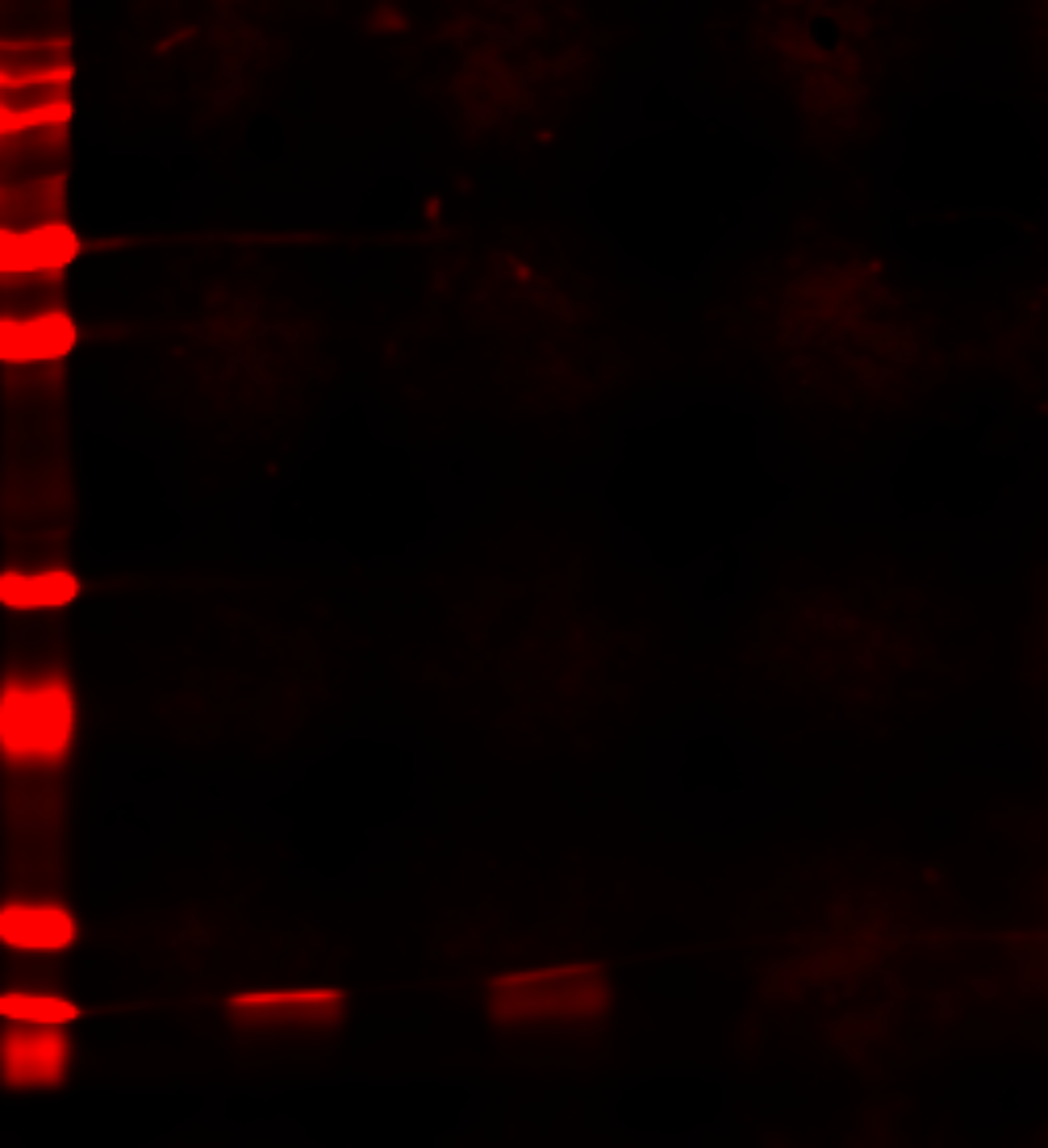

Supplement: Figure 7—source data 1. [file elife-82861-fig7-data1.zip › 02062022co_IPPITCR680.tif]

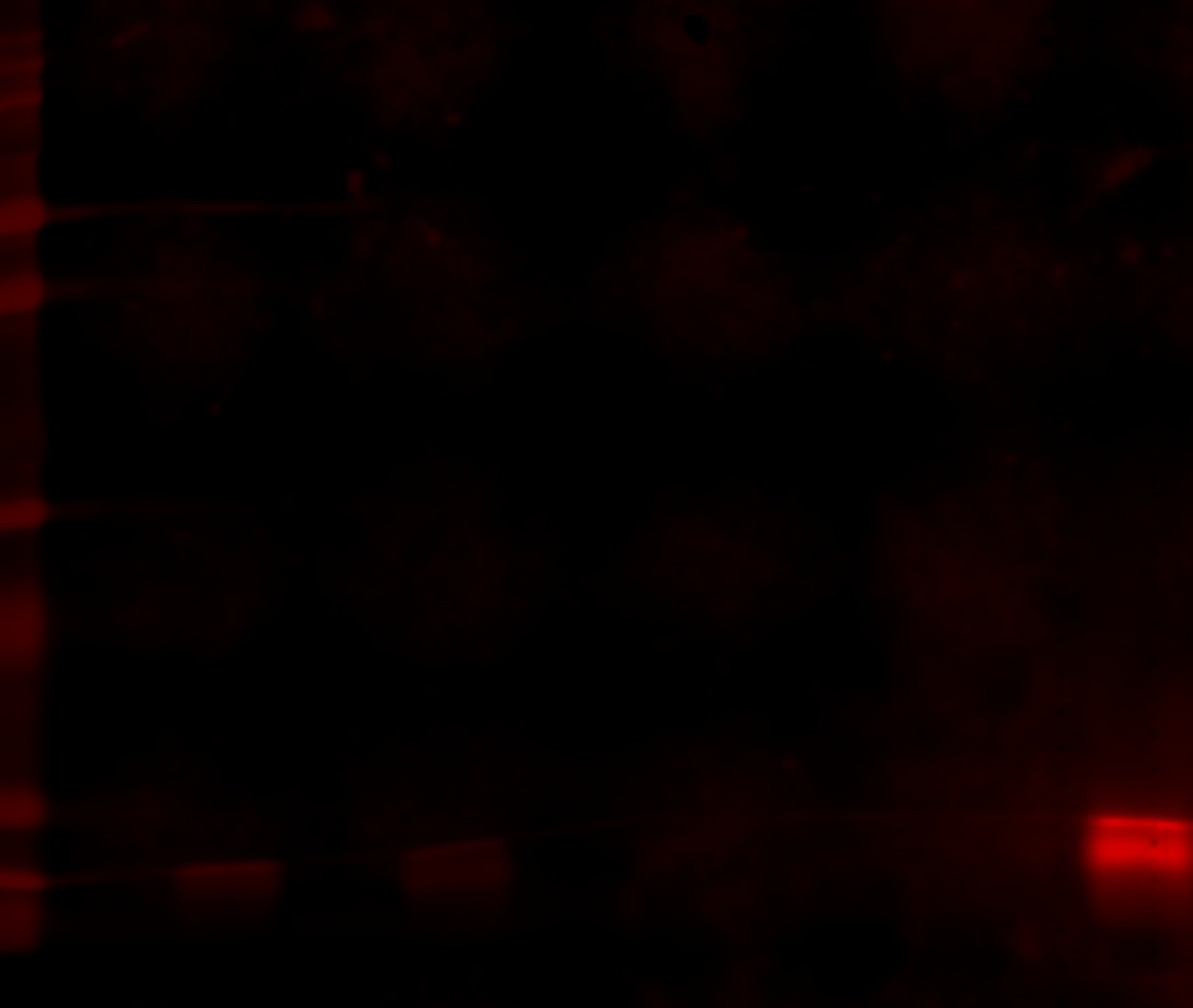

Supplement: Figure 7—source data 1. [file elife-82861-fig7-data1.zip › 02062022co_IPPITCR680_positivectrl_4.tif]

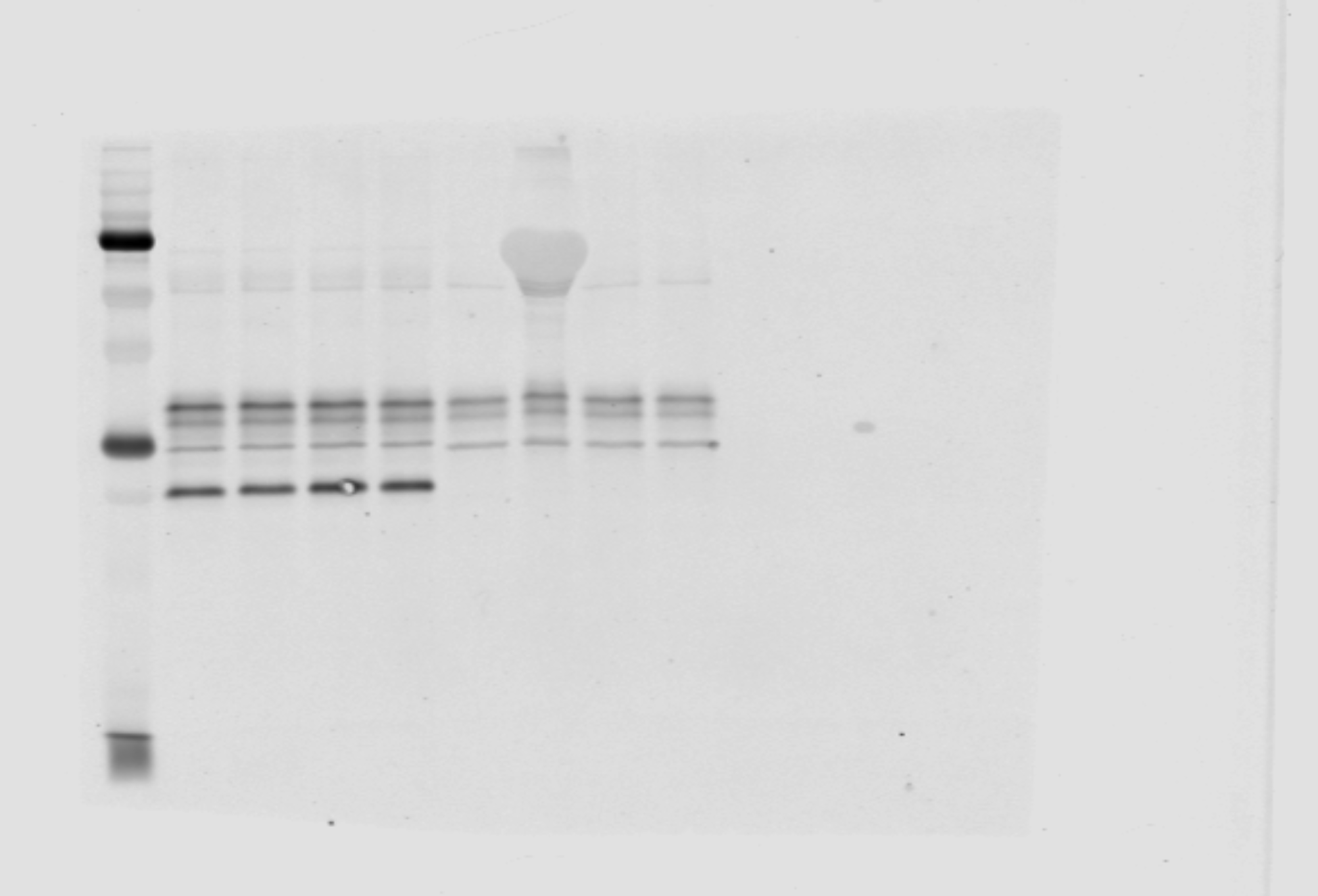

Supplement: Figure 7—source data 1. [file elife-82861-fig7-data1.zip › IP-CD3epsilon.tif.tif]

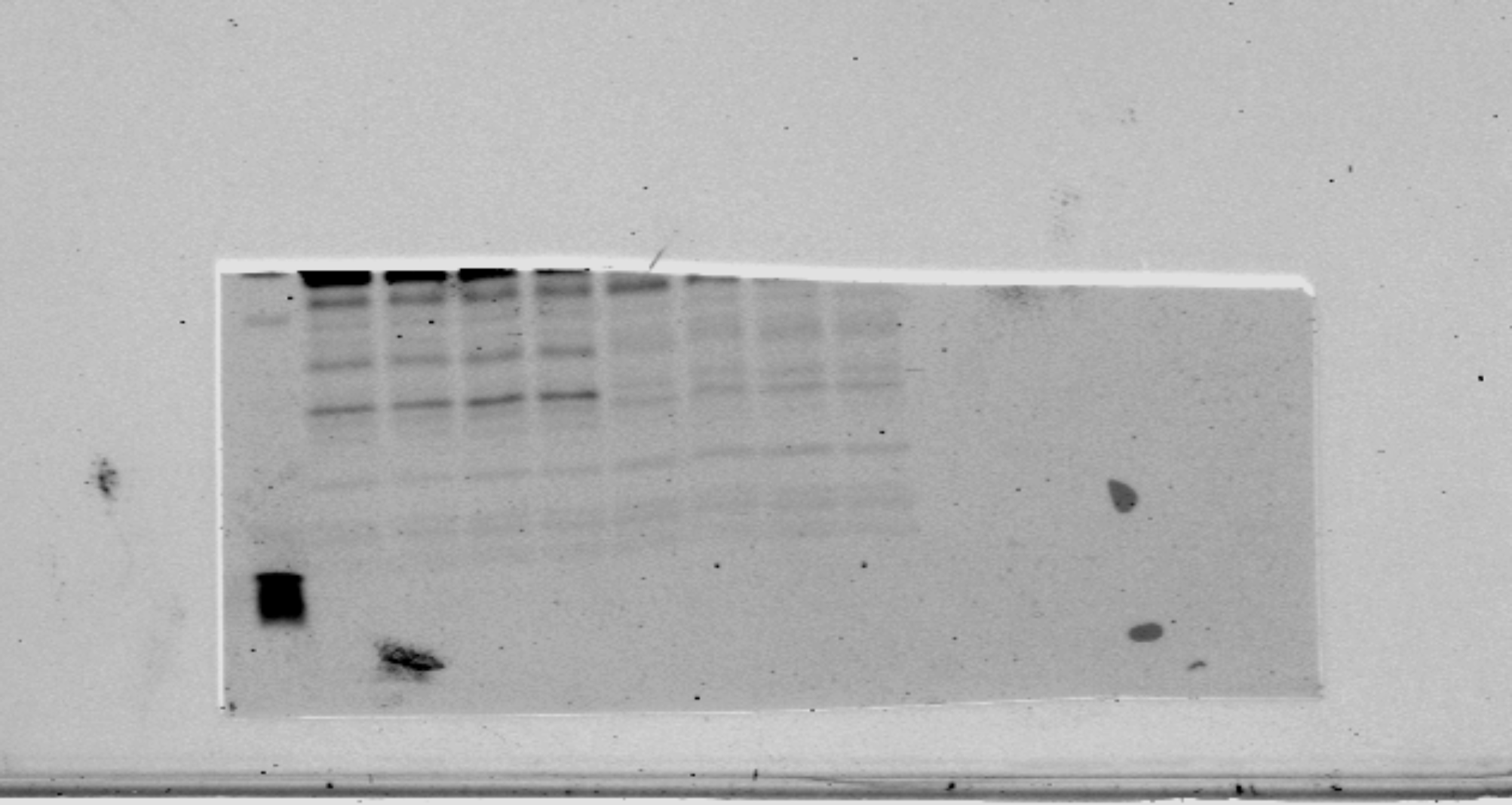

Supplement: Figure 7—source data 1. [file elife-82861-fig7-data1.zip › IP-CD3zeta.tif.tif]

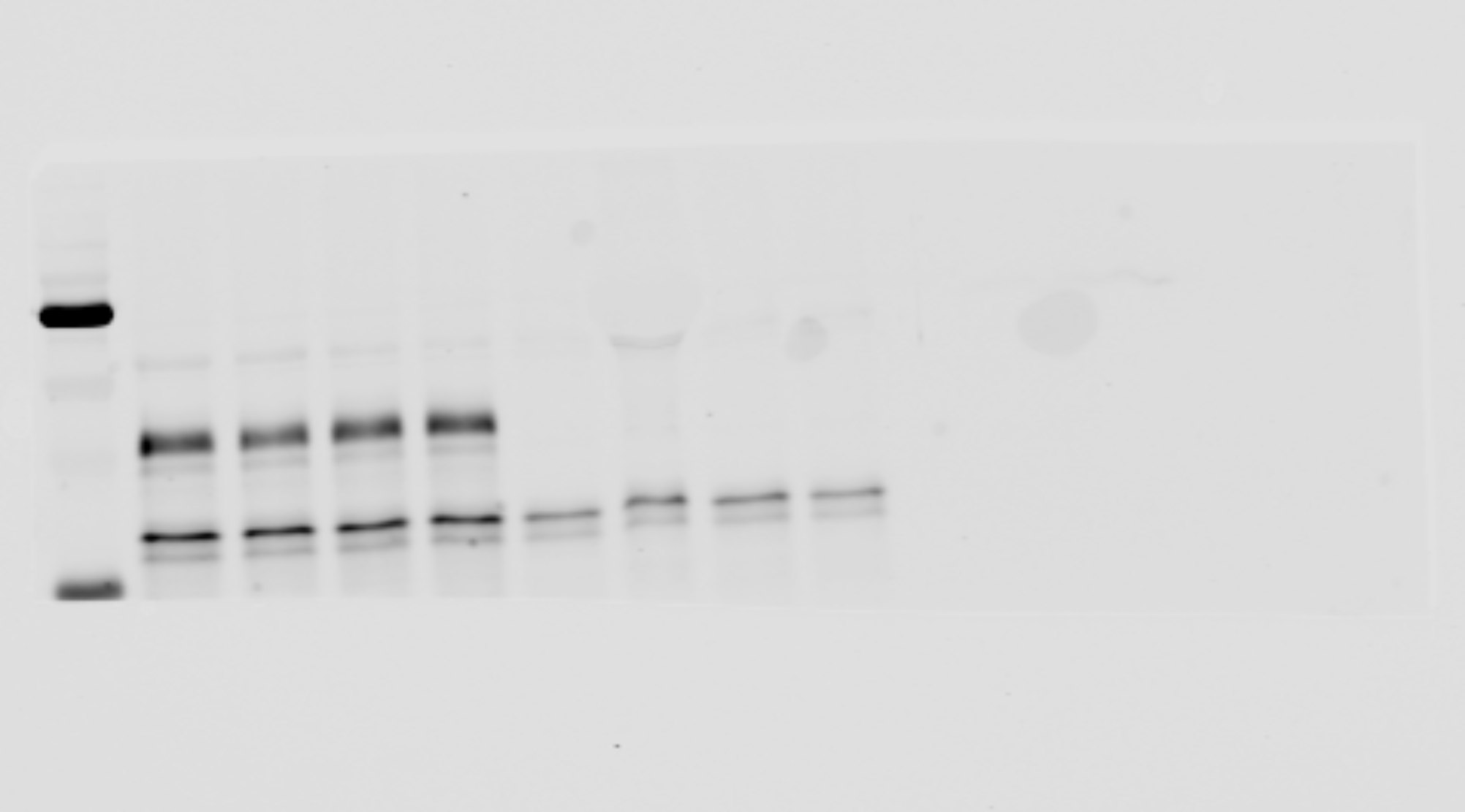

Supplement: Figure 7—source data 1. [file elife-82861-fig7-data1.zip › IP-TCRbeta.tif.tif]

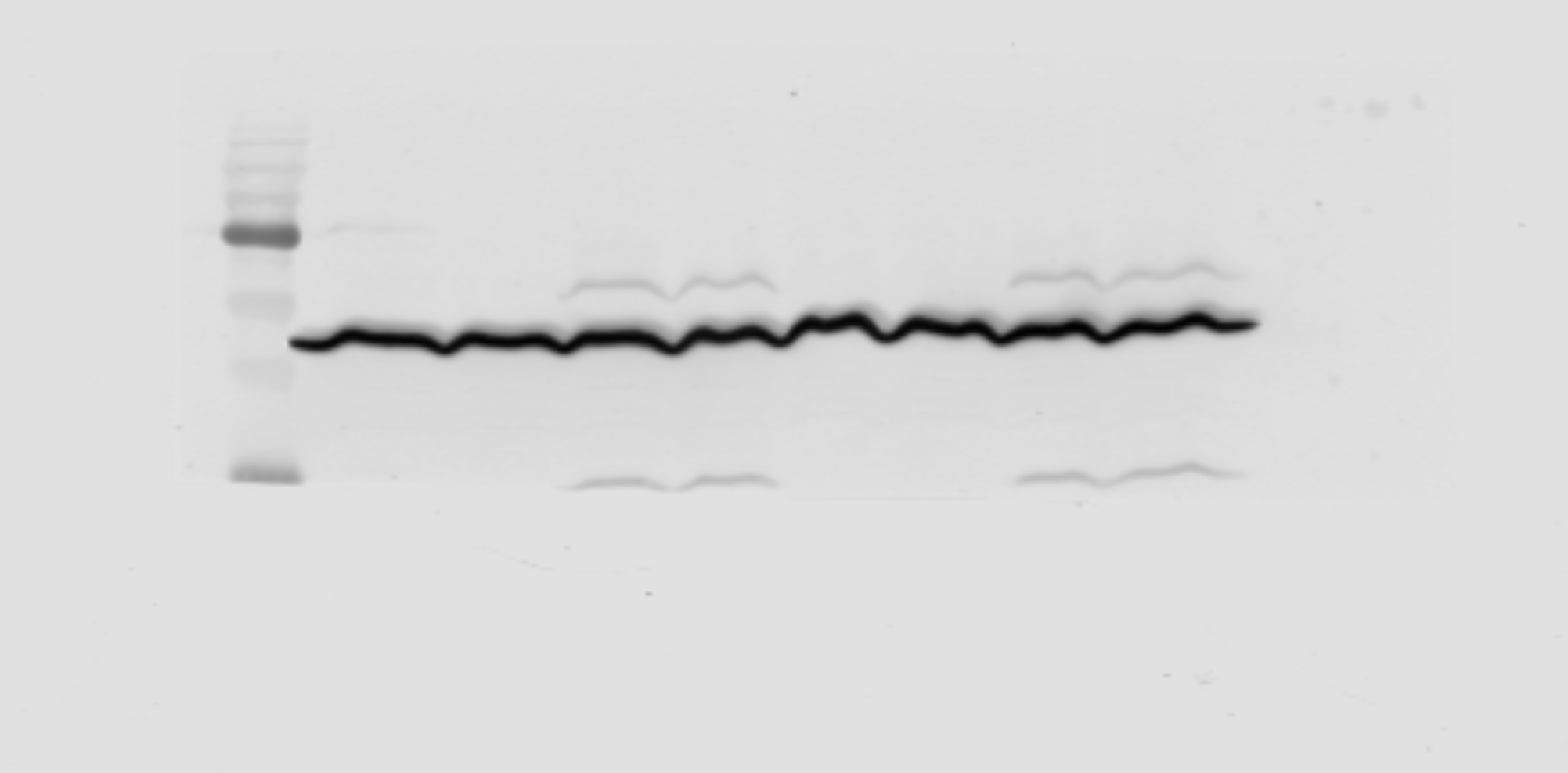

Supplement: Figure 7—source data 1. [file elife-82861-fig7-data1.zip › wholelysate-beta-actin.tif.tif]

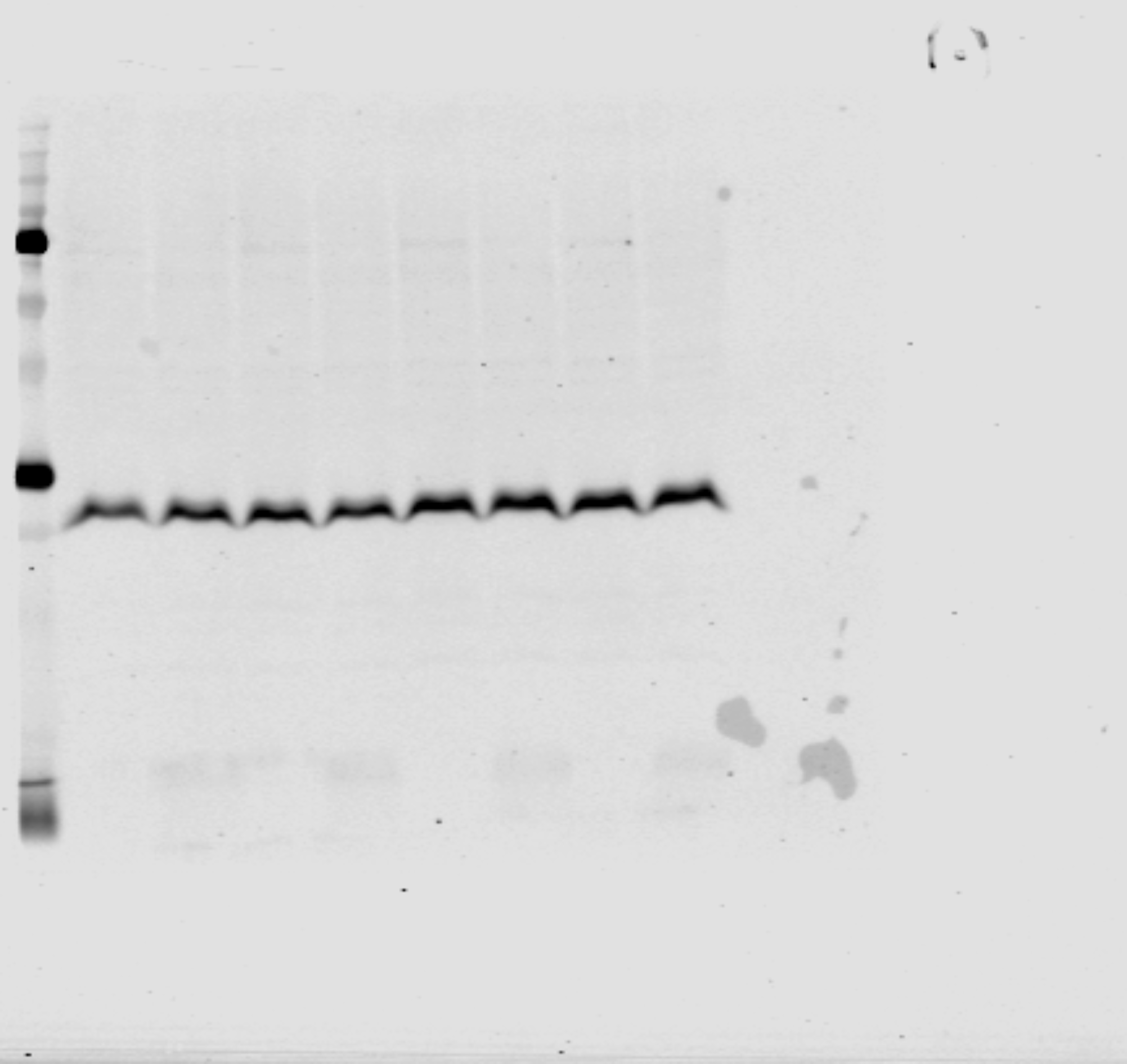

Supplement: Figure 7—source data 1. [file elife-82861-fig7-data1.zip › wholelysate-CD3epsilon.tif.tif]

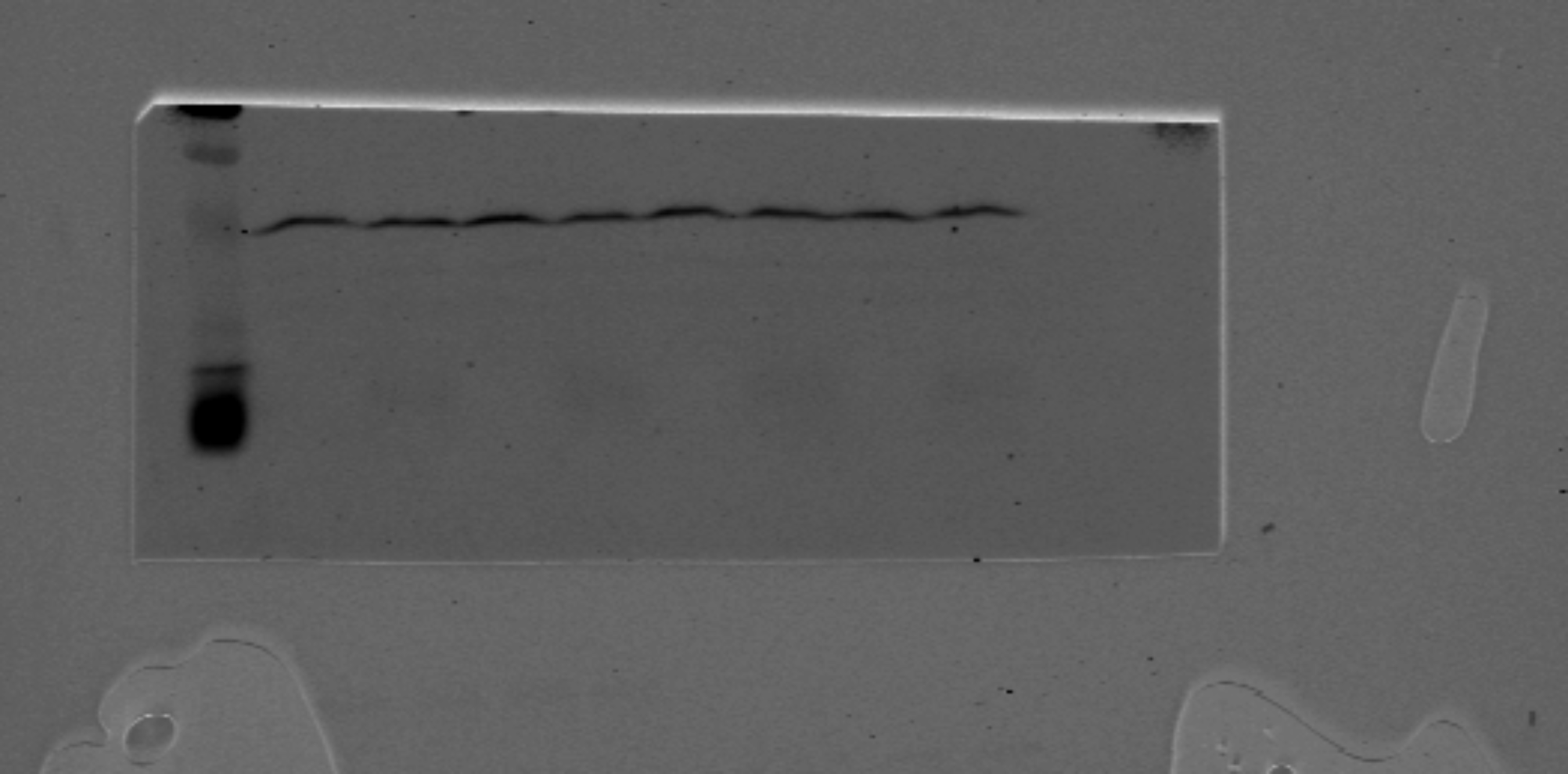

Supplement: Figure 7—source data 1. [file elife-82861-fig7-data1.zip › wholelysate-CD3zeta.tif.tif]

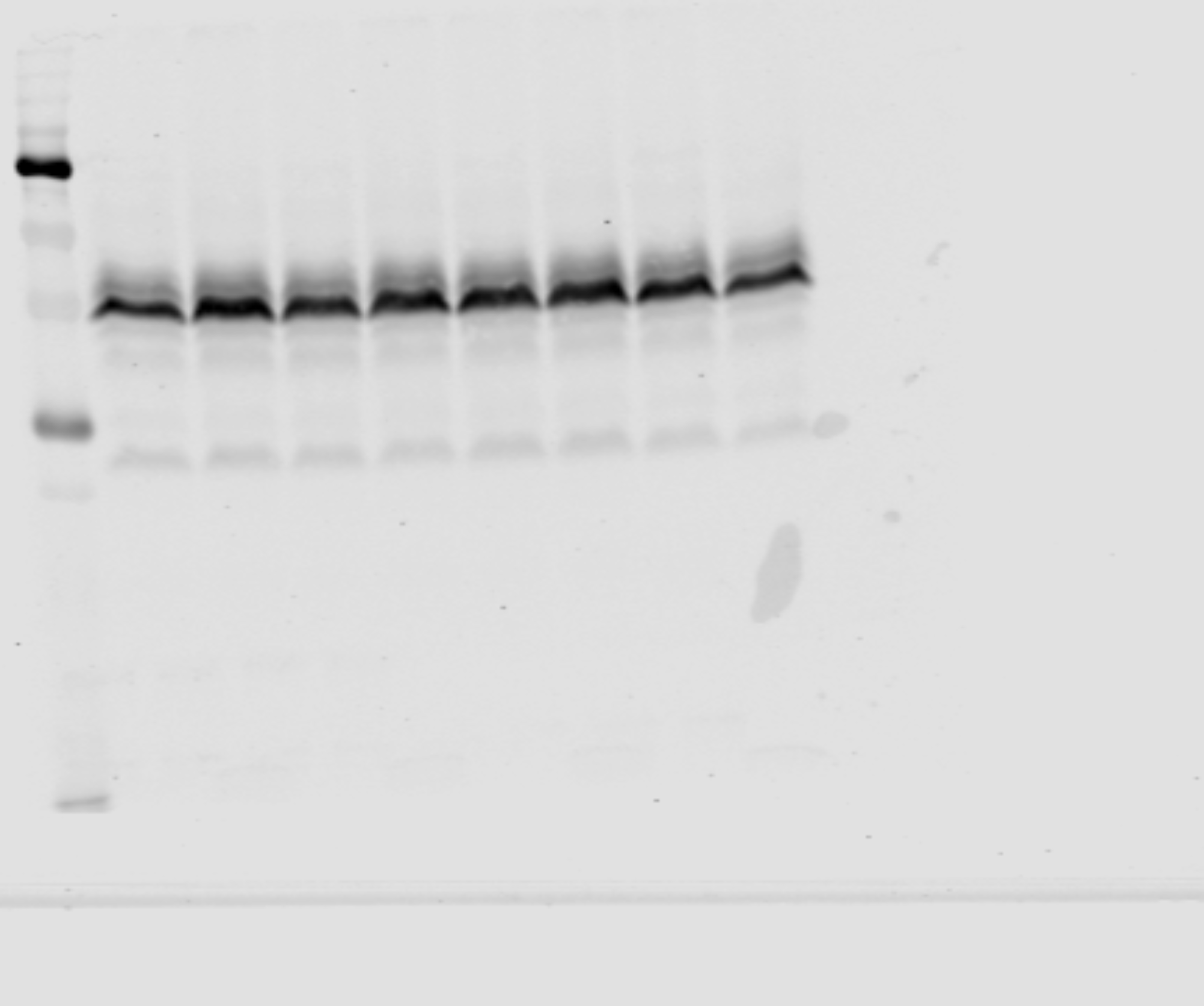

Supplement: Figure 7—source data 1. [file elife-82861-fig7-data1.zip › wholelysate-TCRbeta.tif.tif]

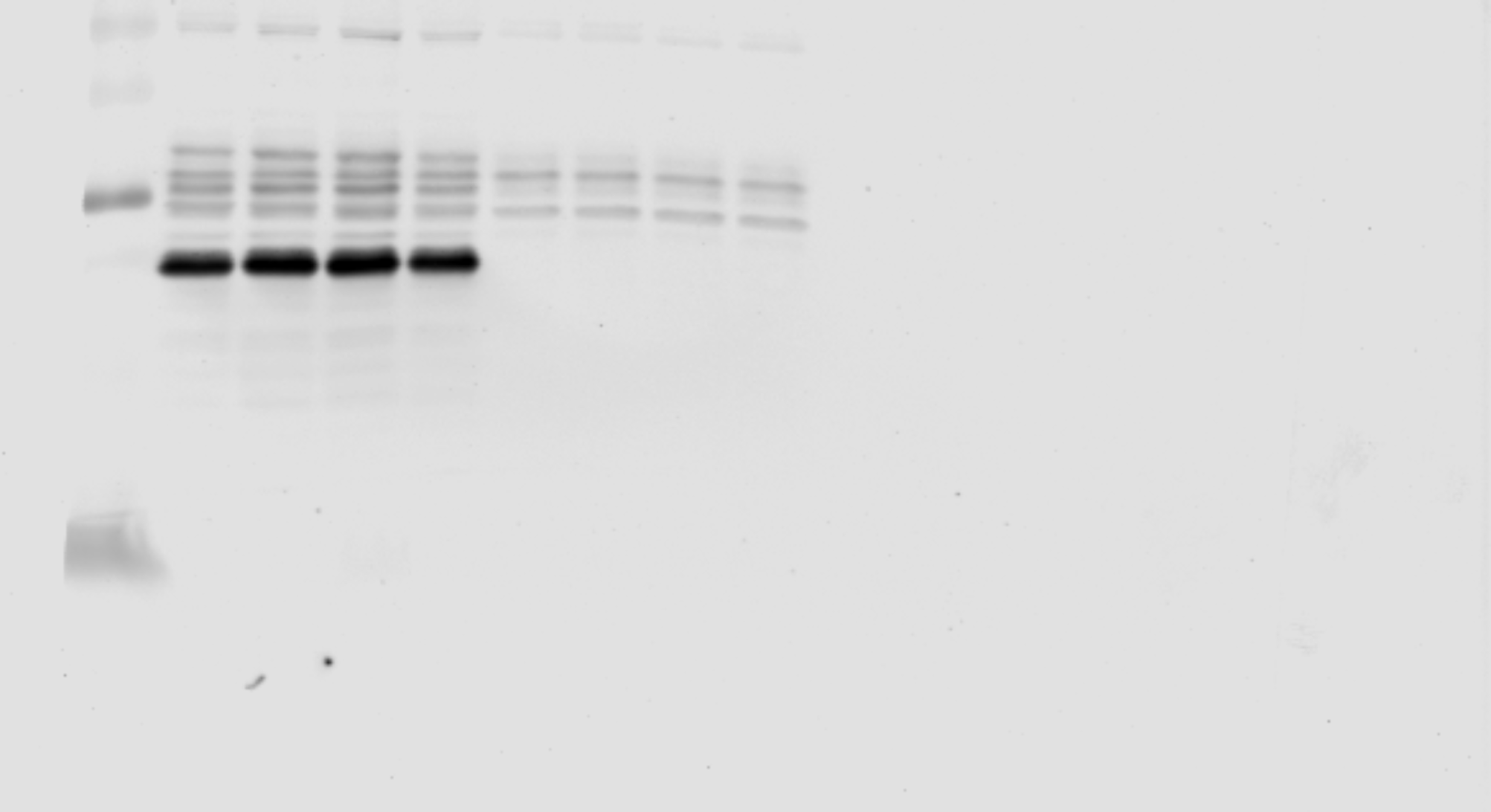

Supplement: Figure 8—source data 1. [file elife-82861-fig8-data1.zip › IP-CD3epsilon.tif.tif]

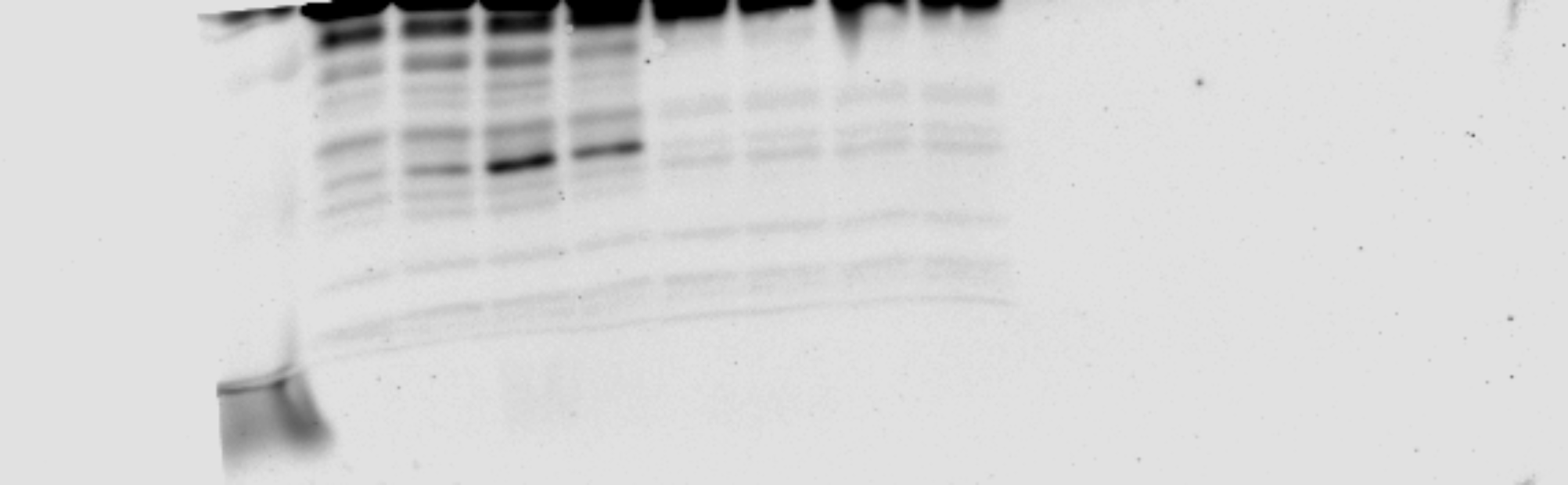

Supplement: Figure 8—source data 1. [file elife-82861-fig8-data1.zip › IP-CD3zeta.tif.tif]

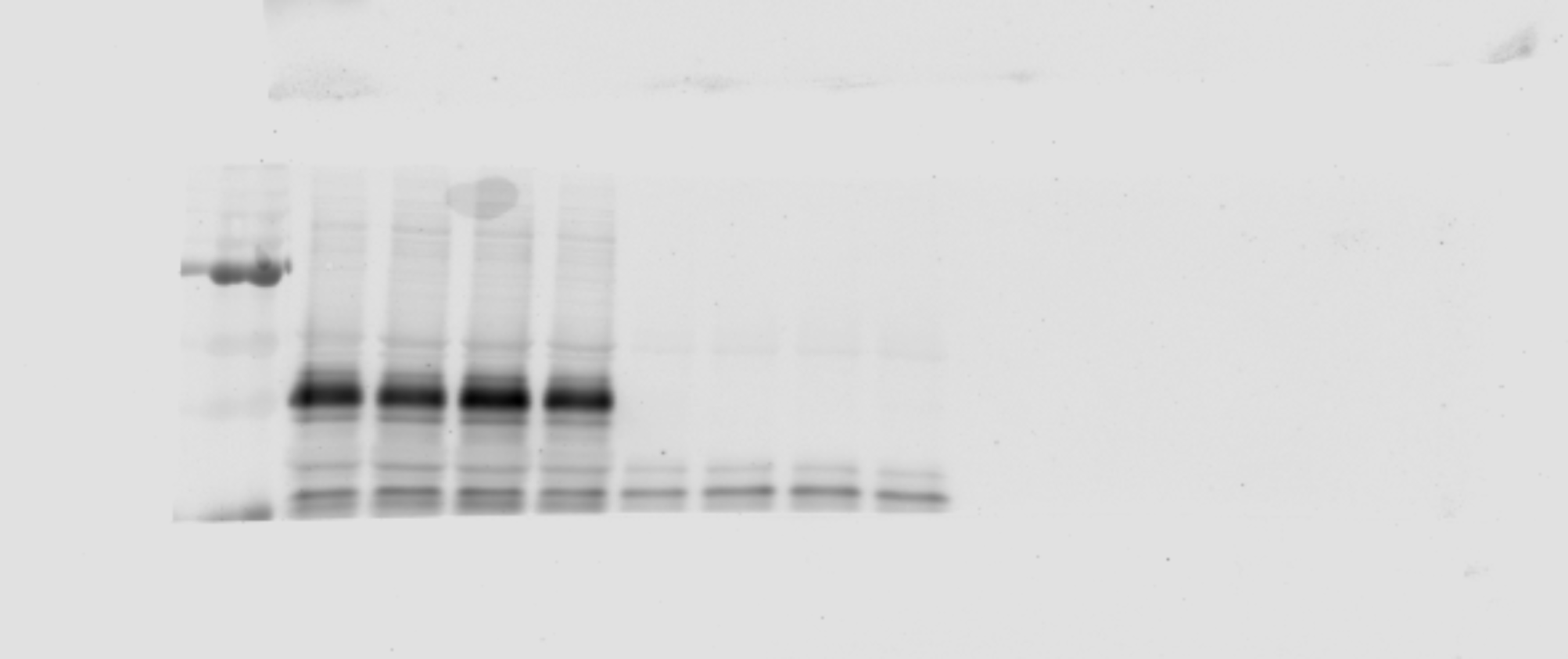

Supplement: Figure 8—source data 1. [file elife-82861-fig8-data1.zip › IP-TCRbeta.tif.tif]

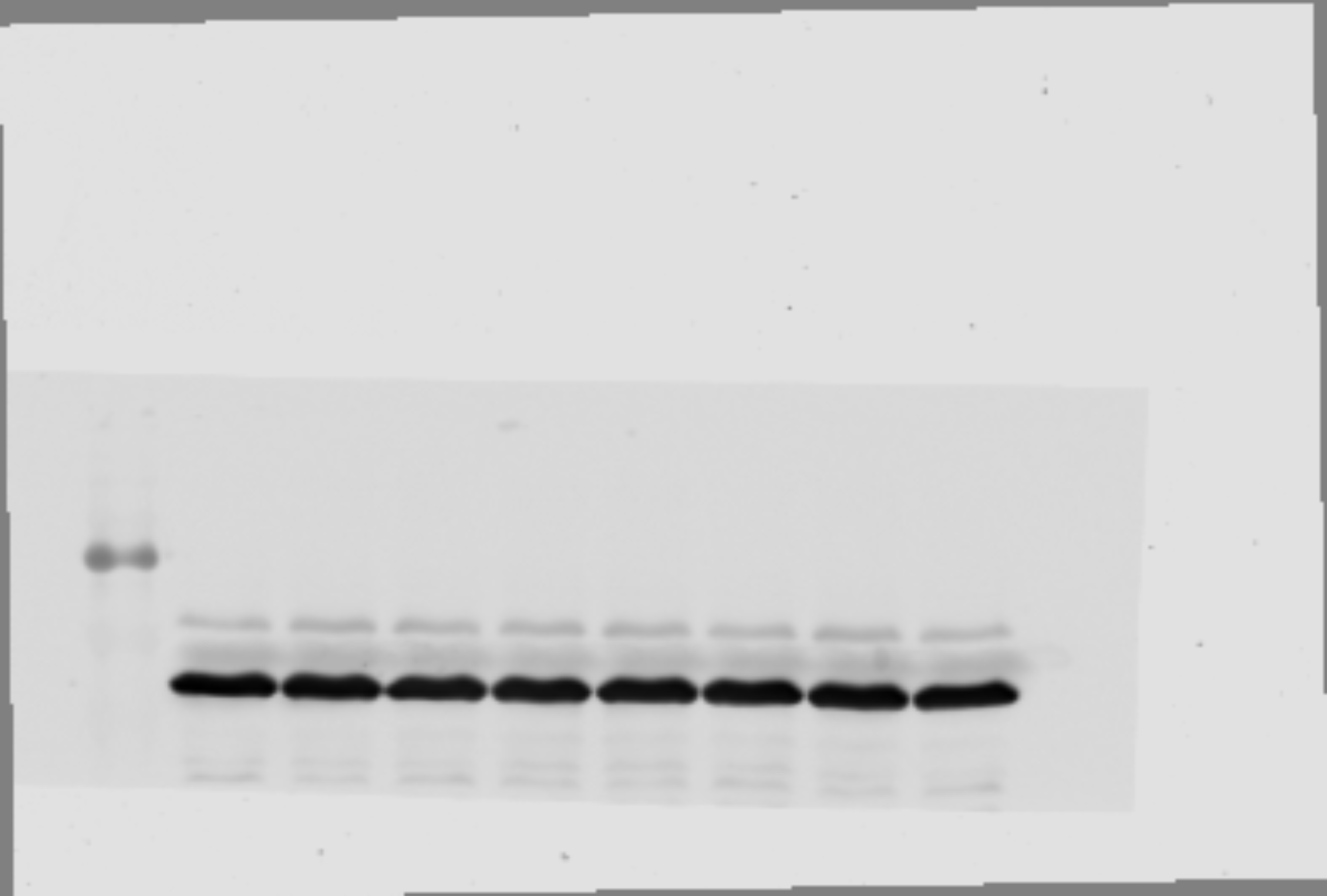

Supplement: Figure 8—source data 1. [file elife-82861-fig8-data1.zip › wholelysate-beta-actin.tif.tif]

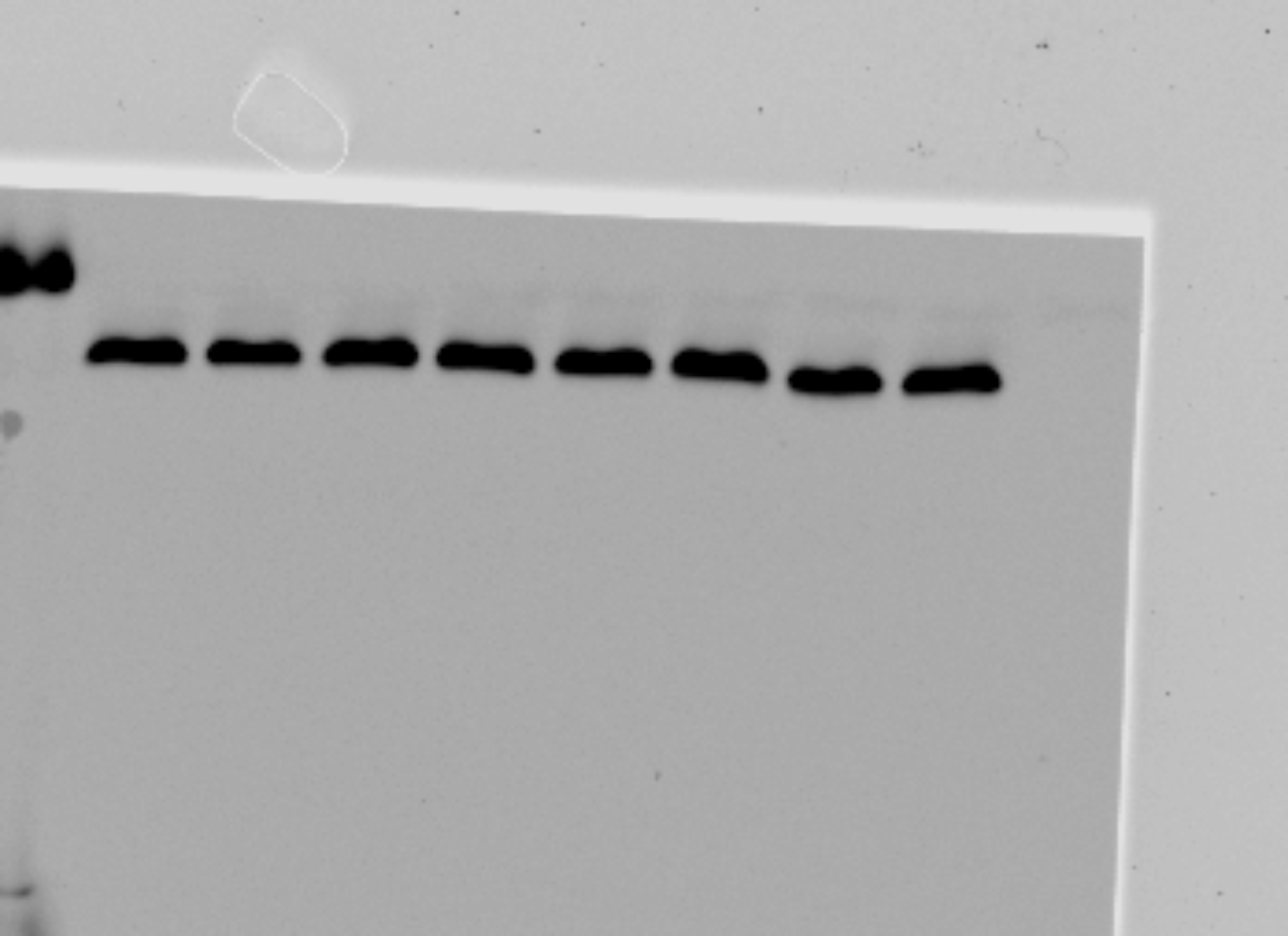

Supplement: Figure 8—source data 1. [file elife-82861-fig8-data1.zip › wholelysate-CD3epsilon.tif.tif]

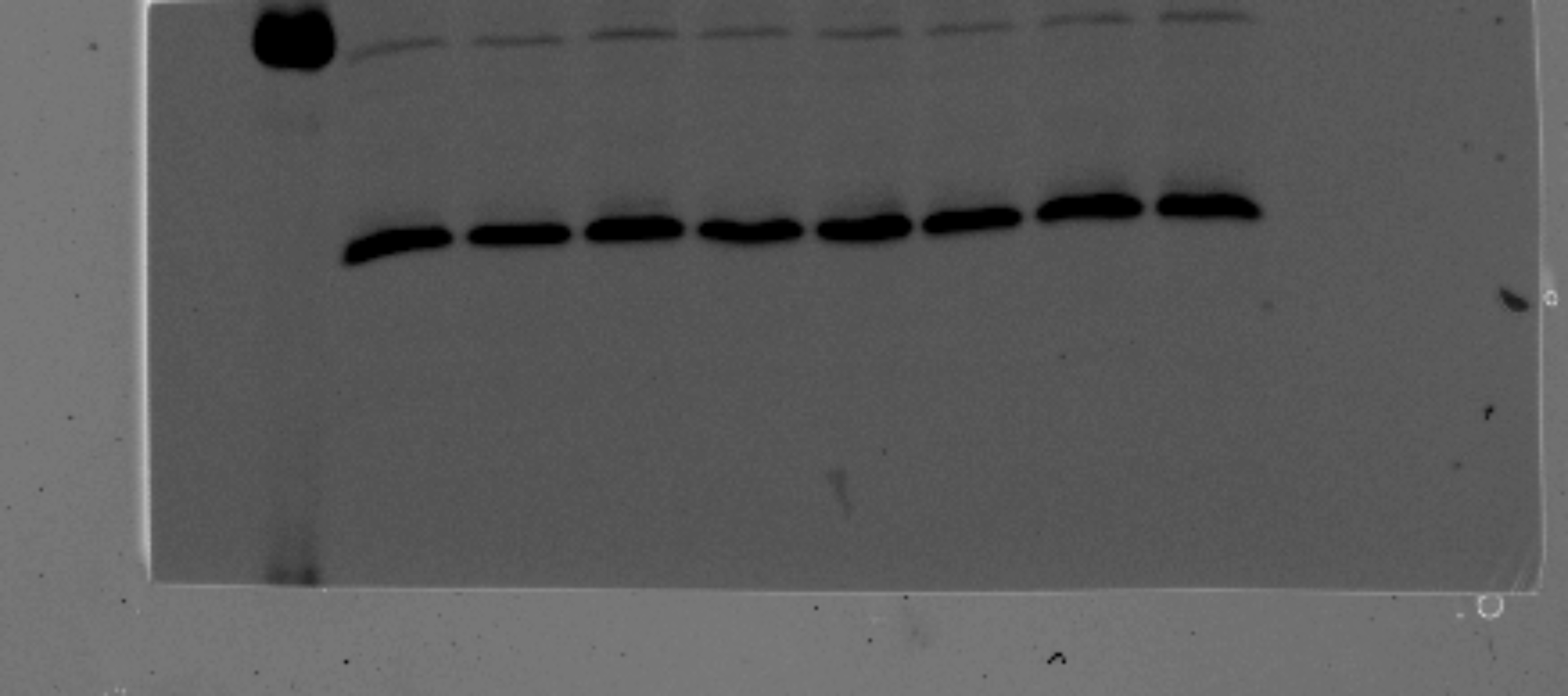

Supplement: Figure 8—source data 1. [file elife-82861-fig8-data1.zip › wholelysate-CD3z.tif.tif]

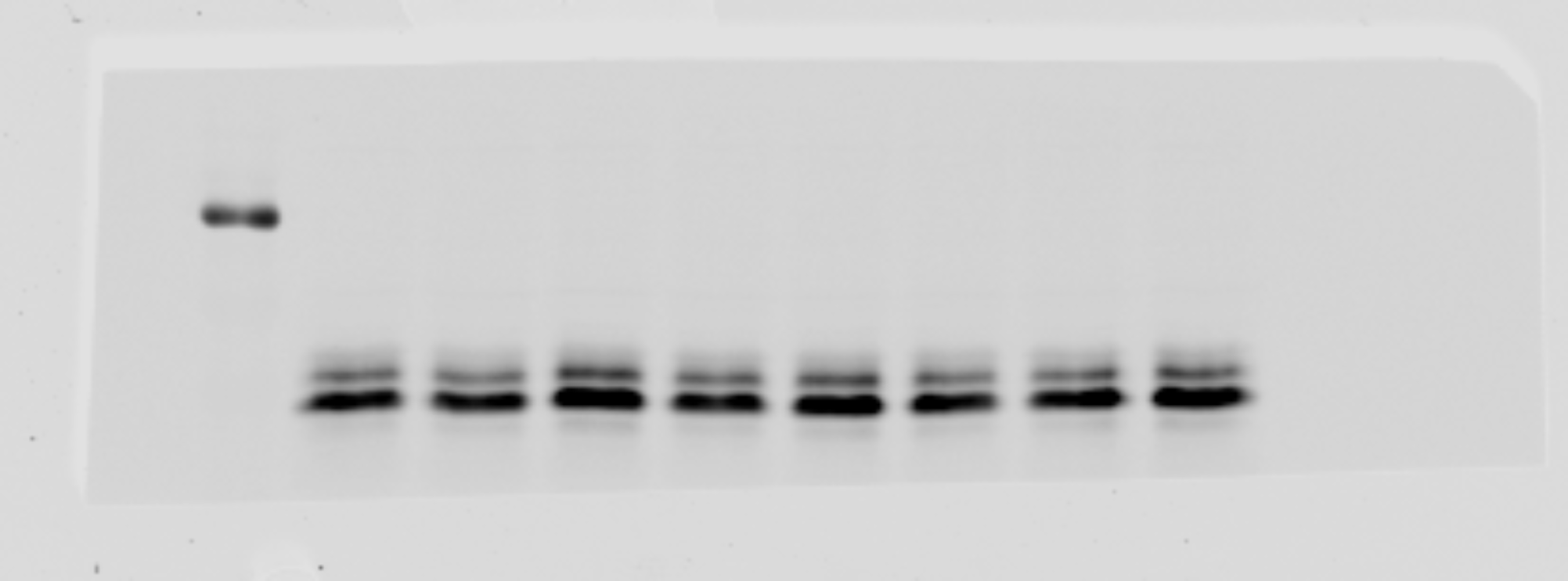

Supplement: Figure 8—source data 1. [file elife-82861-fig8-data1.zip › wholelysate-tcrbeta.tif.tif]
