# Supplementary material for: Allosteric inhibition of the T cell receptor by a designed membrane ligand
Source: eLife. 2023 Oct 5;12:e82861. doi: 10.7554/eLife.82861 (PMC10554751; doi:10.7554/eLife.82861)

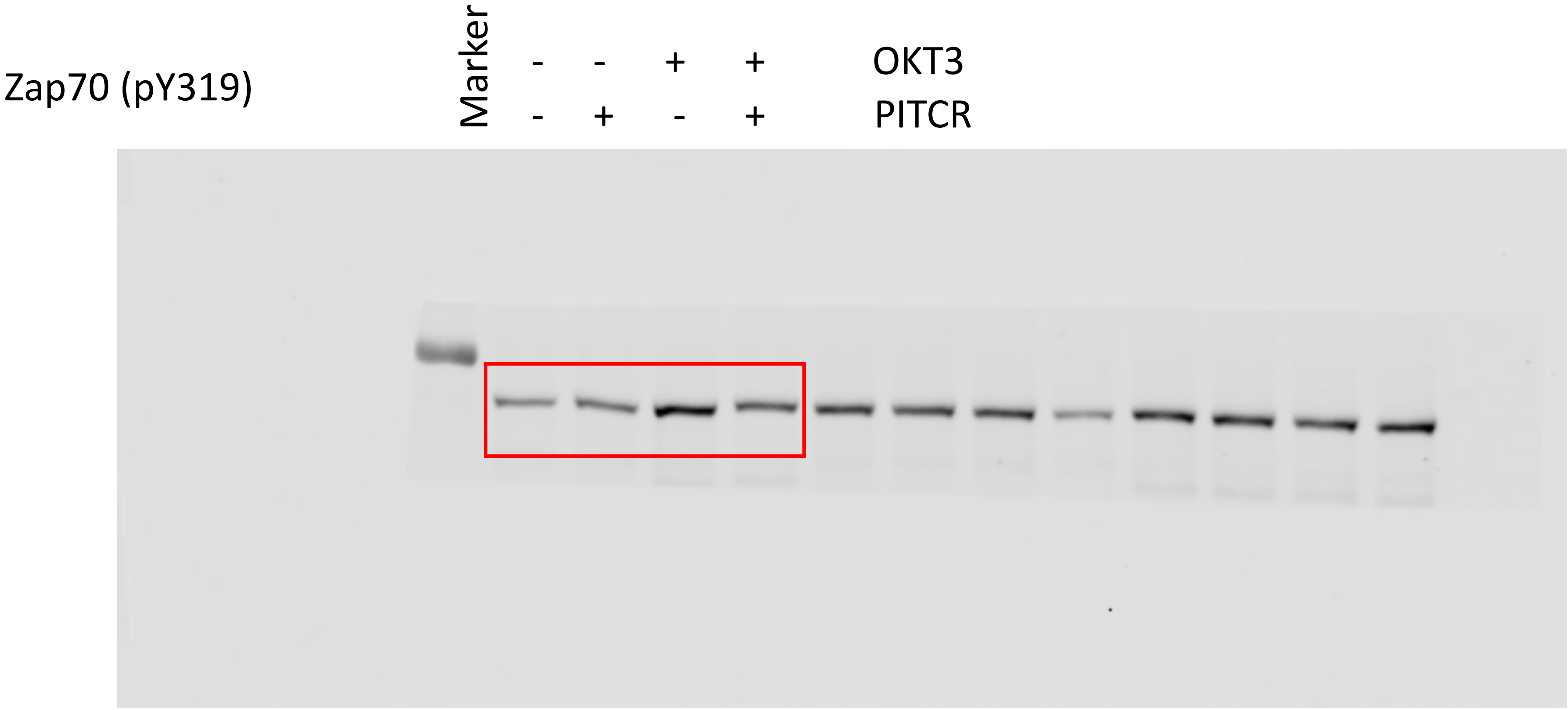

Zap70 (pY319)
Marker
- - + + OKT3
- + - + PITCR

Zap70 (pY493)

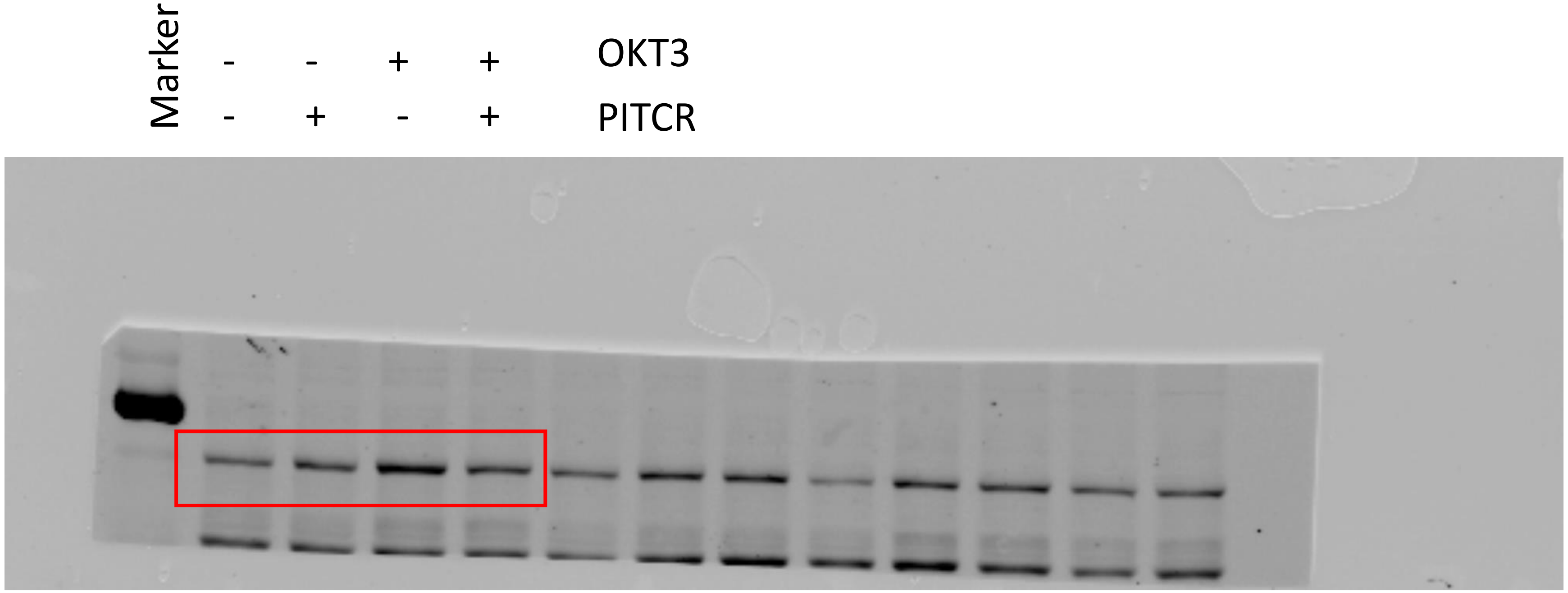

Zap70 (total)

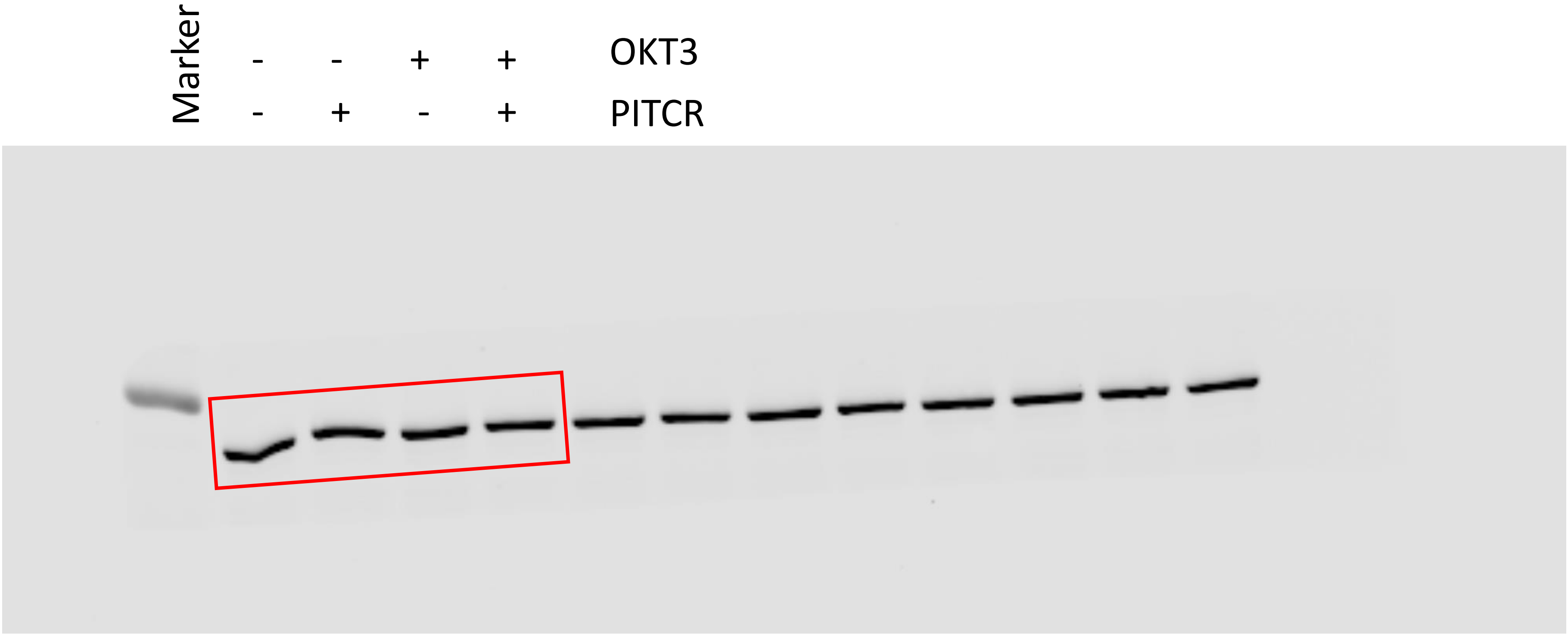

LAT (pY191)

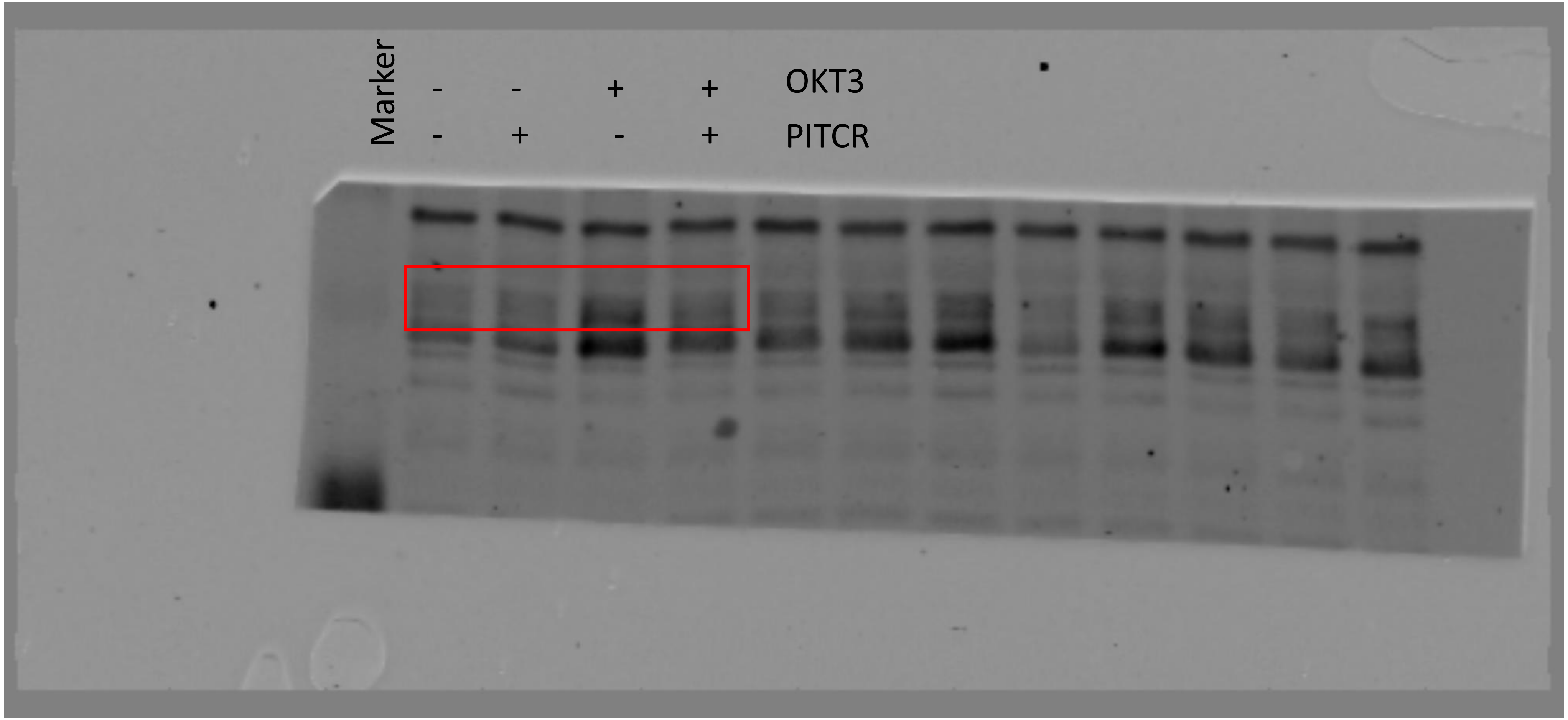

LAT (pY132)

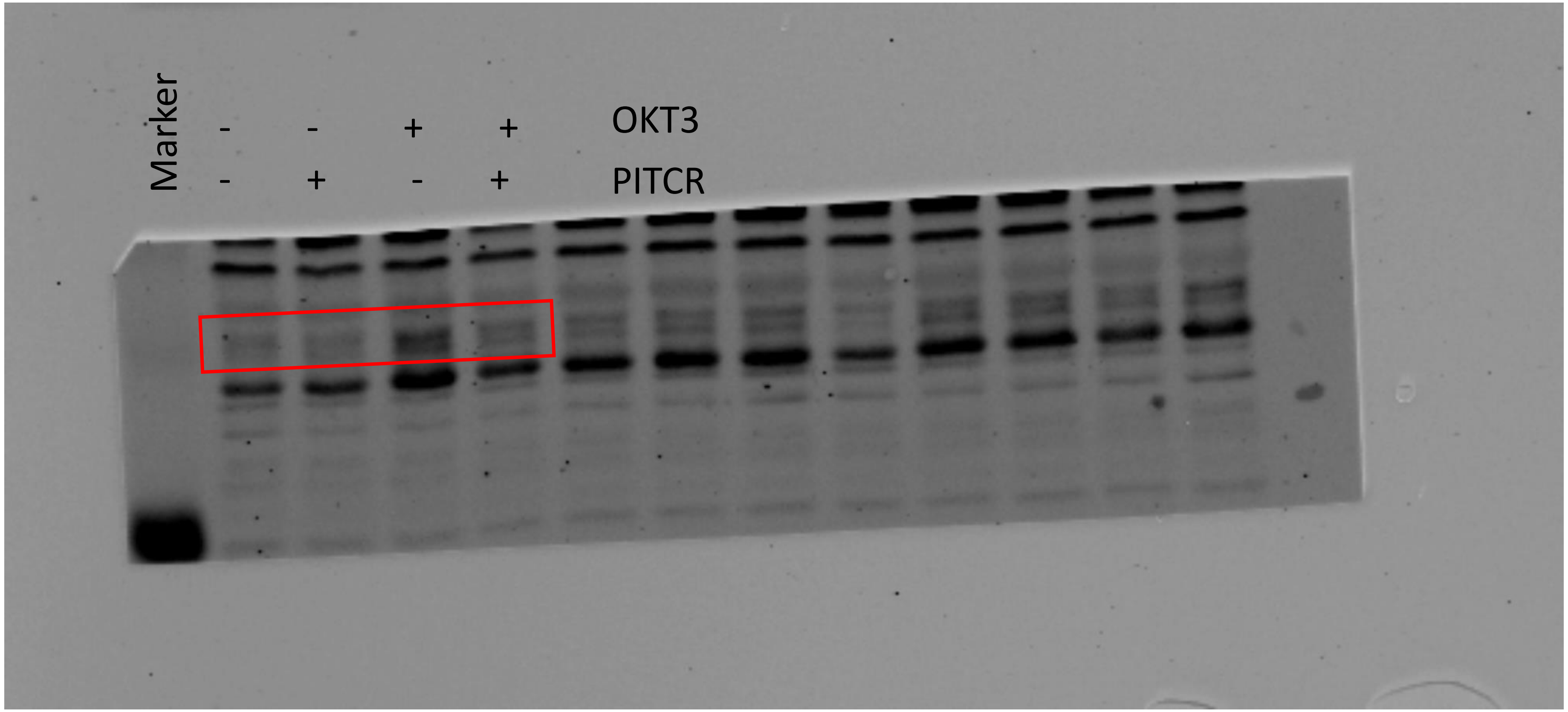

LAT (total)

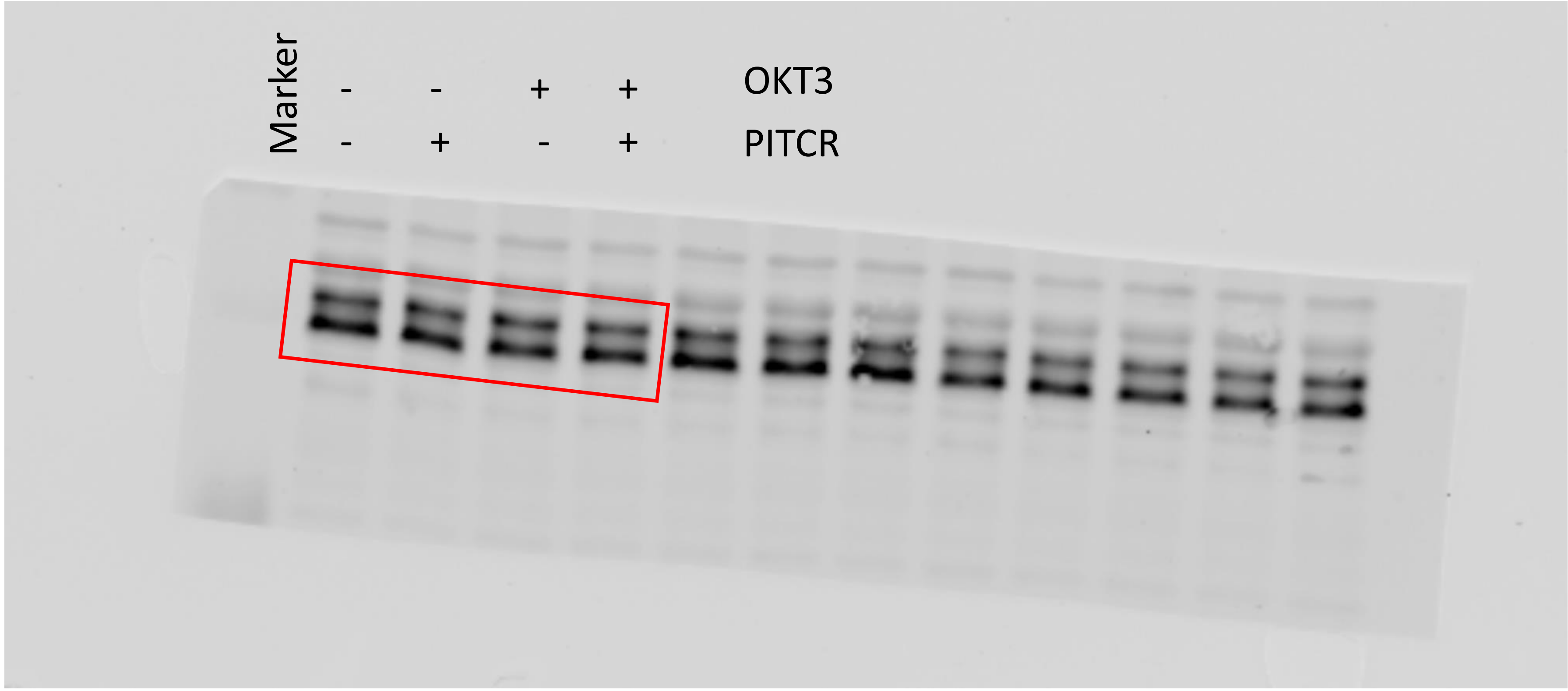

SLP76 (pY128)

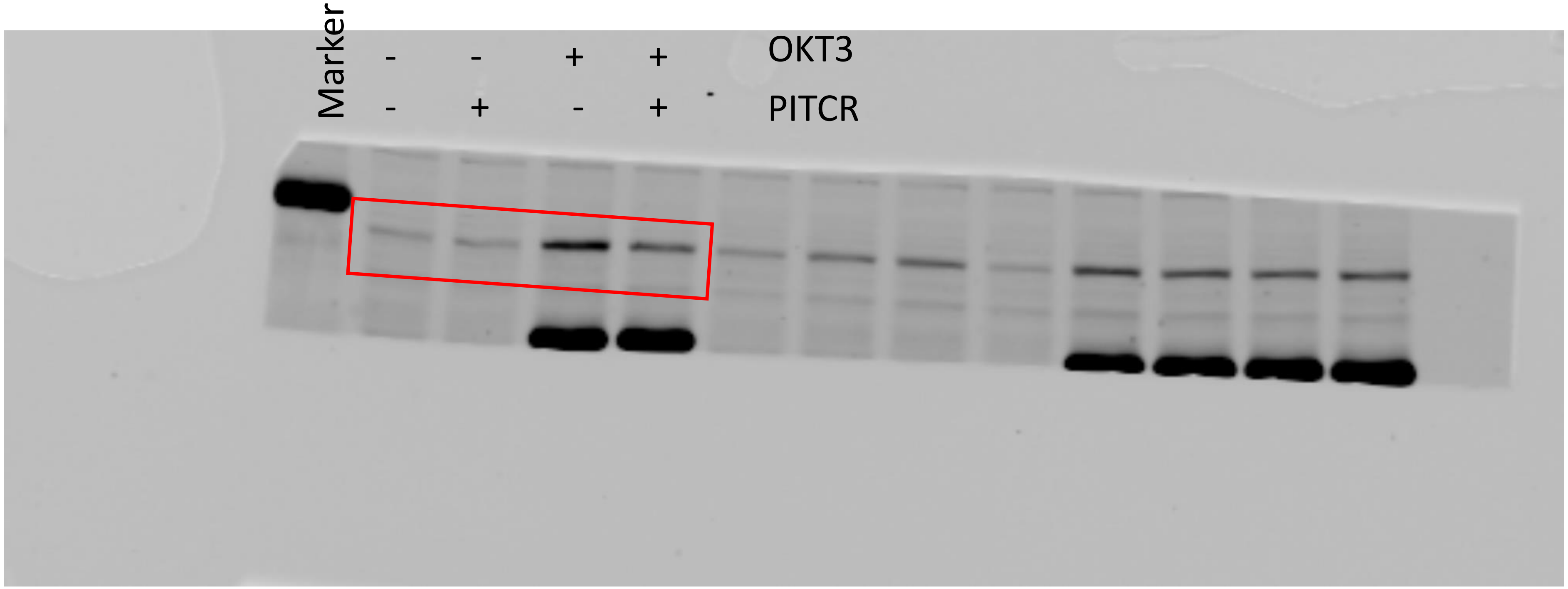

PLCγ1 (pY783)

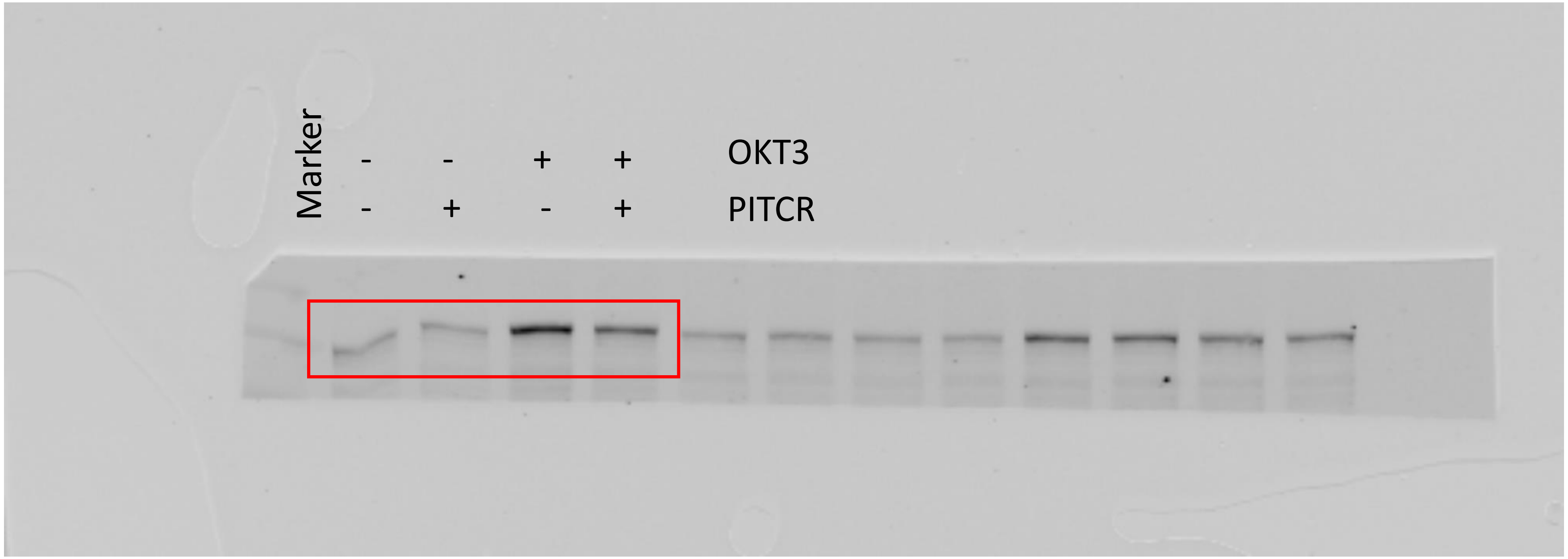

β-actin

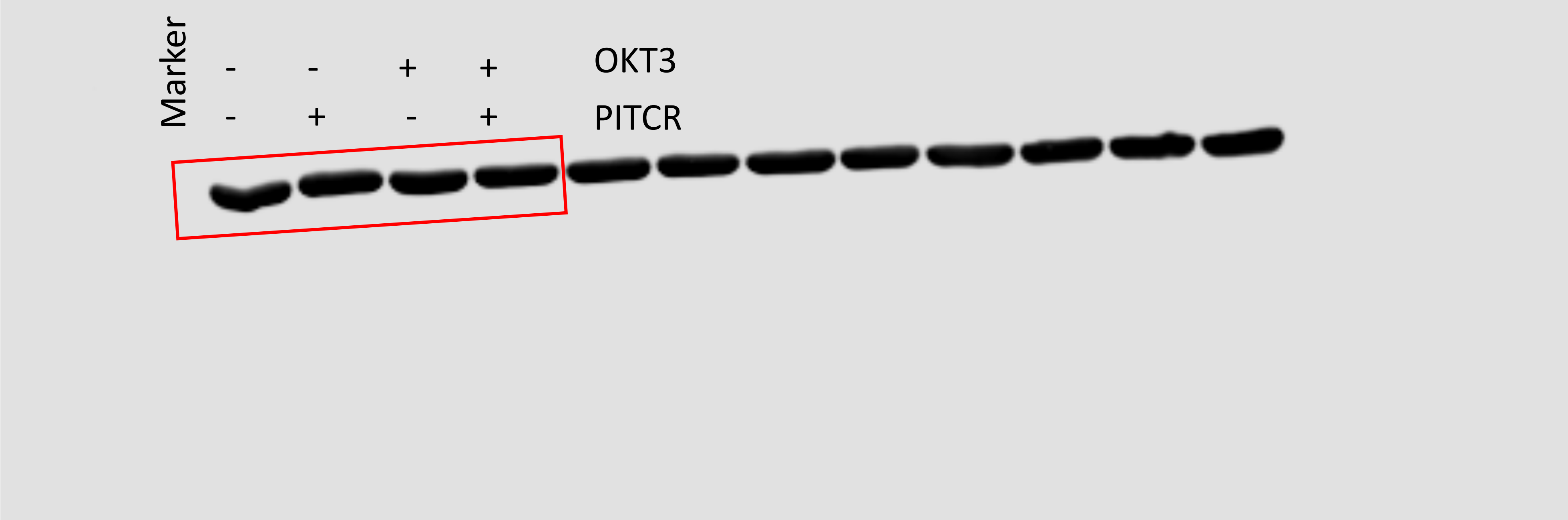

Supplement: Figure 2—source data 1. [file elife-82861-fig2-data1.zip › Figure2_labeled.pdf]
