# Supplementary material for: Allosteric inhibition of the T cell receptor by a designed membrane ligand
Source: eLife. 2023 Oct 5;12:e82861. doi: 10.7554/eLife.82861 (PMC10554751; doi:10.7554/eLife.82861)
Supplement: Figure 2—figure supplement 2—source data 1. [file elife-82861-fig2-figsupp2-data1.zip › Figure2_Figure_supplementary_2_labeled.pdf]

Lck (pY394)

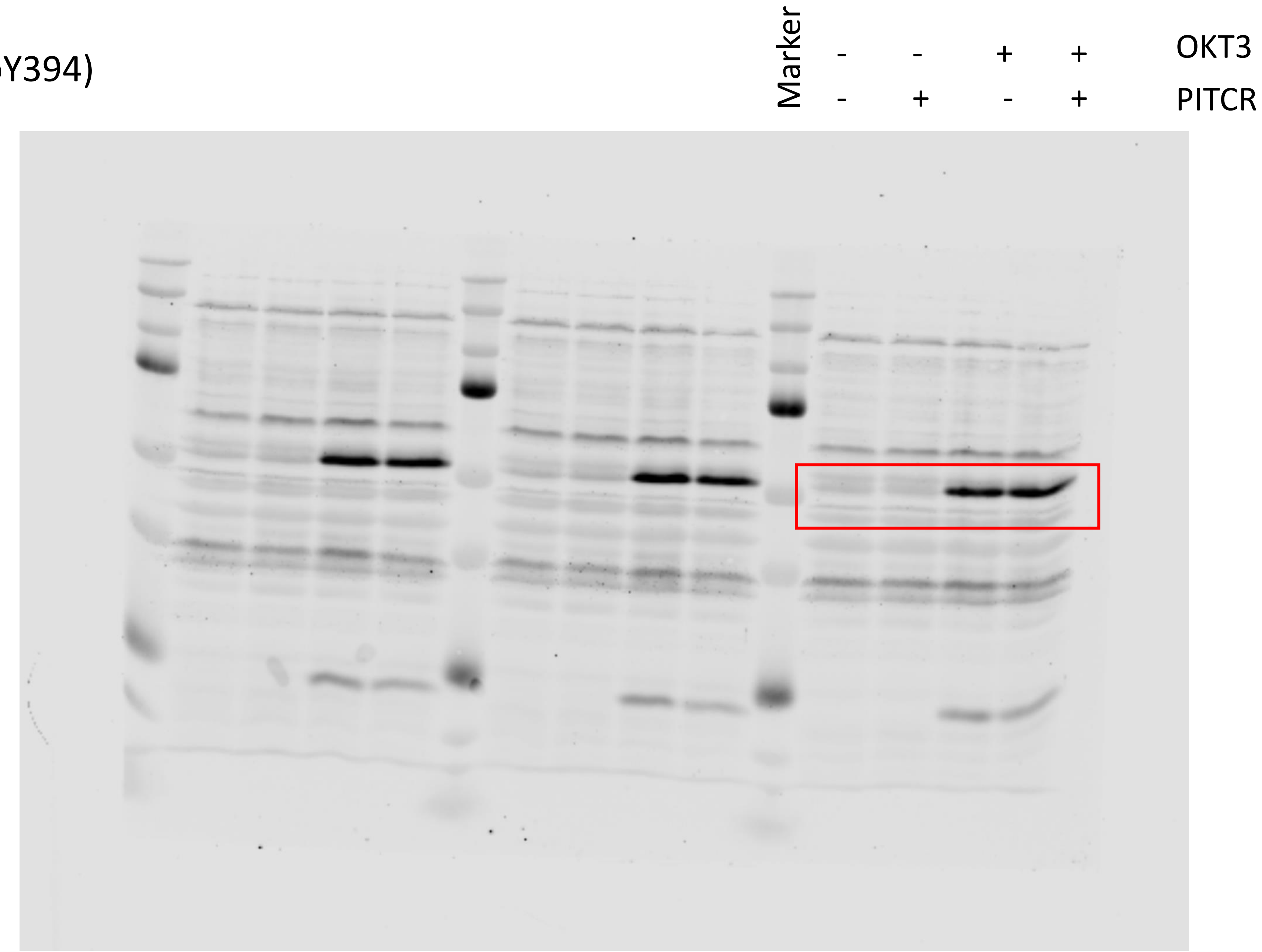

Lck (pY505)

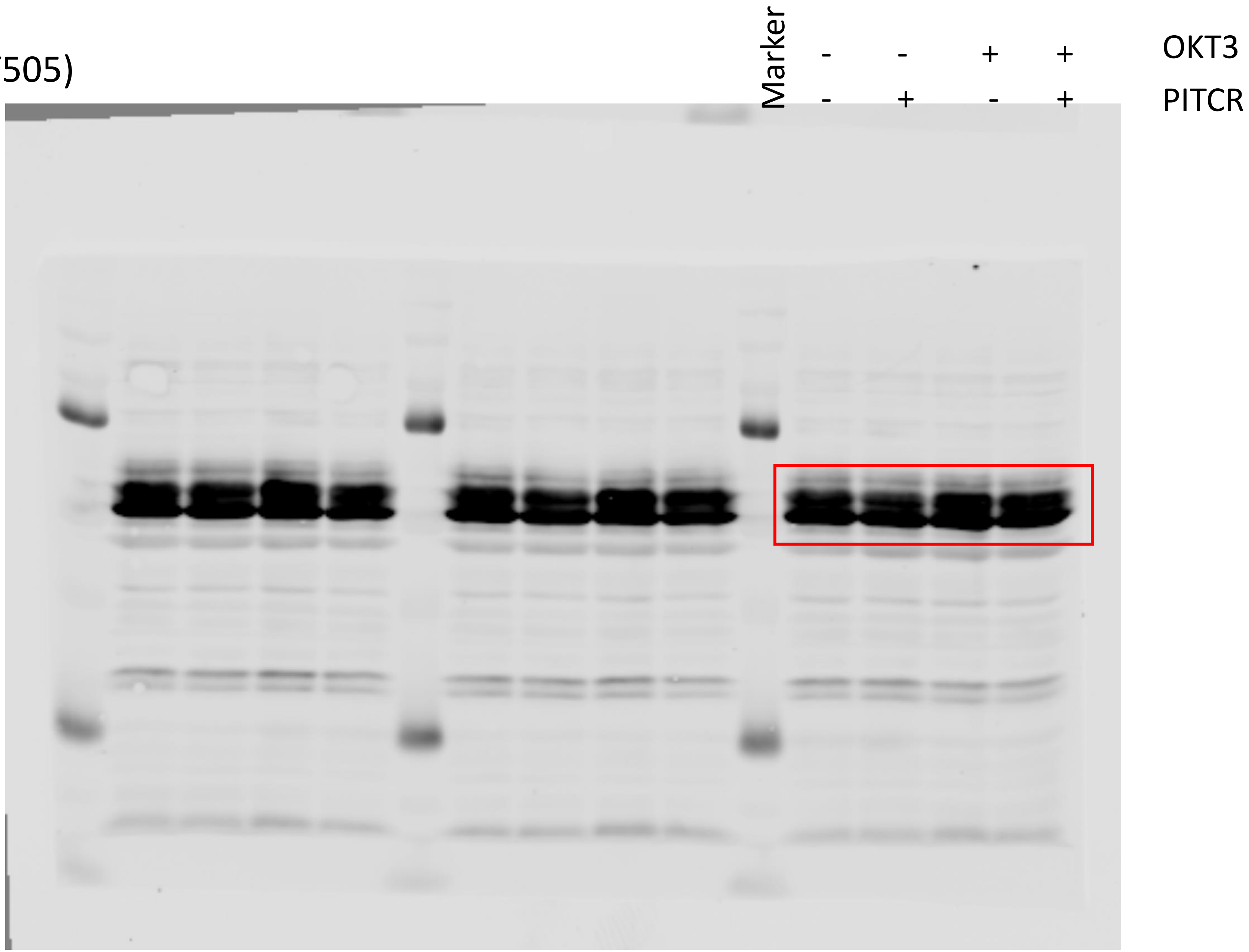

Lck (total)

Marker	-	-	+	+	OKT3
	-	+	-	+	PITCR

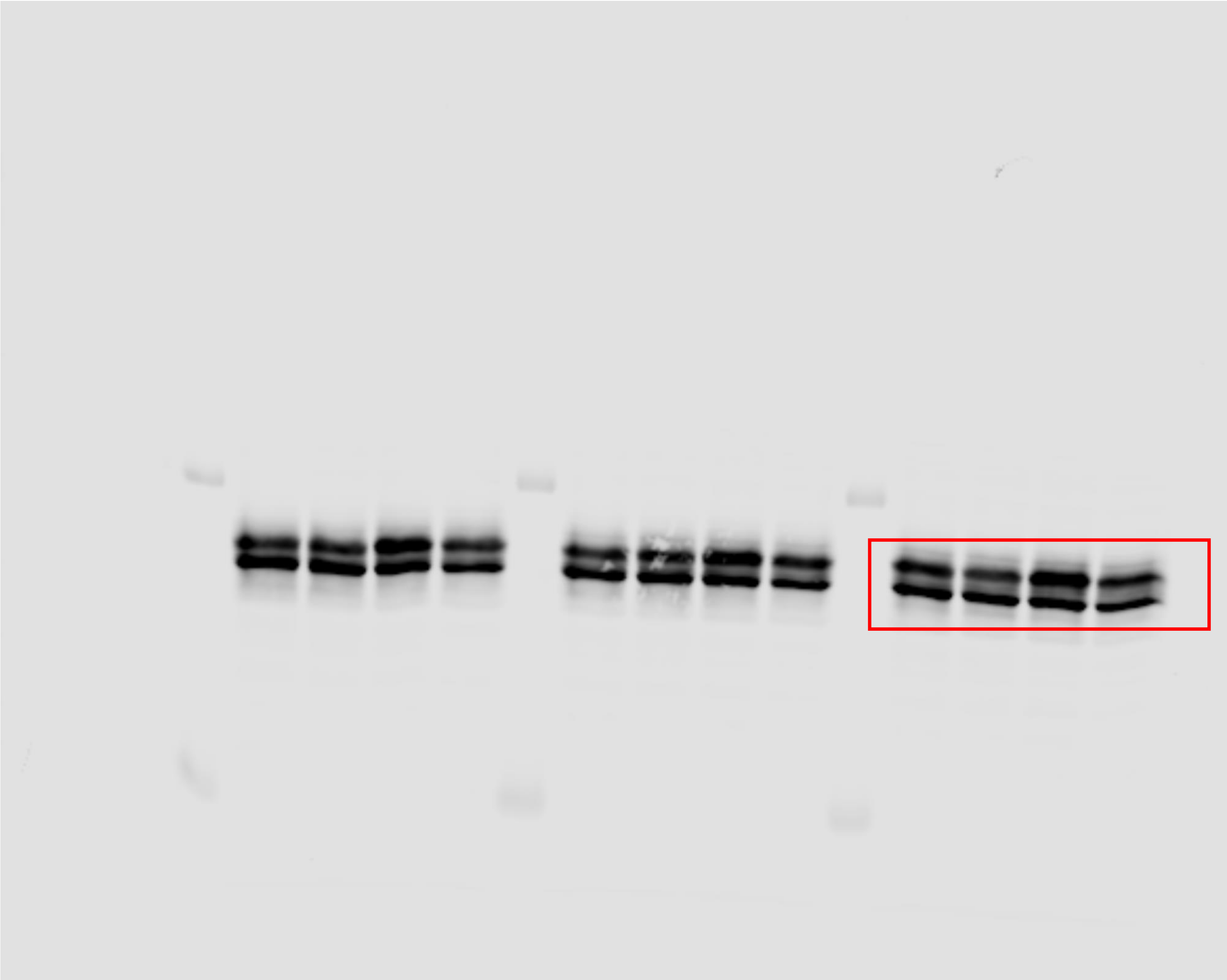

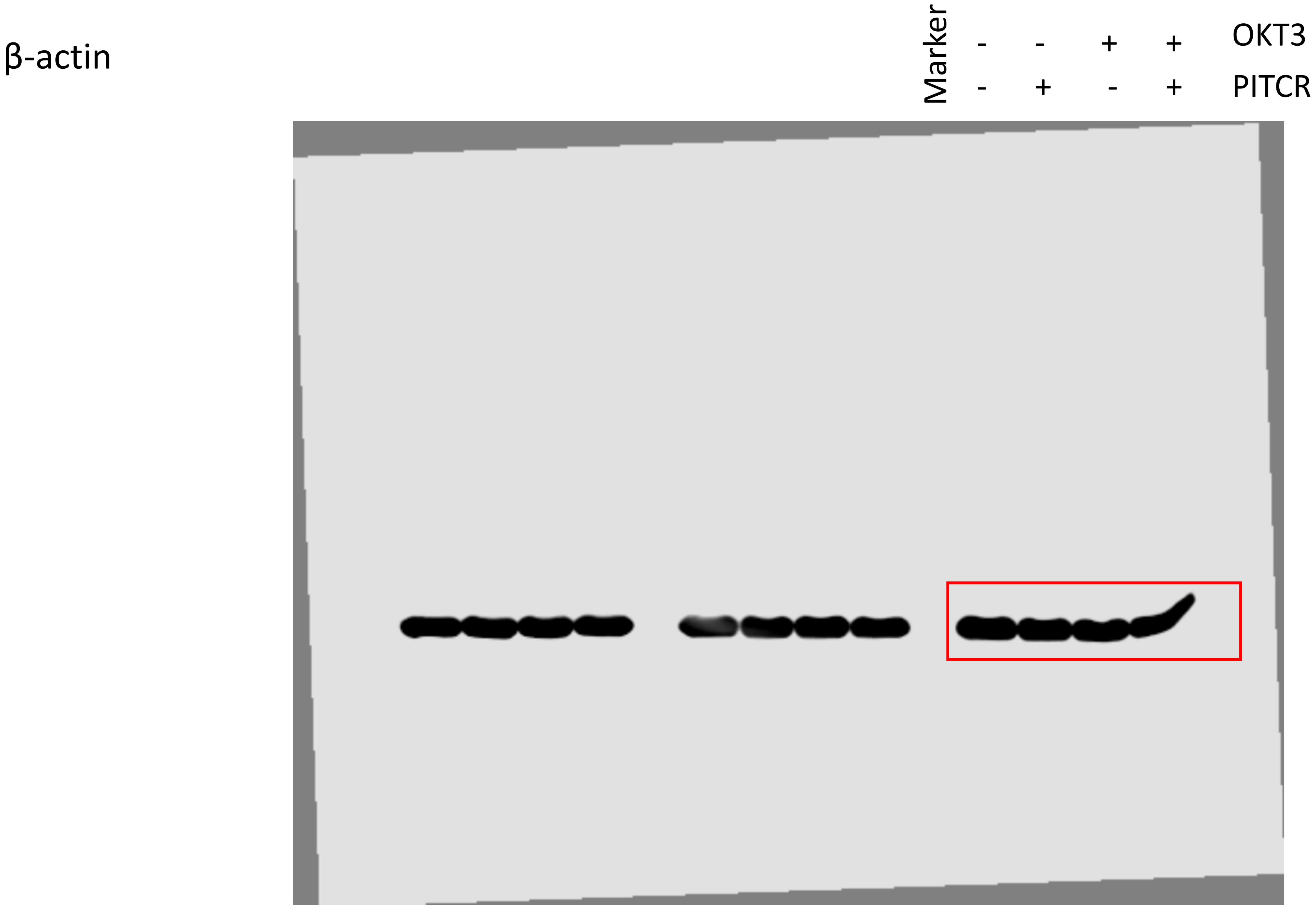
β-actin
Marker
- - + + OKT3
- + - + PITCR
