# Supplementary material for: Allosteric inhibition of the T cell receptor by a designed membrane ligand
Source: eLife. 2023 Oct 5;12:e82861. doi: 10.7554/eLife.82861 (PMC10554751; doi:10.7554/eLife.82861)

Figure 7

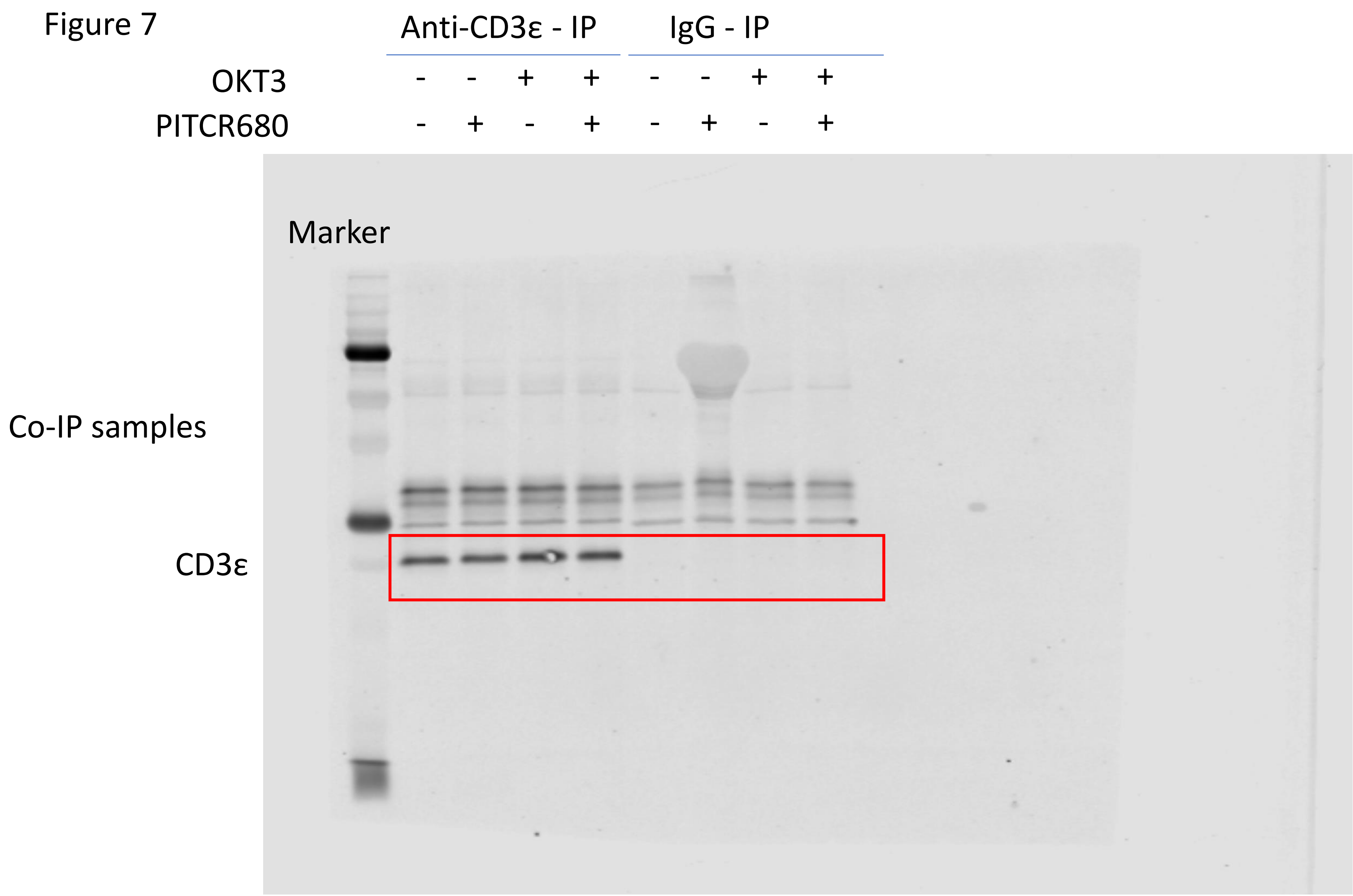

Co-IP samples

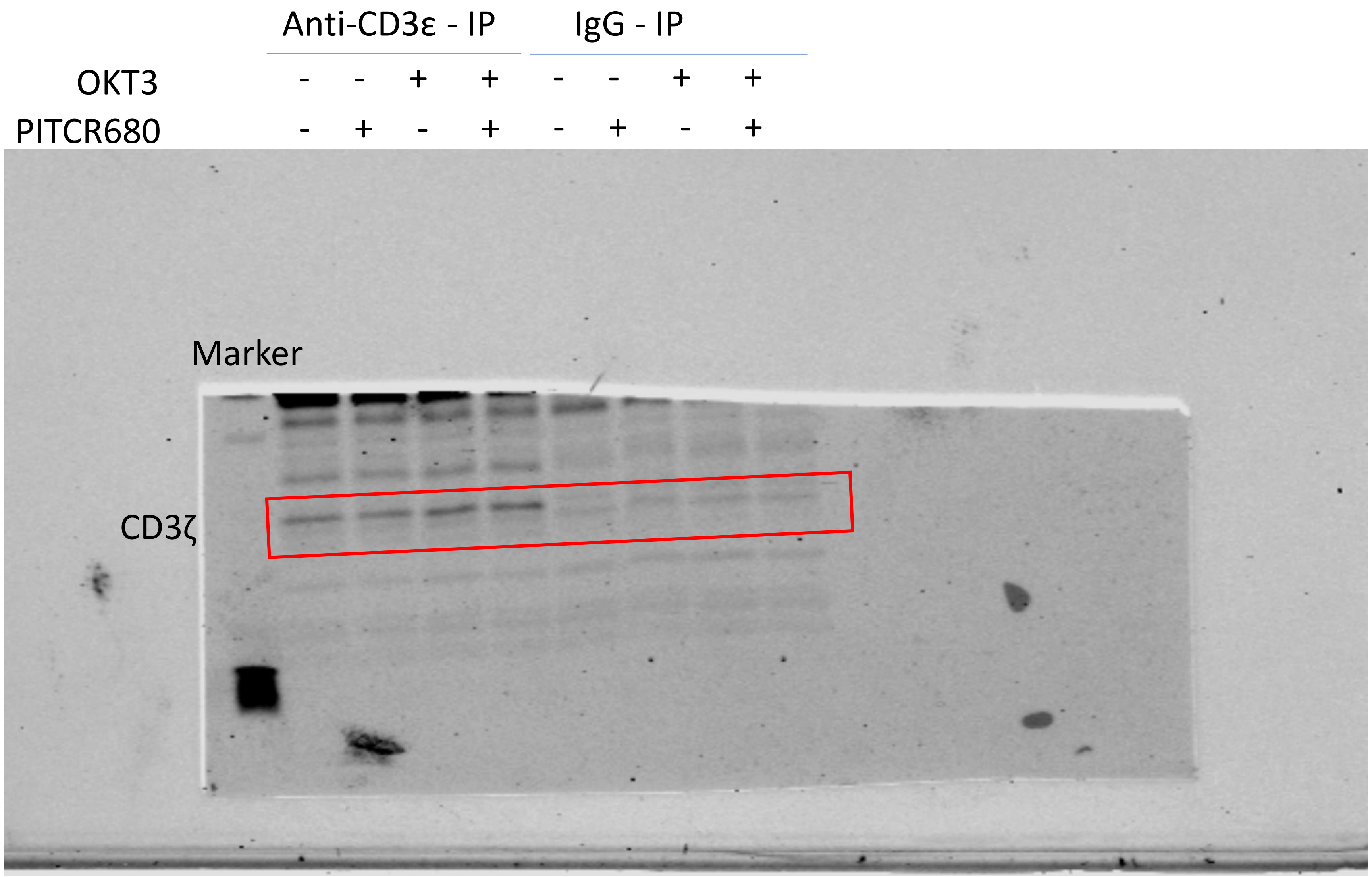

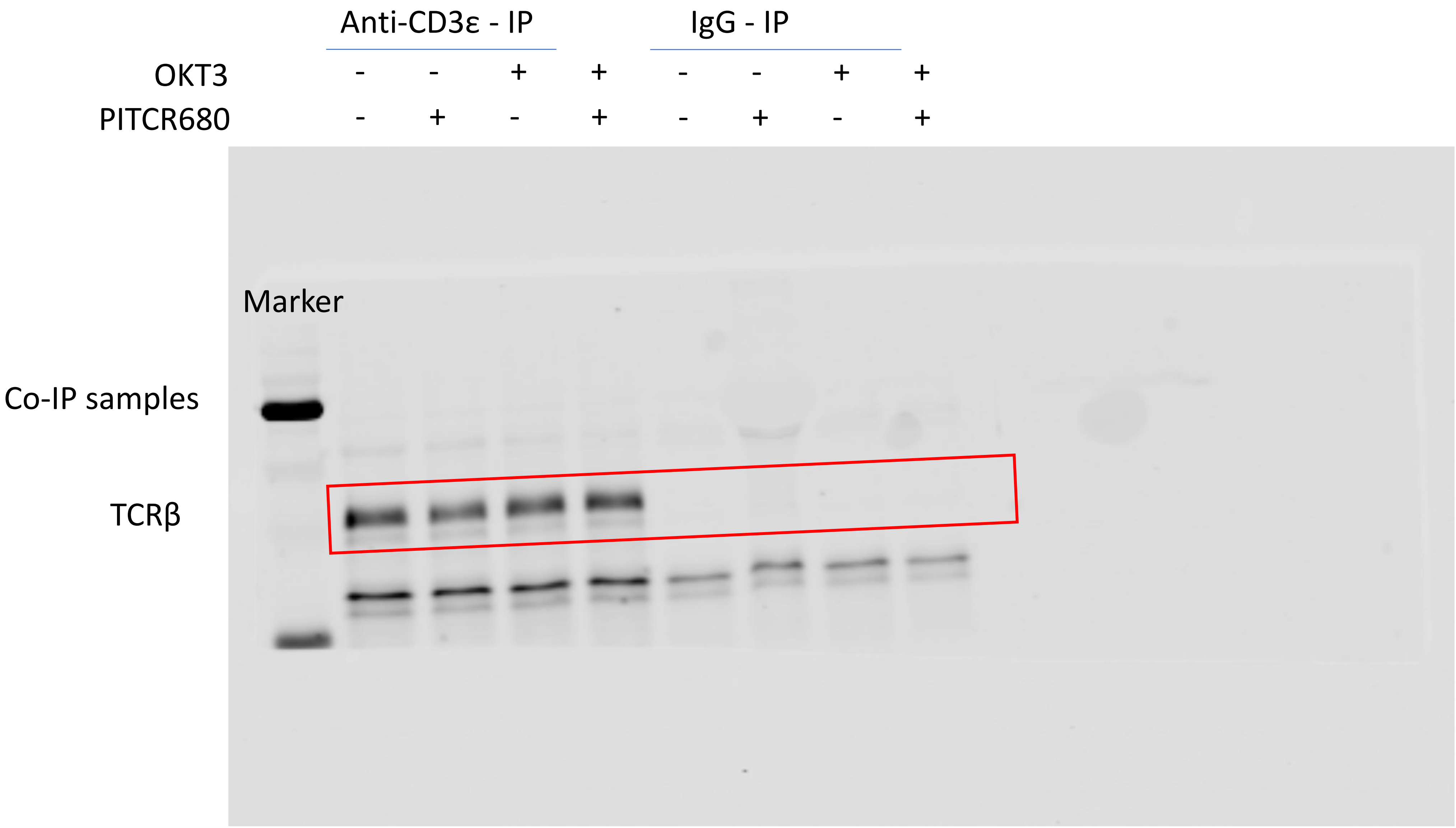

Anti-CD3ε - IP
IgG - IP
OKT3 - - + + - - + +
PITCR680 - + - + - + - +
Marker
Co-IP samples
TCRβ

Whole lysate samples

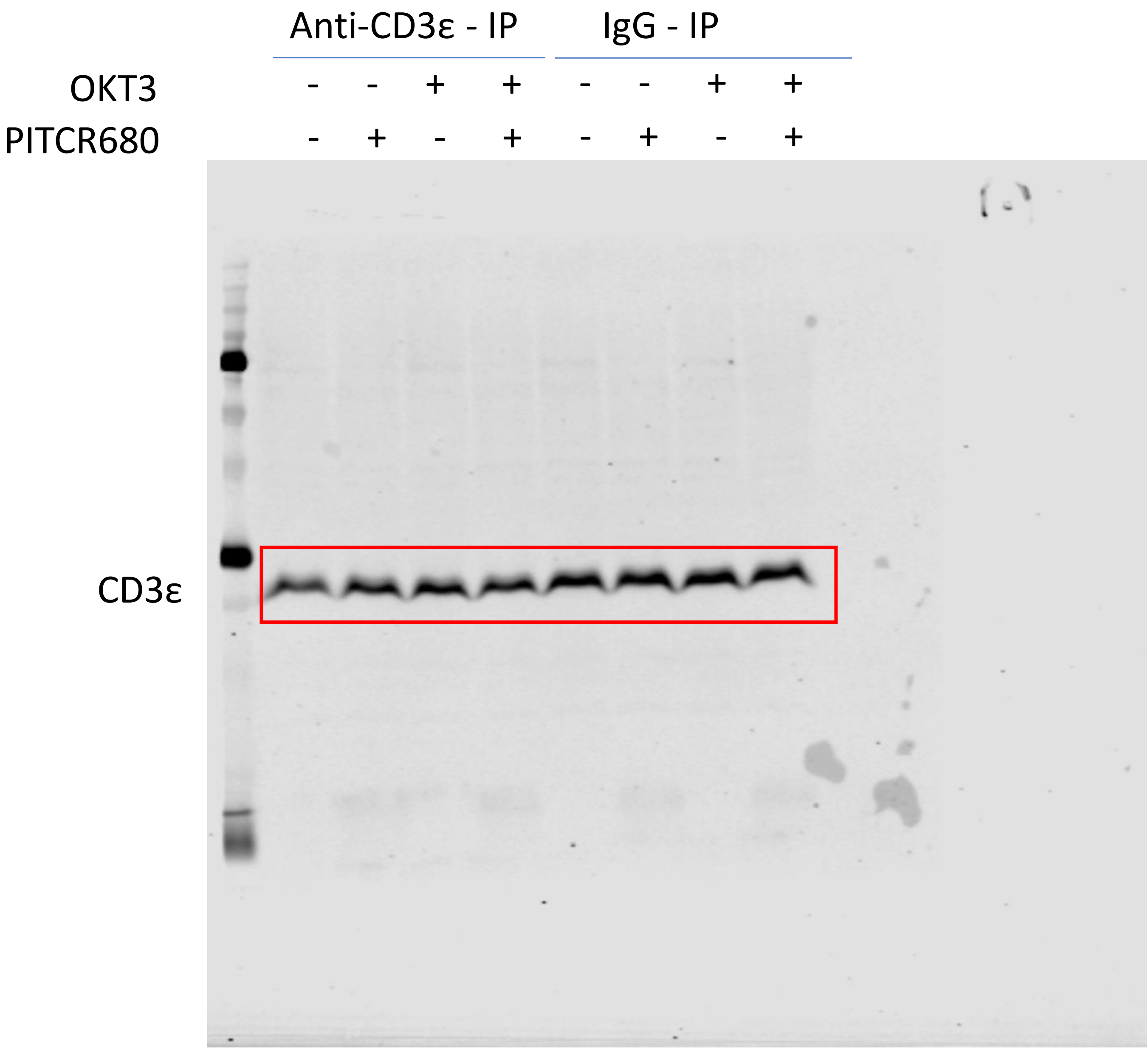

Whole lysate - CD3ζ

	Anti-CD3ε - IP				IgG - IP			
OKT3	-	-	+	+	-	-	+	+
PITCR680	-	+	-	+	-	+	-	+

CD3ζ

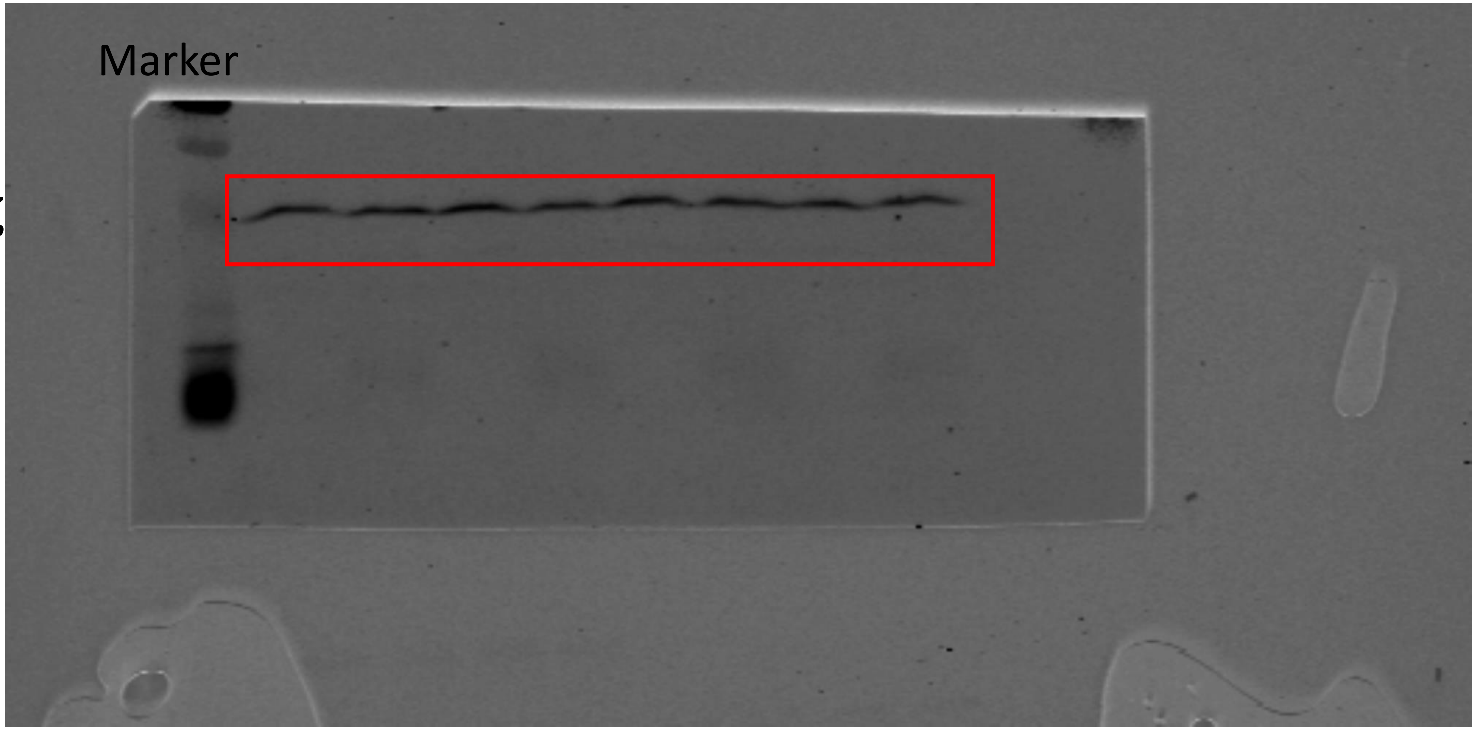

Whole lysate - TCRβ

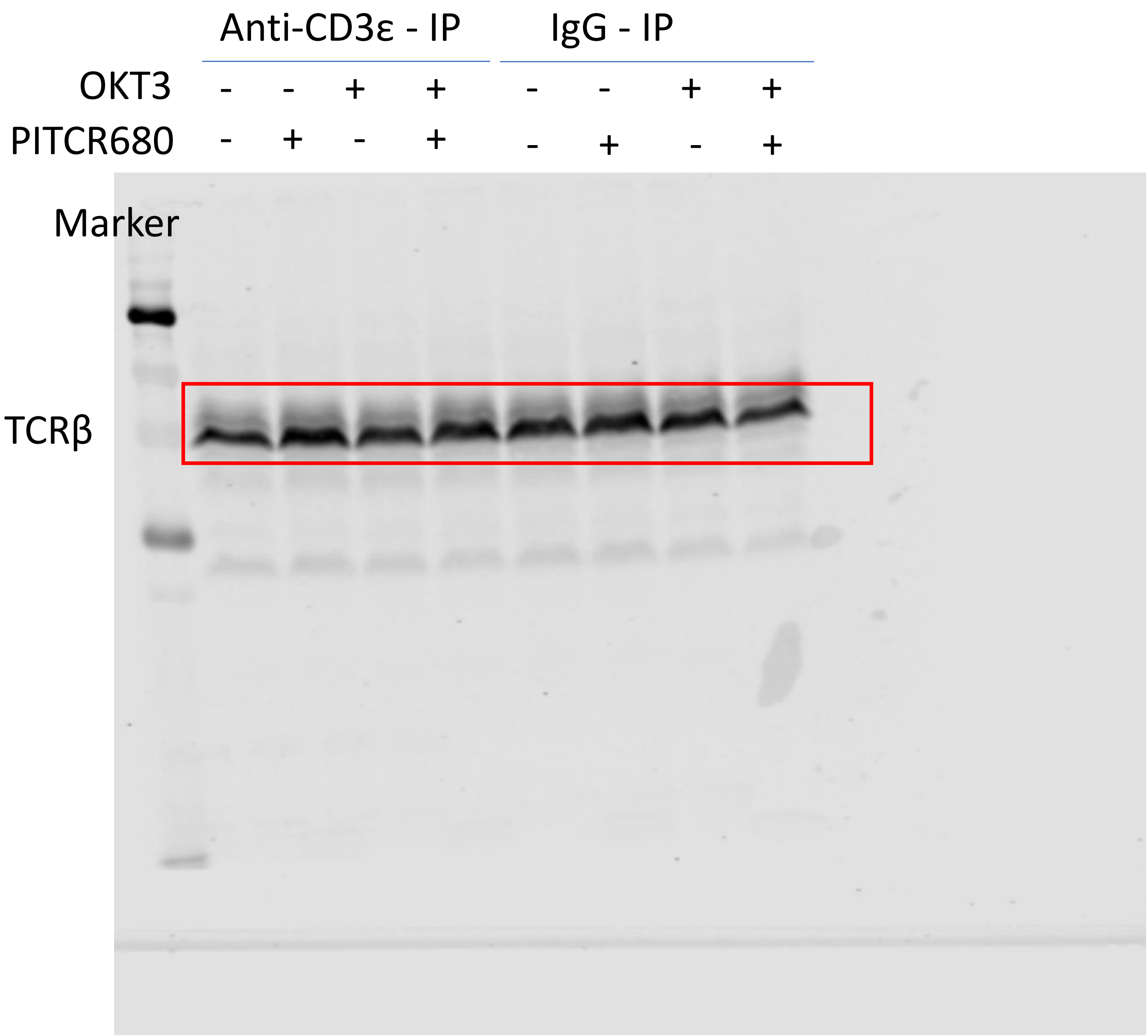

Whole lysate

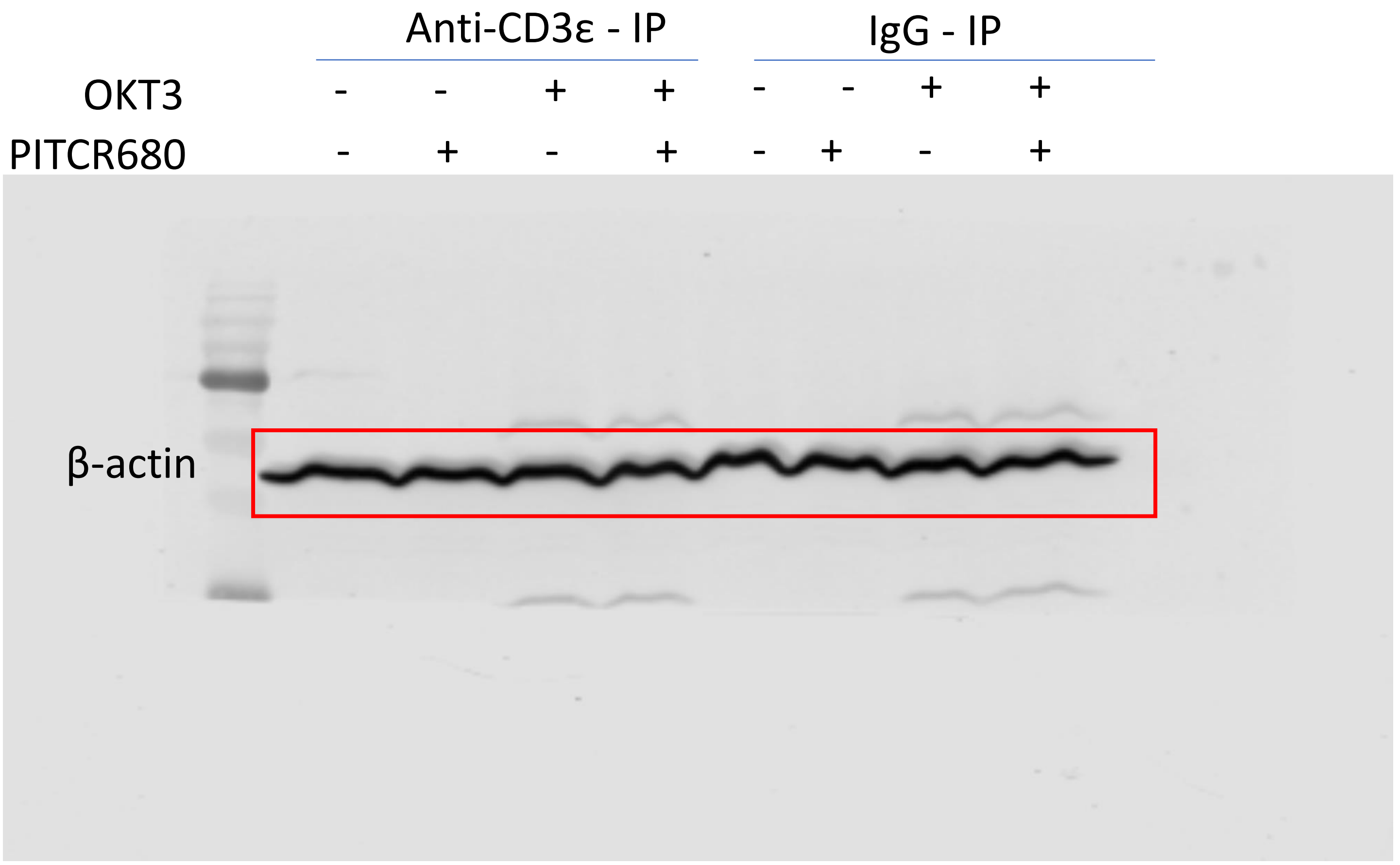

co-IP – PITCR top-panel (left)

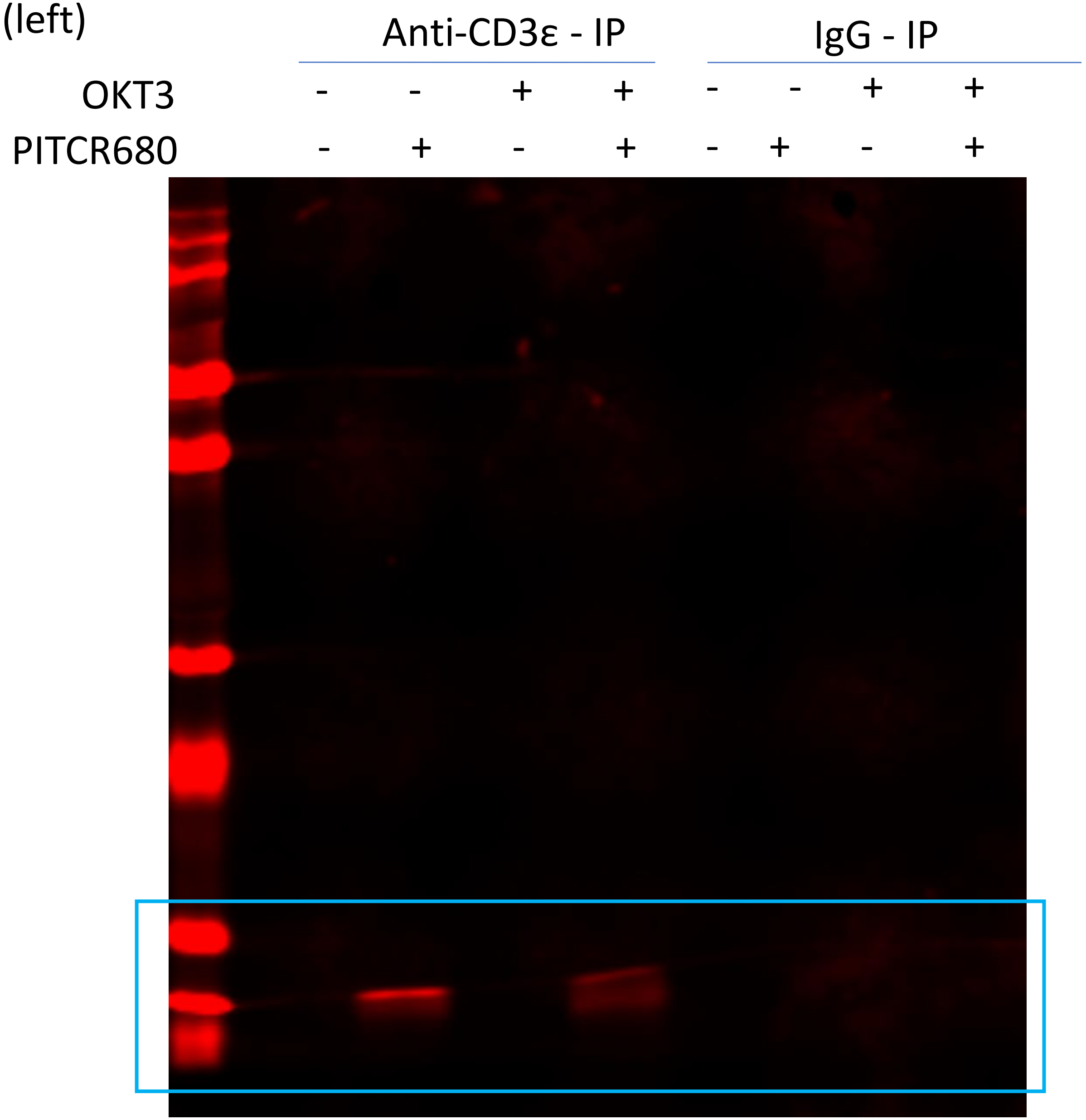

co-IP – marker top-panel (right)

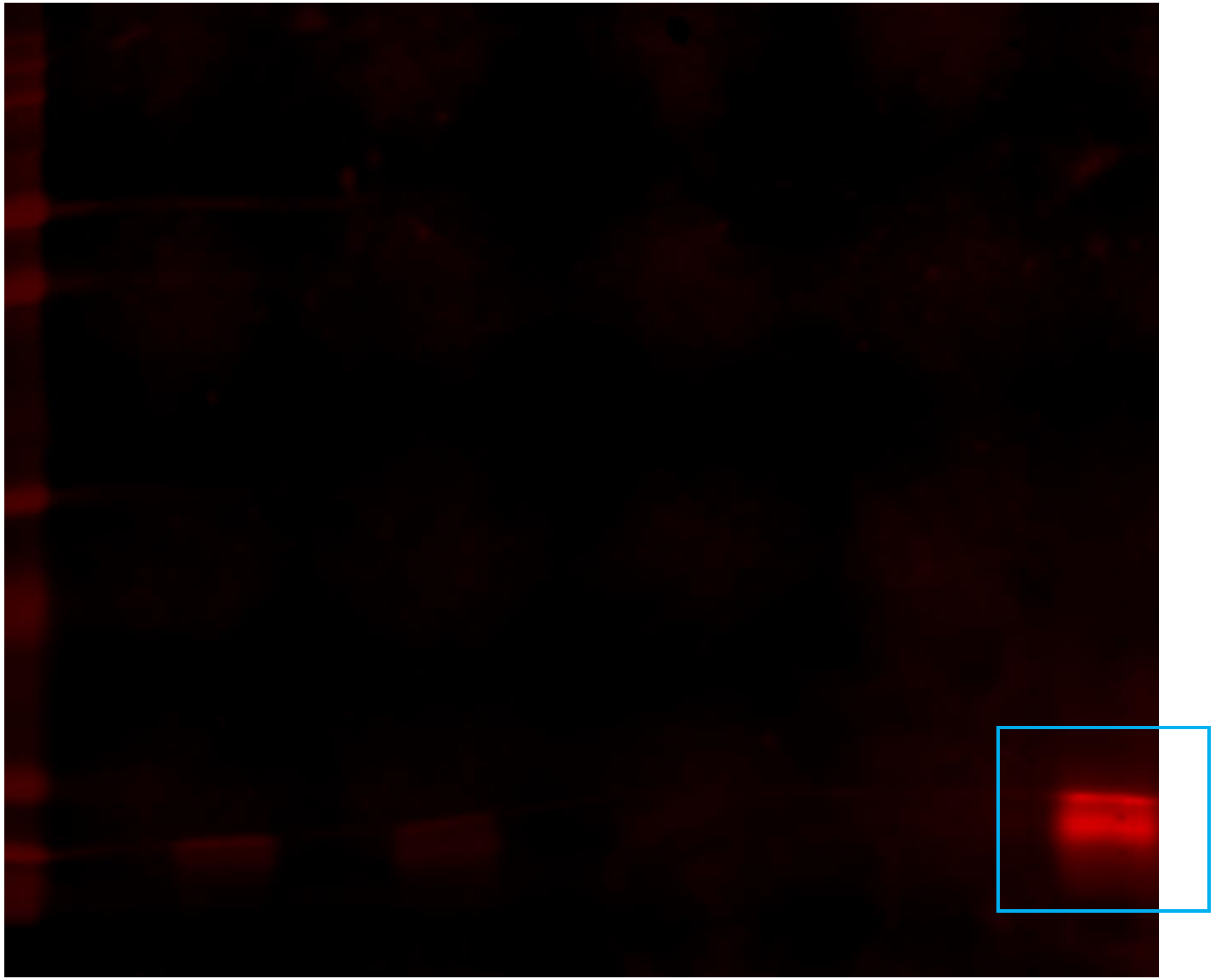

Supplement: Figure 7—source data 1. [file elife-82861-fig7-data1.zip › Figure7_labeled.pdf]
