# Supplementary material for: Allosteric inhibition of the T cell receptor by a designed membrane ligand
Source: eLife. 2023 Oct 5;12:e82861. doi: 10.7554/eLife.82861 (PMC10554751; doi:10.7554/eLife.82861)
Supplement: Figure 8—source data 1. [file elife-82861-fig8-data1.zip › Figure8_labeled.pdf]

Figure 8

IP-(DDM)-CD3ζ

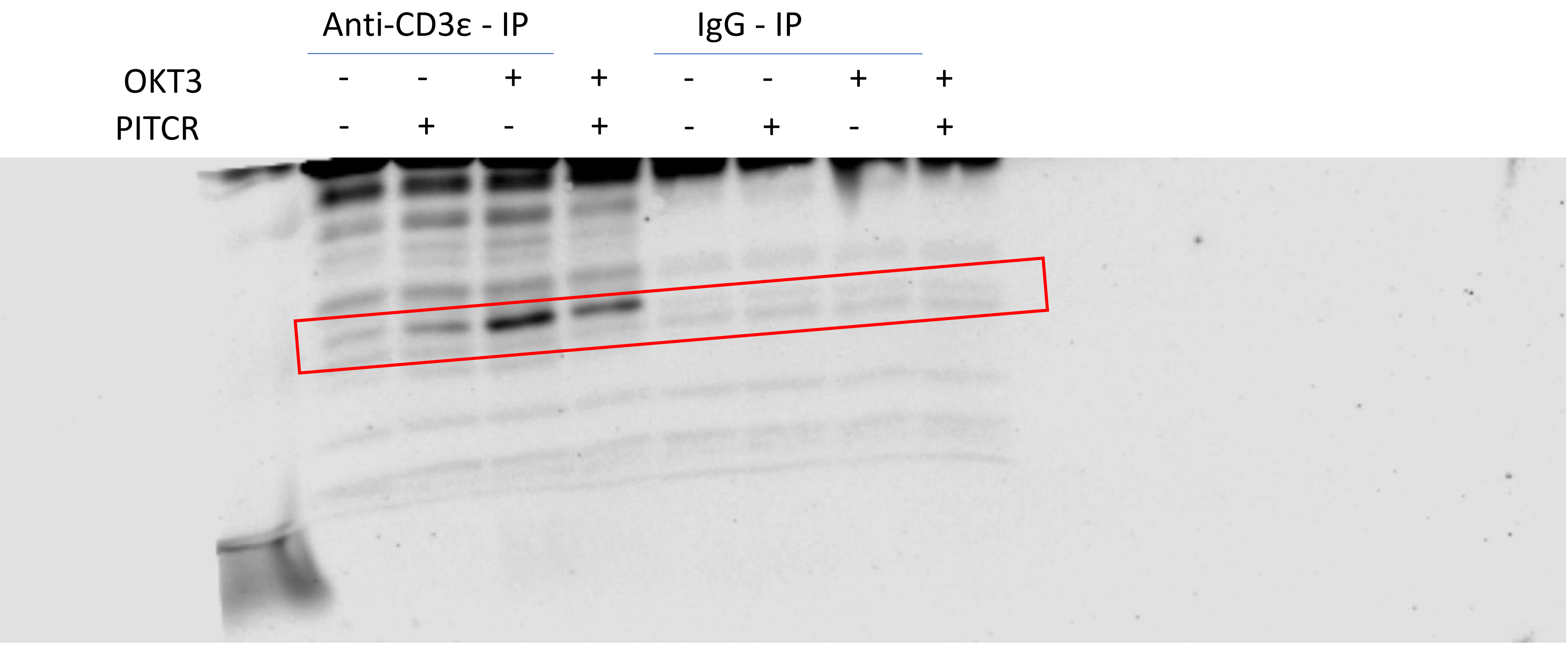

IP-(DDM)-TCRβ

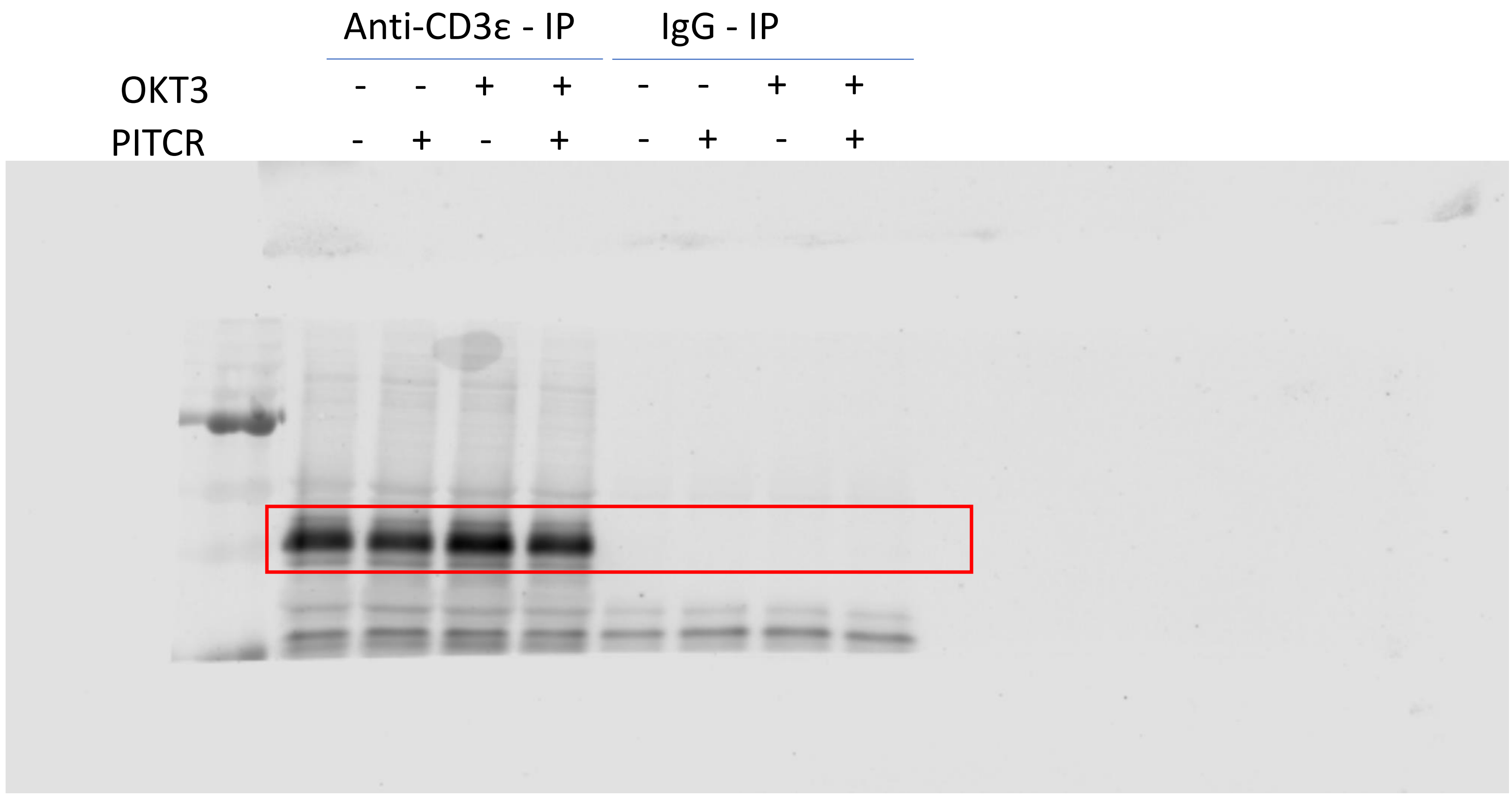

IP-(DDM)-CD3ε

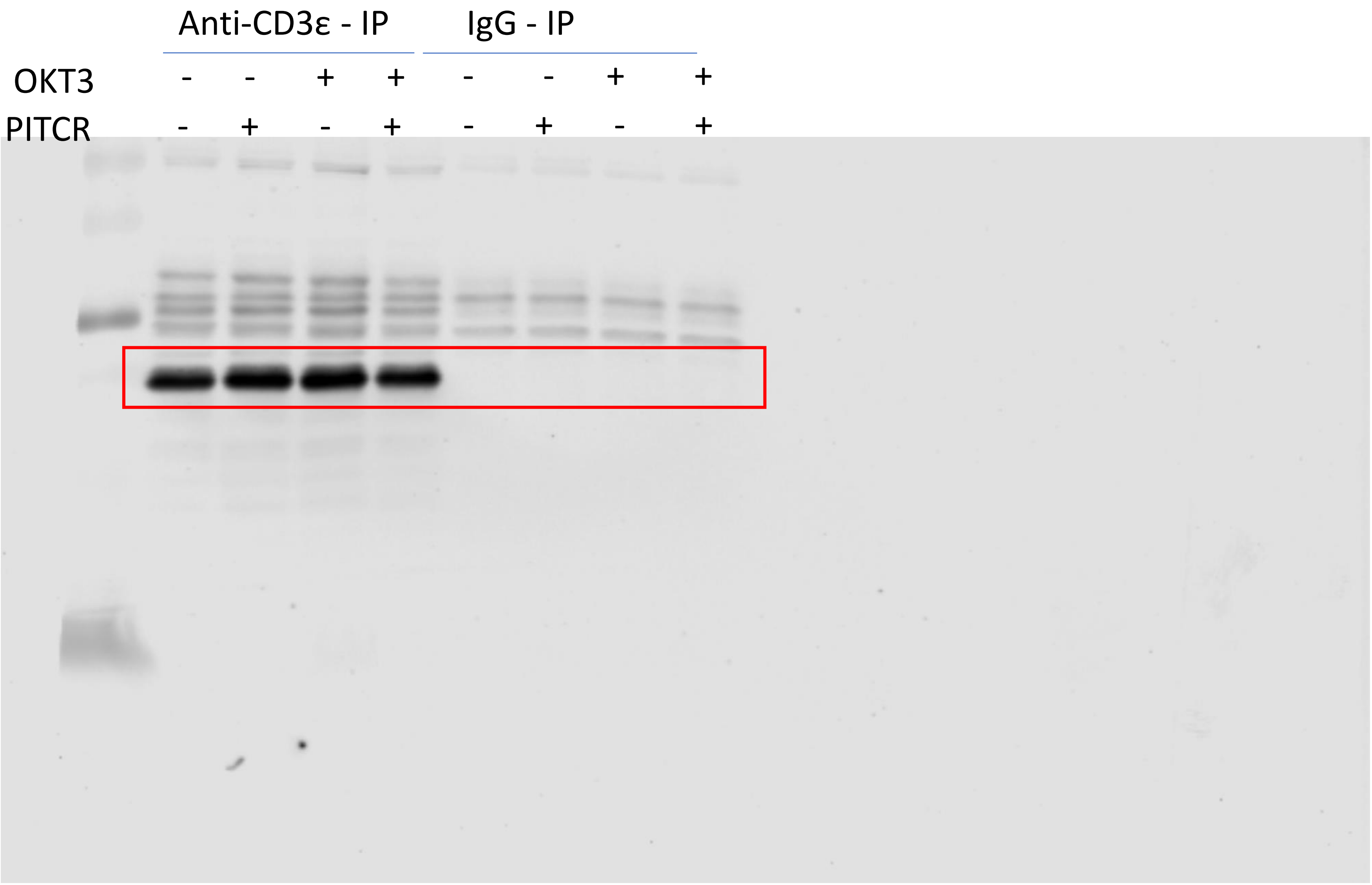

Whole lysate-CD3ζ

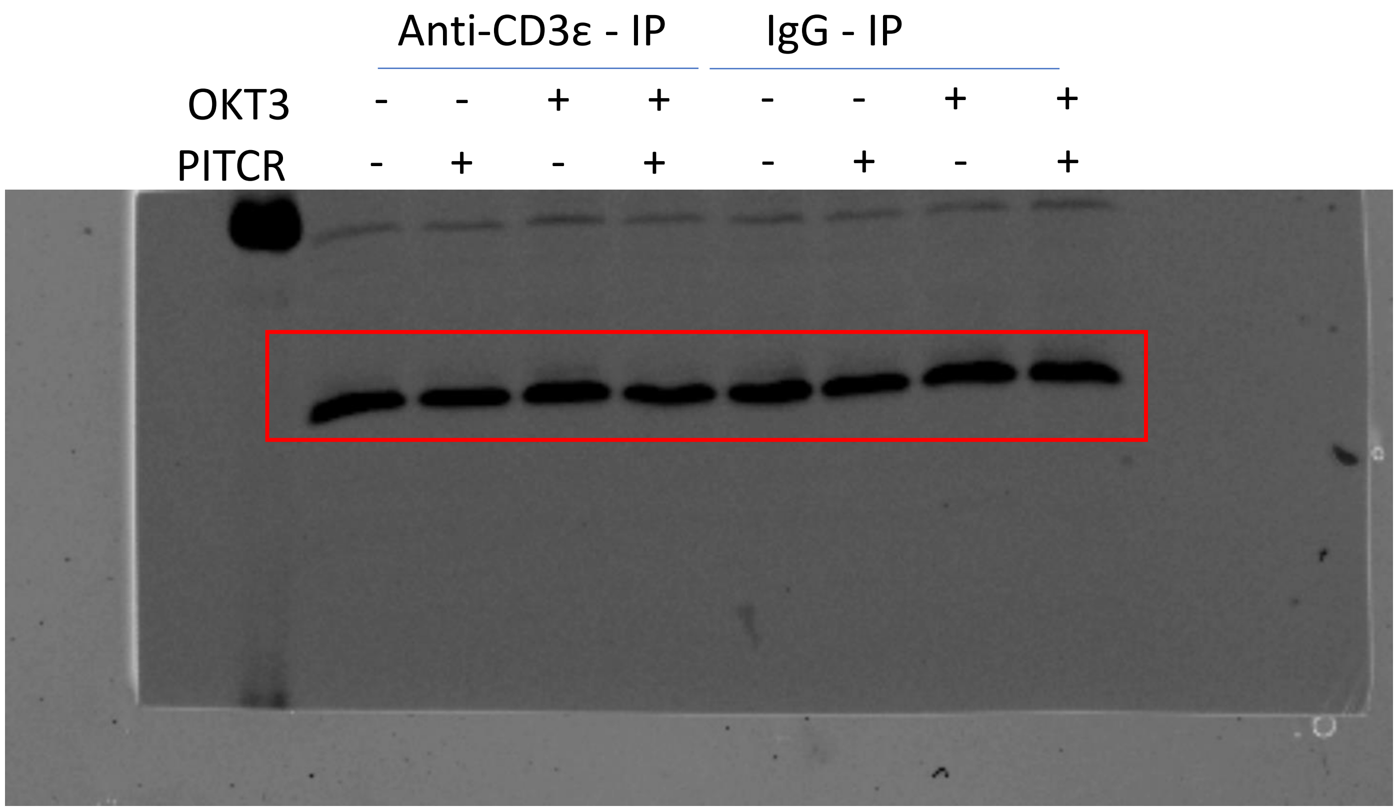

Whole lysate-CD3ε

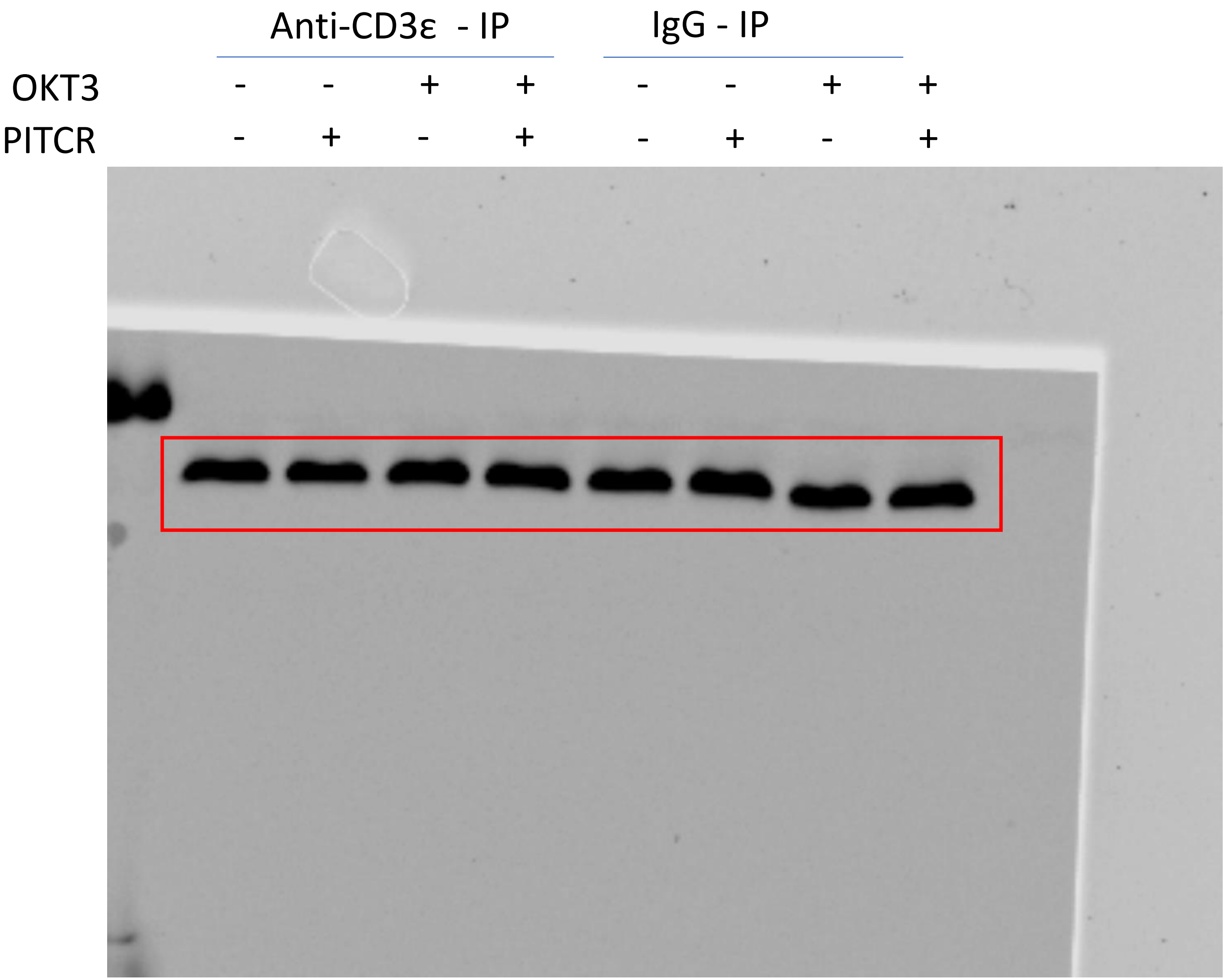

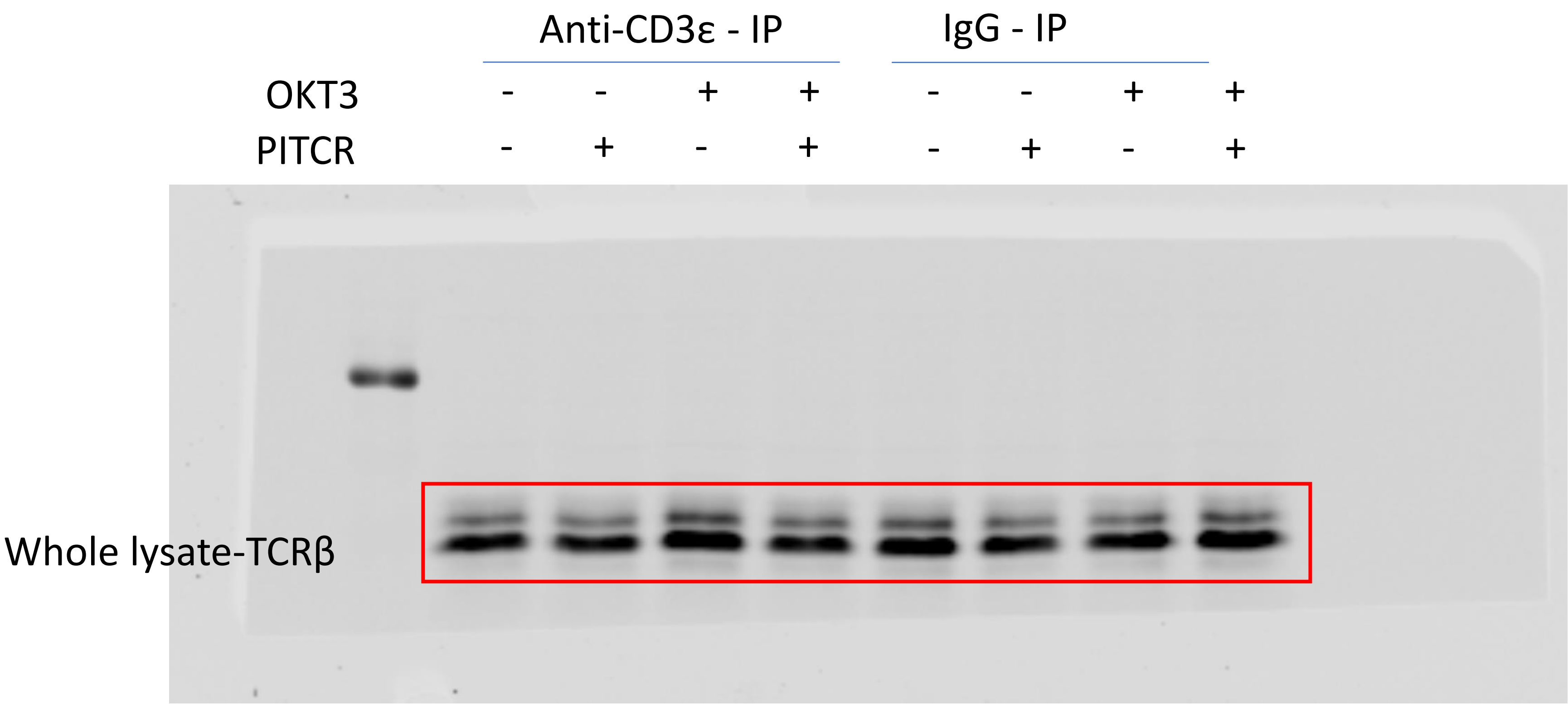
Anti-CD3ε - IP
IgG - IP
OKT3 - - + + - - + +
PITCR - + - + - + - +
Whole lysate-TCRβ

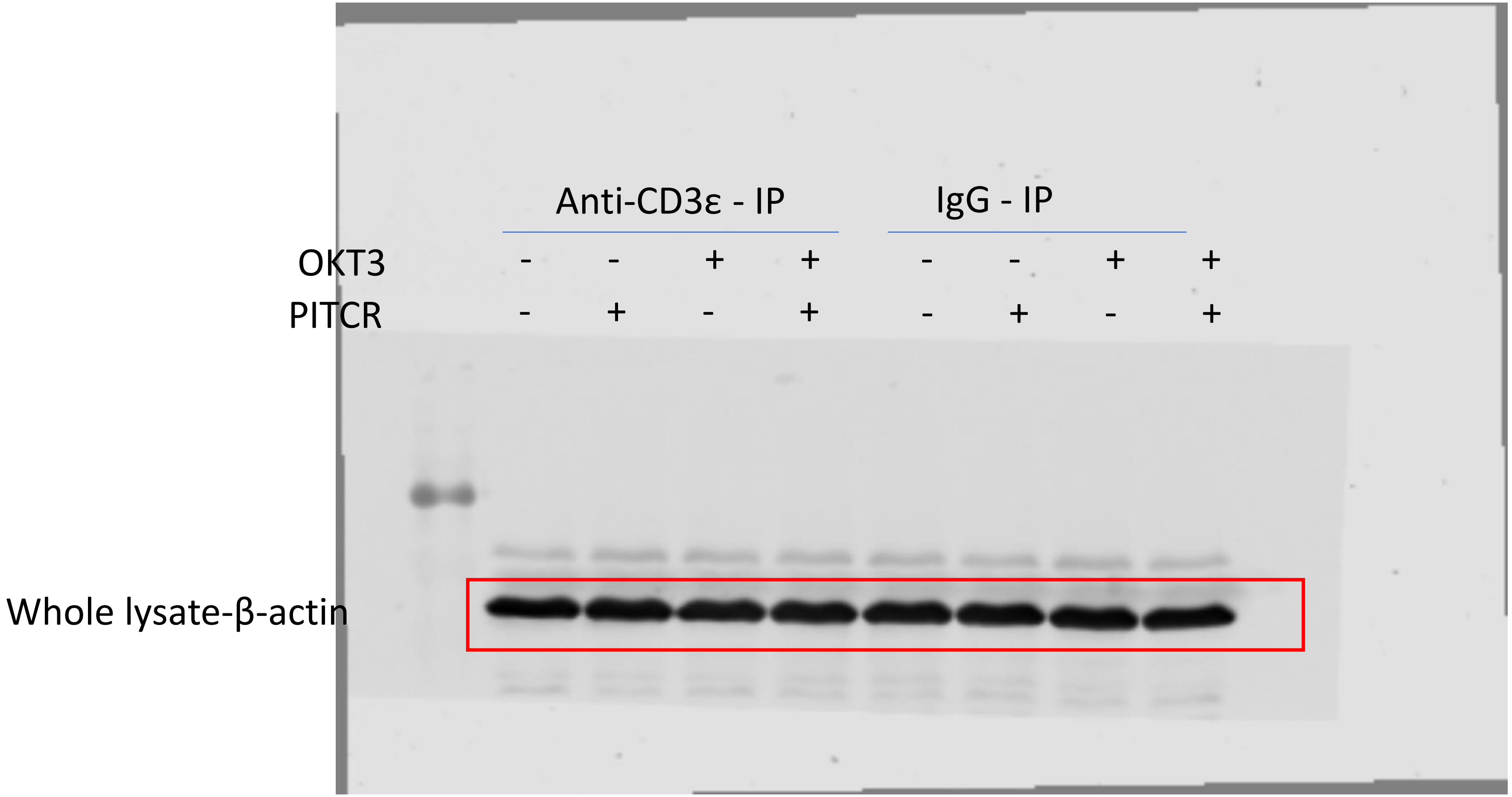
Anti-CD3ε - IP
IgG - IP
OKT3 - - + + - - + +
PITCR - + - + - + - +
Whole lysate-β-actin
