# Supplementary material for: Allosteric inhibition of the T cell receptor by a designed membrane ligand
Source: eLife. 2023 Oct 5;12:e82861. doi: 10.7554/eLife.82861 (PMC10554751; doi:10.7554/eLife.82861)
Supplement: Figure 9—source data 1. — (A) Table listing residues in peptide inhibitor of T cell receptor (PITCR) and PITCRG41P that closely interact with one or both zeta chains and the corresponding interaction distance. Zeta chains are labeled A and B as in Figure 9B. (B) Table listing the maximum displacement distance for α-carbons induced in zeta chain by the indicated peptide, measured by superimposition of the TCRα chain in the AlphaFold model of TCR alone and the model of TCR associated with each peptide. The root-mean-square deviation (RMSD) value for the zeta chains is also listed, calculated by superimposition of the TCRα chain in the AlphaFold model of TCR alone and the model of TCR associated with each peptide. Note that both zeta chains are displaced to a greater extent by PITCR than by the other peptides. [file elife-82861-fig9-data1.pdf]

A

Amino acid in peptide	PITCR contacts with CD3ζ	PITCR interaction distance	PITCRG41P contacts with CD3ζ	PITCRG41P interaction distance
Asp1	ZA Tyr33*	3.37Å	none	-
Ser5	ZB Cys32*	3.60Å	none	-
Tyr6	ZB Asp28**<br>ZB Leu26**	3.37Å<br>2.67Å	none	-
Tyr15	ZA Thr47*	2.76Å	ZA Thr47*<br>ZB Tyr42*	2.61Å<br>2.79Å
Thr20	ZB Tyr42*	2.89Å	none	-
Glu30	ZB Arg57*	2.67Å	ZB Ser56*	2.72Å

*Interaction with sidechain atoms in TCR
**Interaction with backbone atoms in TCR

B

Model 1		Model 2	Maximum displacement of Zeta A α-carbon	Maximum displacement of Zeta B α-carbon	RMSD for Zeta A + Zeta B + TCRα
TCR	vs	TCR + PITCR	6.01Å	8.20Å	1.876Å
TCR	vs	TCR + PITCRG41P	3.72Å	3.62Å	1.179Å
TCR	vs	TCR + TYPE7	4.25Å	3.97Å	1.044Å
TCR	vs	TCR + pHLIP	2.49Å	1.86Å	0.438Å
